# No Switching Cooperativity between Coordinated Azo Ligands on Complexes Having {M^II^(phosphane-κ^2^*P*)}^2+^ (M = Pd, Pt) Scaffolds

**DOI:** 10.1021/acs.inorgchem.4c02169

**Published:** 2024-08-21

**Authors:** Ot Raïch Panisello, Jesús Jover, Cristina Puigjaner, Montserrat Ferrer, Manuel Martínez

**Affiliations:** † Secció de Química Inorgànica, Departament de Química Inorgànica i Orgànica, Universitat de Barcelona, Martí i Franquès 1-11, 08028 Barcelona, Spain; ‡ Institut de Química Teòrica i Computacional (IQTCUB), Universitat de Barcelona, 08028 Barcelona, Spain; § Unitat de Difracció de RX, Centres Científics i Tecnològics de la Universitat de Barcelona (CCiTUB), Universitat de Barcelona, Solé i Sabarís 1-3, 08028 Barcelona, Spain; ∥ Institute of Nanoscience and Nanotechnology (IN2UB), Universitat de Barcelona, 08028 Barcelona, Spain

## Abstract

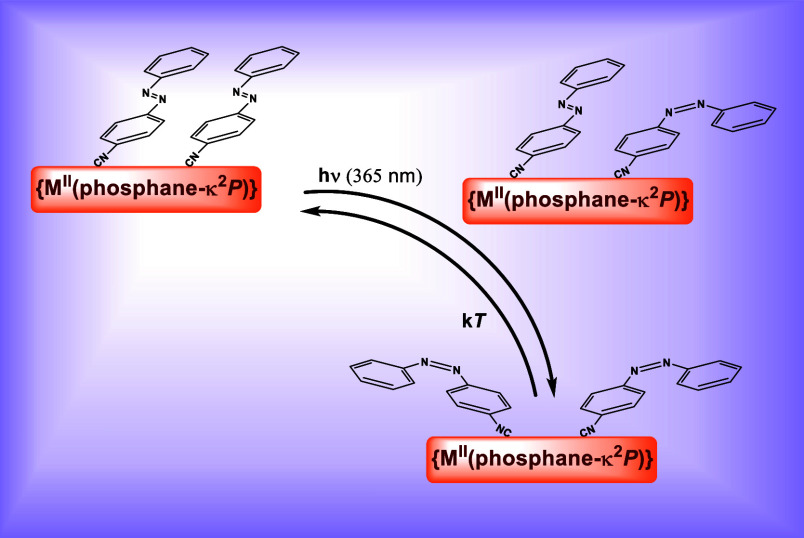

A series of square-planar palladium and platinum compounds with *cis*-blocking phosphanes and terminal azobenzene ligands [M(dppp)(azo)_2_](OTf)_2_ (azo = CN(C_6_H_4_)-N=N-(C_6_H_4_)CN (**iso-cyano**), CN(C_6_H_4_)-N=N-(C_6_H_5_) (**iso-Ph**)) and [{M_2_(tpbz)}(azo)_4_](OTf)_4_ (azo = CN(C_6_H_4_)-N=N-(C_6_H_5_) (**iso-Ph**)) have been synthesized and fully characterized. Similarly to the uncoordinated ligands, the new coordination compounds have shown to be photochemically active with respect to their trans-to-cis isomerization process. Their cis-to-trans back spontaneous reaction have been studied as a function of solvent, temperature and pressure and the corresponding activation parameters determined in order to investigate the mechanism of these transformations. The results obtained are indicative of the operation of a rotational mechanism with no cooperativity between the azo ligands attached to the same metal. Density functional theory calculations have been carried out in order to estimate the relative energies of the different photoisomers for the theoretical interpretation of the experimental data.

## Abstract 2

Square-planar Pd^II^ and Pt^II^ compounds with dangling azobenzene ligands are shown to be photochemically (trans−cis) active with no cooperativity between the azo ligands; the back spontaneous cis−trans reaction has been found to occur via and enhanced rotational isomerization mechanism.

## Introduction

**Scheme 1:**
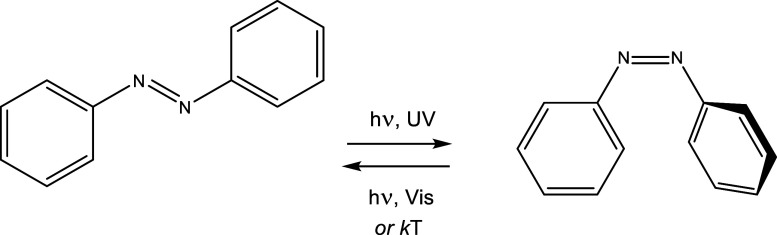


Azobenzene derivatives are historical compounds with a robust and well-known photochemical/thermal isomerization switching around the central diazene unit (Scheme 1).^1^ There are many studies dealing with the photochemical process producing the *cis* isomer, from the *trans* thermodynamically stable form, by means of the π–π* transition at high energies of the molecule, as well as that of the back reaction triggered by the nb–π* transition on illumination at lower energies.^2^

The complementary cis-to-trans thermal spontaneous back isomerization reaction producing the stable *trans* species has been also thoroughly studied, given the importance involved in this process in photoswitching materials.^3,4^ Furthermore, the additional cooperative effects derived from the existence of more than one switching unit in a single compound have also been explored with different outcomes; these include both interesting communication between the units or total independence.^4−7^ This spontaneous process is known to be dependent on a wealth of factors that, ultimately, relate to structural characteristics that favor rotational (charge-separated), or inversional (noncharge-separated) transition states.^8−10^ These structural differences have been mostly tuned by using push–pull functionalization of the diazene-containing molecule, thus achieving a desired switching response time.^11^ Even so, we have demonstrated that the choice of solvent is also a crucial point for the establishment of the isomerization mechanism operating during the process.^12^ In all cases, the comprehensive solvent, temperature and pressure dependence of the isomerization kinetics, including the determination of the thermal (Δ*H*^‡^, Δ*S*^‡^) and pressure (Δ*V*^‡^) activation parameters,^13,14^ has been found to be an ideal tool for this purpose.^15−17^

Coordination of azobenzene derivatives to metal centers is not new, but it had usually involved the nitrogen donors of the diazene moiety to metals,^18^ leading to photochemically inactive mono- and dinuclear-cyclometalated derivatives.^19−24^ Even so, functionalization of the phenyl rings of these derivatives with coordinating groups has also been conducted, with the aim of having a photoactive dangling azo moiety attached to a transition metal, while keeping its switching capabilities.^6,25−32^ The possible implication of these appended photoswitches in biologically relevant interactions has also been reported,^33,34^ and this is definitely an interesting area of research.^35^

In this respect, we have been recently involved in the attachment of azo derivatives to {Fe^II/III^(CN)_5_} units producing water-soluble complexes with a very clear switching and redox activity^36^ that can be potentially incorporated to mixed valence complexes. Interestingly, the azo derivatives utilized there undergo important mechanistic changes with respect to the spontaneous cis-to-trans reaction once coordinated. In these complexes, while their π–π* transition is only slightly modified, the corresponding nb−π* transition overlaps with a new solvatochromic MLCT band, which modifies their UV–vis spectra in an important way. As indicated above, few examples exist with several azo derivatives attached to the same metal center, and inclusion in 2D and 3D transition metal frameworks has been attained in some cases.^37−41^ From these examples, where more than one azo derivative are coordinated to the same metal center,^6,19^ the possible cooperative effect in the switching behavior of the diazene units has not been investigated, despite its potential interest.

With all this data at hand, we have decided to follow up our investigations by attaching several functionalized azo derivatives to transition metal centers in order to ascertain any changes that can be observed in their switching behavior. In the present study, we have started from the classical palladium and platinum {M(diphosphane-κ^2^*P*)}^2+^ building blocks (diphosphane-κ^2^*P* being 1,3-bis(diphenylphosphanyl)propane, dppp), or {M_2_(μ_2_-tetraphosphane-κ^2^*P*)}^4+^ (μ_2_-tetraphosphane-κ^2^*P* being 1,2,4,5-{tetrakis(diphenylphosphanyl)benzene}, tpbz) with the aim to study the possible cooperativity in the switching behavior of two or four linear coordinated diazene units at ca. 90°.

## Results and Discussion

### Azo Ligands

**Figure 1:**
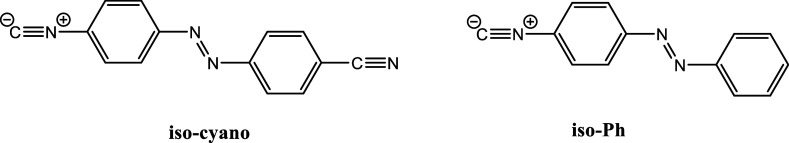
CN(C_6_H_4_)-N=N-(C_6_H_4_)CN (**iso-cyano**), and (4-isocyanophenyl)diazenylphenyl, CN(C_6_H_4_)-N=N-(C_6_H_5_) (**iso-Ph**) compounds utilized in this work.

Both 4-((4-isocyanophenyl)diazenyl)benzonitrile, CN(C_6_H_4_)-N=N-(C_6_H_4_)CN (**iso-cyano**), and (4-isocyanophenyl)diazenylphenyl, CN(C_6_H_4_)-N=N-(C_6_H_5_) (**iso-Ph**) compounds (Figure 1) have been prepared by optimization of the standard methods described in the literature; i.e. diazonium salt formation, azo coupling, amide formation, and dehydration of the latter using triphosgene.^26,42−44^

The ligands have been characterized by the standard methods, and the data agree with the already reported in the literature.^36,45^

The ^1^H NMR spectra of irradiated solutions at 365 nm of these molecules feature the set of signals assigned to the *cis* isomers, apart from the thermally dominant *trans* isomeric form, that are reverted to the original spectra by irradiation at 450 nm. Thus, all these compounds undergo the typical photochemical trans-to-cis isomerization process around the diazenyl unit.^3^ Although for the **iso-cyano** ligand, the spontaneous cis-to-trans thermal conversion had already been studied kinetically as a function of solvent, temperature and pressure,^36^ the study has now been completed using acetonitrile and dichloromethane, the solvents that are common to those used for the present study of the **iso-Ph** trans-to-cis thermal isomerization. Table 1 collects all the relevant kinetic and activation data obtained for these ligands.

**Table 1:** Kinetic (Interpolated at 313 K) and Activation Parameters for the Spontaneous cis-to-trans Isomerisation after Photoexcitation of the Ligands Utilised in this Work (Figure 1) as a Function of the Solvent Used

compound	solvent	^313^*k*_*cis*-to-*trans*_/s^–1^	Δ*H*^‡^/kJ mol^–1^	Δ*S*^‡^/J K^–1^ mol^–1^	Δ*V*^‡^/cm^3^ mol^–1^
**iso-cyano**	toluene^a^	5.4 × 10^–5^	95 ± 10	–26 ± 30	not determined
	dichloromethane	4.1 × 10^–5^	91 ± 2	–41 ± 5	not determined
	acetonitrile	2.4 × 10^–5^	89 ± 5	–52 ± 15	ca. 0 (63 °C)
	methanol^a^	7.3 × 10^–5^	97 ± 4	–17 ± 13	ca. 0 (48 °C)
**iso-Ph**	toluene	6.0 × 10^–5^	85 ± 5	–54 ± 17	4.4 ± 0.2 (63 °C)
	dichloromethane	1.7 × 10^–5^	90 ± 3	–52 ± 11	not determined
	acetonitrile	1.0 × 10^–5^	99 ± 6	–27 ± 18	4.2 ± 0.7 (63 °C)
	methanol	1.3 × 10^–5^	93 ± 2	–44 ± 7	not determined

a Indicates from ref (36).

From the data in Table 1, it is clear that the **iso-cyano** derivative shows a solvent-independent process with a practically null activation volume, a fact that has been repetitively associated with an inversional mechanism^36^ with high enthalpic requirements and no polar transition state (no ordering/contraction in polar solvents). Additional experiments with new solvents (dichloromethane and acetonitrile) do not introduce significant changes, as observed both in the value of Δ*V*^‡^ and in the lack of any trend in the values of the enthalpies and entropies of activation with the polarity of the solvents (Figure S1).^13,46^ Contrarily, from the same experiments with the **iso-Ph** derivative, results clearly indicate that the cis-to-trans spontaneous thermal conversion mechanism is distinct.^16^ In this case the activation volumes determined are nonzero, even when toluene solutions are utilized,^16^ indicative of a charge-separated rotational transition state.^47^ Furthermore, there is a clear trend in the values of Δ*H*^‡^ and Δ*S*^‡^ with the polarity of the solvent (Figure S1), also indicative of a charge involvement in the transition state of the process.

### [M(dppp)(azo)_2_](OTf)_2_ Complexes

**Figure 2:**
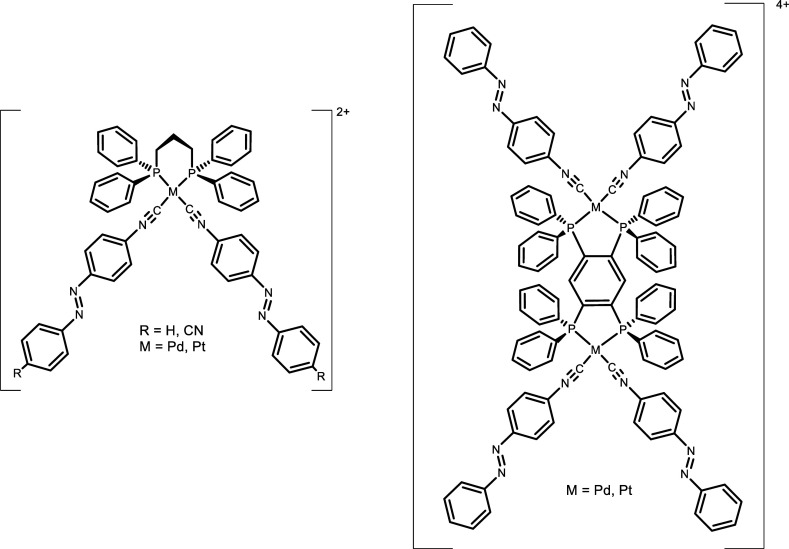
Schematic drawing of the [M(dppp)(azo)_2_]^2+^ and [{M_2_(tpbz)}(azo)_4_]^4+^ compounds.

The [M(dppp)(azo)_2_](OTf)_2_ compounds, with azo being **iso-cyano** and **iso-Ph** ligands (Figure 2, left), and M being Pd^II^ or Pt^II^ have been prepared in very good yields from the [M(dppp)(H_2_O)_2_](OTf)_2_ complexes by stoichiometric aqua by azo ligand substitution in dichloromethane solution, followed by partial evaporation of the solvent and addition of diethyl ether for precipitation, as indicated in the Experimental Section. The desired compounds crystallize easily, and the solids obtained were analyzed via ESI MS, IR and NMR spectroscopy. Mass spectra showed, in all cases, a signal corresponding to the double charged {[M(dppp)(azo)_2_]}^2+^ fragment and other intense peaks that result from decoordination of one azo ligand (Figures S2–S5). As expected, the IR spectra feature the shifted C≡N stretching signal of the {M-C≡N} blocks for all the systems (appearing at ca. 2225 cm^–1^ from the originally 2130 cm^–1^ in the free ligands). Furthermore, the band of the dangling nitrile unit in the [M(dppp)(**iso-cyano**)_2_](OTf)_2_ compounds is always observed at the position corresponding to the free ligand (ca. 2227 cm^–1^) as a shoulder of the isonitrile group stretching indicated above. ^1^H NMR is also clearly indicative of the nature of the complexes prepared with an evident upfield shift of the signals of the *ortho*-phenylisonitrile protons, from 7.70 ppm for the free ligands to ca. 7.30 ppm in the complexes. The ^31^P NMR spectra also agree with the purity of the compounds prepared showing a single resonance at ca. 0 ppm for all the palladium complexes and at ca. −16 ppm for the platinum analogues. The concomitant ^195^Pt satellites are also observed for the platinum complexes (*J*_P–Pt_ ≈ 2500 Hz). Figures S6–S13 show representative NMR spectra of the complexes prepared.

**Figure 3:**
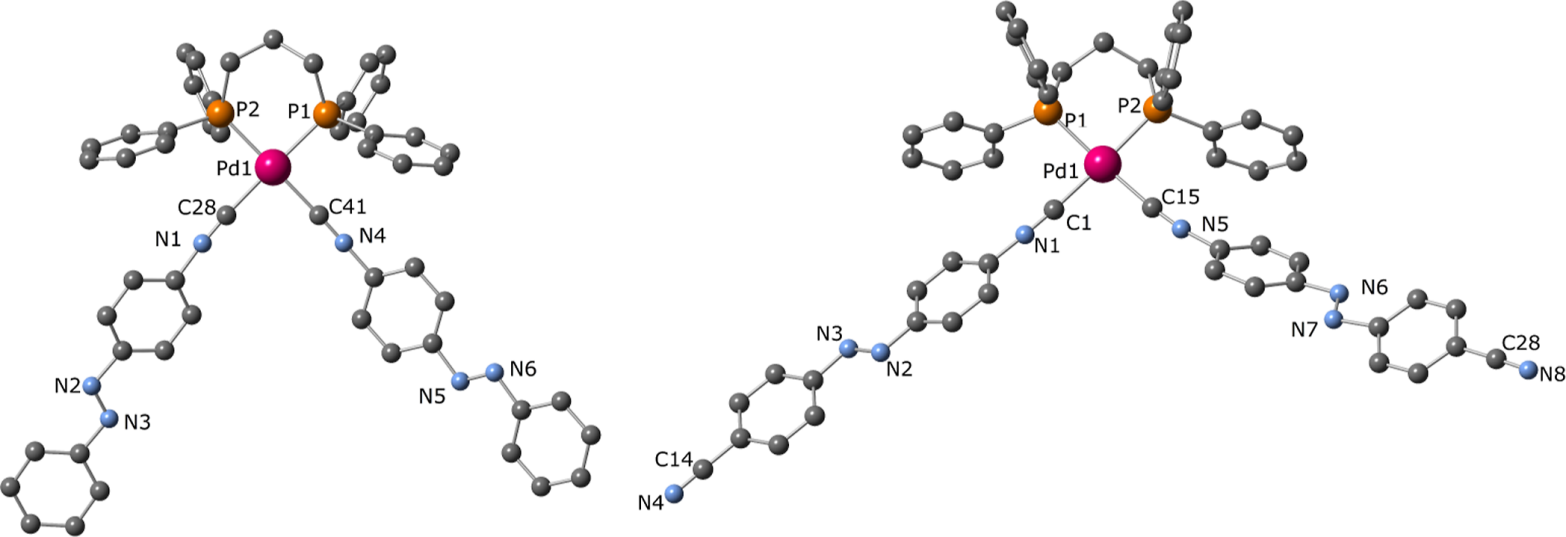
View of the XRD-determined [Pd(dppp)(**iso-Ph**)_2_]^2+^ (left) and [Pd(dppp)(**iso-cyano**)_2_]^2+^ (right) structural units (hydrogen atoms, solvent and counteranions not shown for clarity). Selected bond lengths (Å) and angles (°) for [Pd(dppp)(**iso-Ph**)_2_]^2+^: Pd(1)C(28) 2.042(4), Pd(1)C(41) 2.023(4), C(28)N(1) 1.119(5), C(41)N(4) 1.137(5), C(28)Pd(1)C(41) 88.25(18); for [Pd(dppp)(**iso-cyano**)_2_]^2+^: Pd(1)C(1) 2.002(8), Pd(1)C(15) 2.029(9), C(1)N(1) 1.152(11), C(15)N(5) 1.131(12), C(14)N(4) 1.127(12), C(28)N(8) 1.150(12), C(1)Pd(1)C(15) 92.8(3).

XRD studies on single crystals have been possible for the two palladium complexes [Pd(dppp)(**iso-Ph**)_2_](OTf)_2_ and [Pd(dppp)(**iso-cyano**)_2_](OTf)_2_ (Figure 3). The determined structural data are in the normal range found for the limited number of square planar palladium compounds containing a diphosphane and two isocyanido ligands.^48,49^ The two *trans*-azo ligands are in a fully “spread-out” distant fashion (the angles defined by the C–N=N–C planes being 141 and 161°, for the **iso-Ph** and **iso-cyano** compounds, respectively). These are very different from that determined for the related unique example of a d^8^ square-planar M^II^*bis*-azo complex (angle of 22° between the C–N=N–C planes) where the *trans*-azo ligands are not linear.^6^ This disposition may be associated with the existence of π–π stacking interactions that in the case of [Pd(dppp)(**iso-Ph**)_2_](OTf)_2_ are formed between Ph and PhNC rings of adjacent unit cells (Figure S14) and for the [Pd(dppp)(**iso-cyano**)_2_](OTf)_2_ result in pairs of Pd complexes within the same unit cell (Figure S15). Table S1 collects the crystal and structure refinement data for these structures.

**Figure 4:**
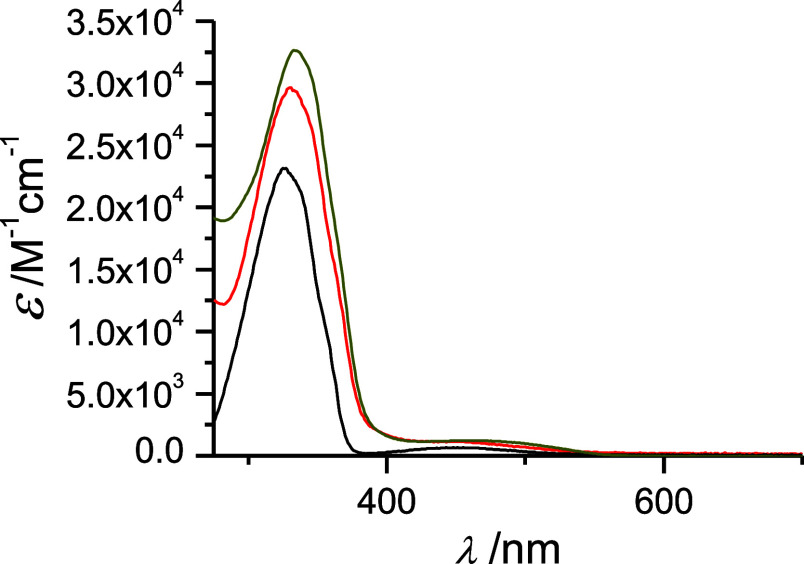
UV–vis spectra of the **iso-Ph** ligand (black) and its [Pd(dppp)(**iso-Ph**)_2_]^2+^ (red, divided by two) and [{Pd_2_(tpbz)}(**iso-Ph**)_4_]^4+^(olive, divided by four) derivatives in dichloromethane solution.

The UV–vis spectra of the complexes prepared show very interesting features as for their intensity with reference to the noncoordinated **iso-Ph** and **iso-cyano** molecules (Figure 4 and Table S2); the intensity of the π–π* and nb−π* bands of the complexes are greater than twice those of the free ligands. A clear involvement of the metal centers in the MO of the azo molecules can be claimed although no clear trend is observed between the Pd^II^ and Pt^II^ complexes. In this respect, the late transition nature of the Pt^II^ and Pd^II^ centers, together with the positive overall charge of the complexes produce a poor π-acidic character of the bond with the isocyanide fragment as also been suggested recently.^50^ These observations are very interesting in view of the opposite effect observed on coordination of these type of ligands to negatively charged {Fe^II^(CN)_5_}^3–^ units.^36^ In that case, a clear decrease in the intensity of the π–π* band was observed, accompanied by a dramatic intensity increase of the nb−π* signal, the latter due to the appearance of an overlapping MLCT band from the negatively charged {Fe^II^(CN)_5_}^3–^ unit. In the present compounds no MLCT bands are observed, which can be associated with the positive charge in the {M(phosphane)}^2+^ blocks.

### [{M_2_(tpbz)}(azo)_4_](OTf)_4_ Complexes

The [{M_2_(tpbz)}(azo)_4_](OTf)_4_ compounds with the **iso-Ph** azo ligand (Figure 2 right), and M being Pd^II^ or Pt^II^ have been prepared, as those of the dppp phosphane, from the [{M_2_(tpbz)}(CH_3_CN)_4_](OTf)_4_ complexes by stoichiometric acetonitrile by azo ligand substitution in dichloromethane solution, as indicated in the Experimental Section. After precipitation with diethyl ether the solids obtained were analyzed via ESI MS, IR and NMR spectroscopy. Mass spectra showed, in all cases, a signal corresponding to the quadruple charged {[{M_2_(tpbz)}(**iso-Ph**)_4_]}^4+^ fragment and other peaks that result from the subsequent loses of triflate anions from the parent [{M_2_(tpbz)}(azo)_4_](OTf)_4_ complex (Figures S16 and S17). ^1^H NMR is indicative of the nature of the complexes prepared with an upfield shift of the signals of the *ortho*-phenylisonitrile protons of ca. 0.40 ppm from the free ligands, analogously to the observed for the dppp systems. The ^31^P NMR spectra also agree with the purity of the compounds prepared showing a single resonance at 53.4 ppm for the palladium complex and at 35.0 ppm for the platinum analog (with the concomitant ^195^Pt satellites with *J*_P–Pt_ = 2660 Hz). The appearance of the ^31^P NMR signal at lower fields than that for the [M(dppp)(azo)_2_](OTf)_2_ derivatives indicates a larger electron density on the metal center due to the tetraphosphane ligand (i.e., greater basicity); Figures S18 and S19 show representative NMR spectra of the complexes prepared. The IR spectra feature the shifted C≡N stretching signal of the {M-C≡N} blocks appearing at ca. 2200 cm^–1^ from originally 2130 cm^–1^ in the free ligand. This value is ca. 25 cm^–1^ lower than that observed for the [M(dppp)(azo)_2_](OTf)_2_ derivatives, indicating again a higher electron density on the metal center and a greater back-donation to the π-antibonding orbitals of the C≡N units on the azo ligand.

As for the UV–vis spectra of these tpbz complexes prepared (Table S2 and Figure 4), the same discussion above about the intensity features of the π–π* band, with respect to the noncoordinated **iso-Ph** molecules, than that for the [M(dppp)(azo)_2_](OTf)_2_ analogues applies. That is, the intensity of the band is higher than the expected from the addition of the contribution from each of the azo derivative ligands.

### Photoswitching Activity of the Coordinated Azo Ligands

**Figure 5:**
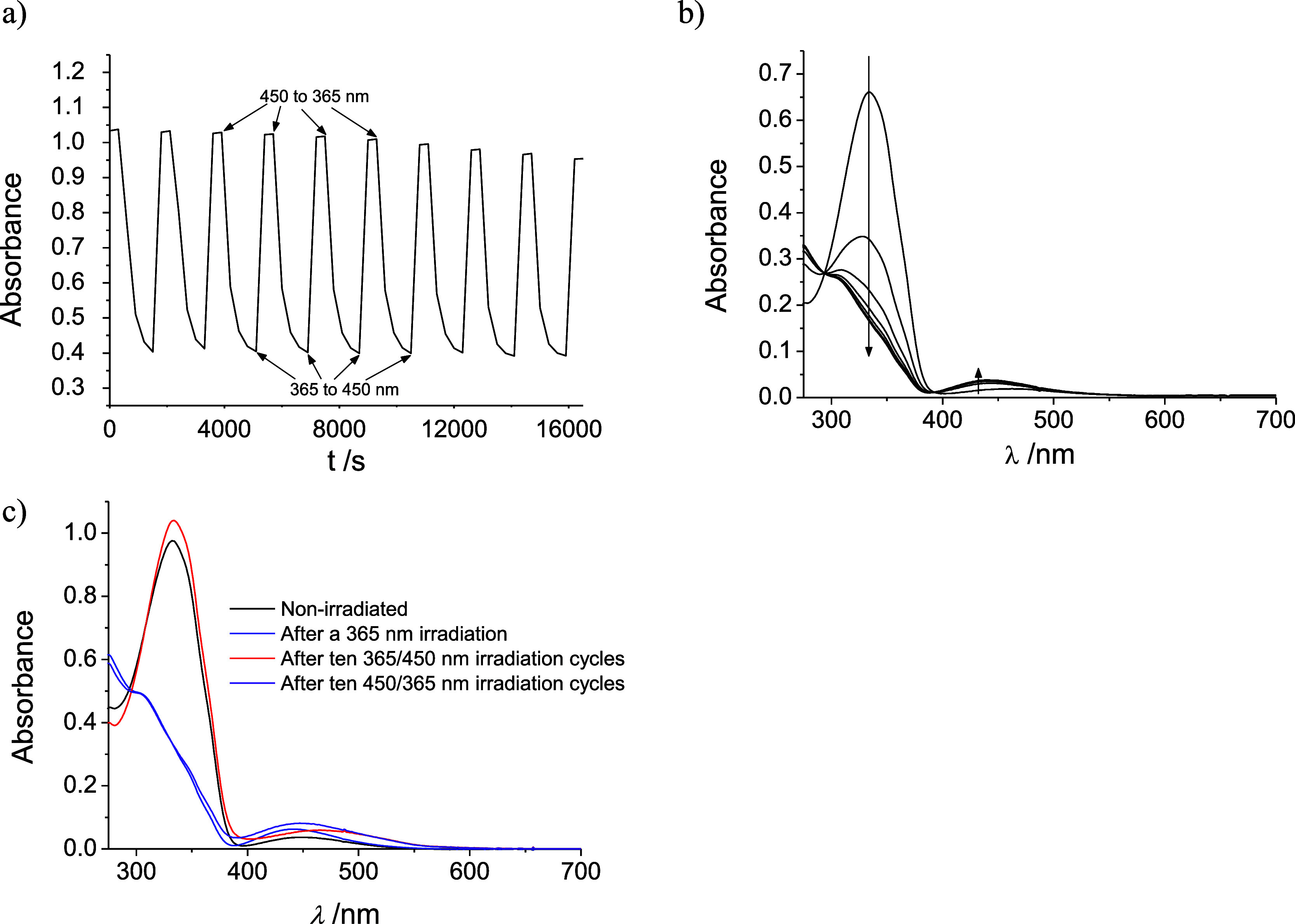
(a) Changes in the intensity of the UV–vis signal at 340 nm of a dichloromethane solution of [Pt(dppp)(**iso-Ph**)]^2+^ on consecutive irradiation cycles at 365 and 450 nm. (b) Full UV–vis time-resolved spectral changes of the same solution on illumination at 365 nm. (c) Initial and final spectrum (after 10 illumination cycles) of the same solution.

As the ligands have shown to possess the classical photoswitching capability of the azobenzene derivatives (see above), and given the fact that such a capability is usually transferred to coordination complexes, we have studied the photoswitching of the complexes alternating 365 and 450 nm illumination. Figure 5a shows the changes in intensity of the UV–vis spectrum measured at 340 nm for several illumination cycles for one of the [M(dppp)(azo)_2_]^2+^ complexes studied in dichloromethane solution; Figure 5b features the full UV–vis time-resolved spectral changes on illumination at 365 nm. Clearly, the photoswitching process is quite robust as only a ca. 4–8% of the signal is lost after several cycles. As seen in Figure 5c, for dichloromethane solutions, this loss is reflected by a bathochromic shift in the maximum of the nb–π* signal; this has been associated with the generation of HCl in the irradiated dichloromethane and consequent protonation of the azo ligands.^10,51^ Although a change of solvent should avoid this problem, the use of acetonitrile (Figure S20), or methanol for the solution of the complexes does not lead to an increase of the robustness of the systems. In both solvents the [M(dppp)(azo)_2_]^2+^ complexes show some decomposition on standing, even in the dark, especially at low concentrations. While in acetonitrile solutions the compounds present significant changes in the ^1^H and ^31^P NMR spectra after 4–5 days at room temperature, for methanol solutions these changes are evident after 1 day. Most probably, the observed decomposition involves substitution reactions on these fairly labile systems with participation of solvent molecules (or their ubiquitous water content). In this respect, the robustness of the [{M_2_(tpbz)}(**iso-Ph**)_4_]^4+^ analogues proved to be much lower even in dichloromethane solution (see Figure S21); the larger basicity of the tetraphosphane ligand (see above, ^31^P NMR chemical shifts), creating a higher *trans*-influence on the coordinated azo derivatives, should be held responsible for this fact. Therefore, the studies related to the thermal back cis-to-trans process (see below) have been limited to ambient pressure and using fresh solutions for each run; Figure S22 shows the feasibility of such studies on freshly prepared solutions when a single switching process was studied.

Interestingly, the spectral results indicated in Figure 5b show neat isosbestic points at ca. 390 and 290 nm (a fact that applies for all the systems studied), which indicates that there is a lack of cooperativity between the two azo ligands attached to the metal center; the behavior can be considered statistical. That is, no two photoswitching consecutive processes are observed, in clear difference from the reactivity of other published *bis*-azo complexes.^6^

**Figure 6:**
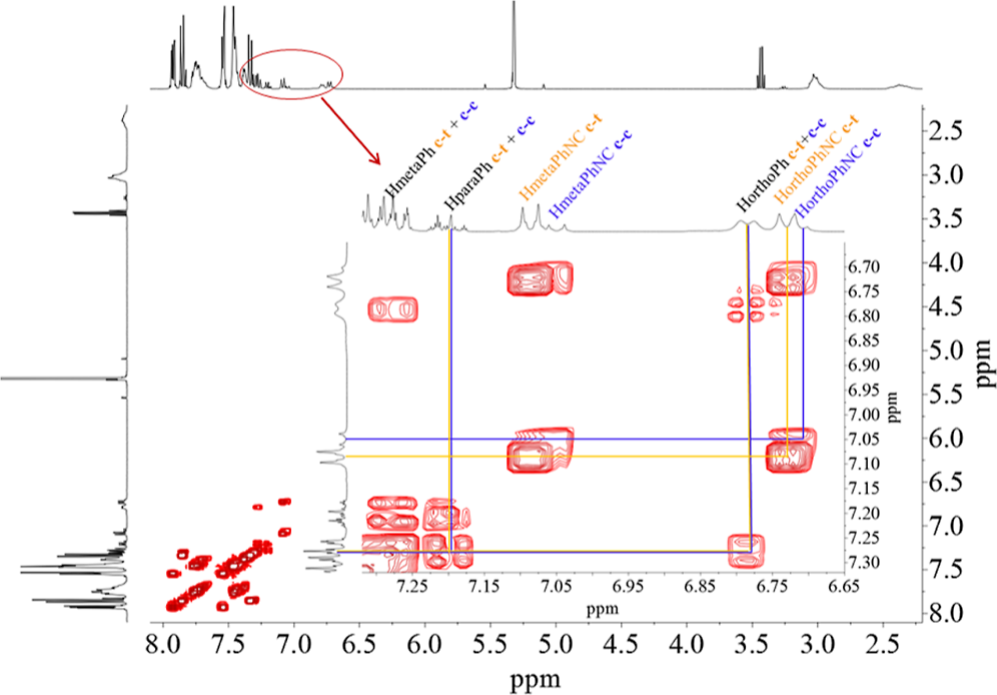
COSY ^1^H–^1^H NMR spectrum of a solution of [Pd(dppp)(*trans*-**iso-Ph**)_2_]^2+^ in CD_2_Cl_2_ and expansion indicating the proton signals of the ligand in the less predominant *cis*–*trans* (c–t) and *cis*–*cis* (c–c) complexes.

**Figure 7:**
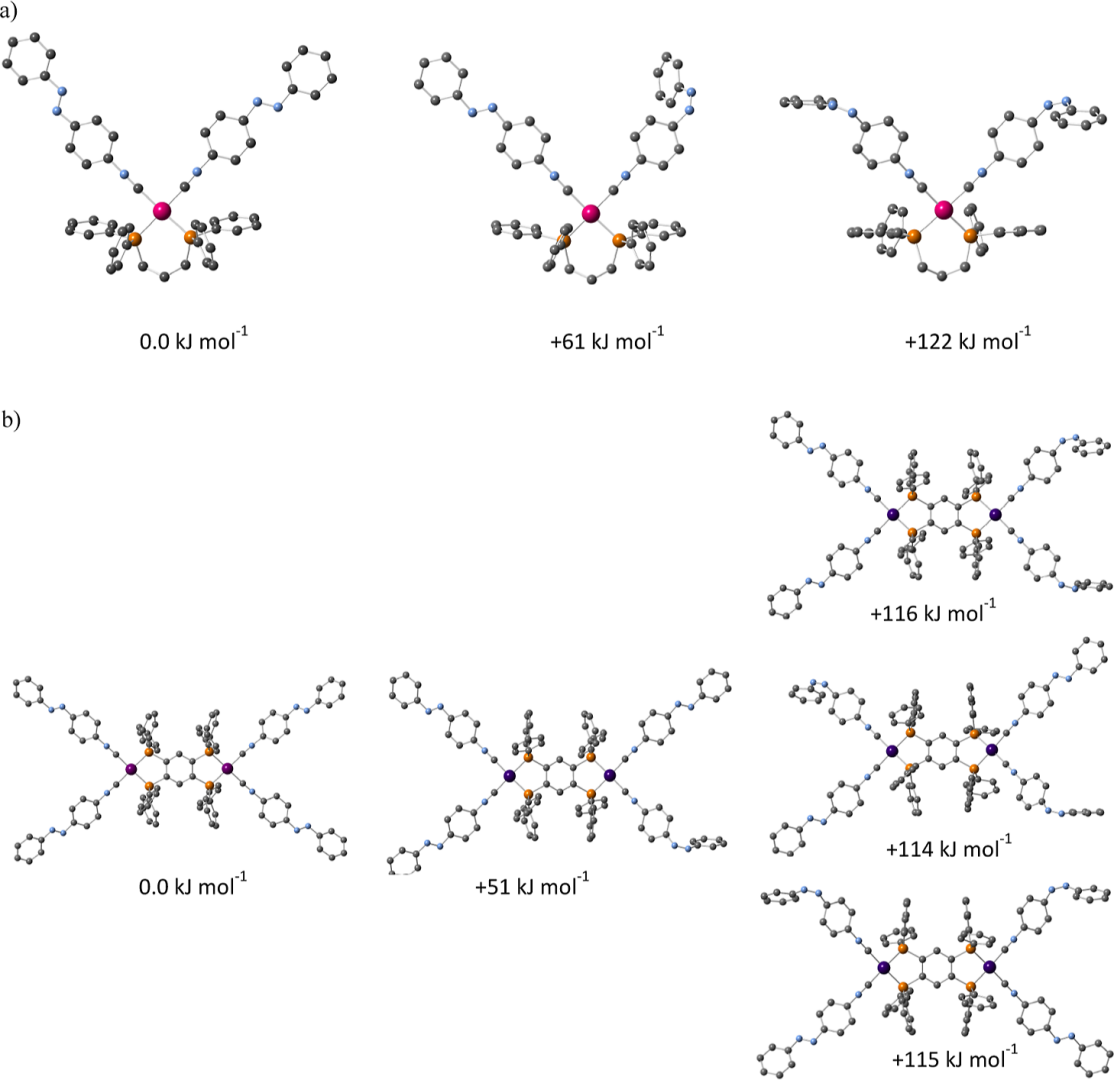
Calculated energy differences between the structures of the *trans-***iso-Ph** and *cis-***iso-Ph** units in the (a) [Pd(dppp)(**iso-Ph**)_2_]^2+^ and (b) [{Pd_2_(tpbz)}(**iso-Ph**)_4_]^4+^ complexes.

The spread-out disposition of the two **iso-cyano** and **iso-Ph** ligands in the coordination sphere of the Pd^II^ and Pt^II^ centers, as observed in the structures featured in Figure 3, should be held responsible of such a fact. Alternatively, the systems could have a single photoswitching azo unit that prevents follow-up consecutive isomerisations;^31^ a comprehensive interpretation of the COSY ^1^H NMR spectrum of 365 nm illuminated samples of some of the complexes studied was thus conducted (Figures S23 and S24). As seen in Figure 6, the signals associated with the thermodynamically stable [Pd(dppp)(*trans*-**iso-Ph**)_2_]^2+^ species are dominant in the spectrum, but the signals corresponding to the *trans* and *cis* forms of the ligands of the [Pd(dppp)(*trans*-**iso-Ph**)(*cis*-**iso-Ph**)]^2+^ species are also univocally observed, as are those for the *cis* ligands in the [Pd(dppp)(*cis*-**iso-Ph**)_2_]^2+^ species [relative composition in the photostationary state has been calculated approximately as 70% (*trans*–*trans*), 25% (*trans*–*cis*) and 5% (*cis*–*cis*)]. Parallel ^31^P NMR monitoring of the sample (Figure S25) also shows the presence of two singlets corresponding to the major [Pd(dppp)(*trans*-**iso-Ph**)_2_]^2+^ and minor [Pd(dppp)(*cis*-**iso-Ph**)_2_]^2+^ symmetrical complexes, *plus* other two intermediate intensity resonances associated with the nonequivalent phosphorus atoms in the [Pd(dppp)(*trans*-**iso-Ph**)(*cis*-**iso-Ph**)]^2+^ species. The expected doublets are not observed for the latter, as the difference in chemical shift is too small; in fact, in acetonitrile solution all these signals merge as a broad singlet. For the other [M(dppp)(azo)_2_]^2+^ complexes studied, similar trends were observed, serving as an indication of the presence of more than a single species. For the [{M_2_(tpbz)}(**iso-Ph**)_4_]^4+^ analogues, the study indicated the appearance of a broad signal that corresponds to a mixture of the possible photoisomeric forms formed (Figure S26). That is, although a single form of [{M_2_(tpbz)}(*cis-***iso-Ph**)(*trans-***iso-Ph**)_3_]^4+^ and [{M_2_(tpbz)}(*cis-***iso-Ph**)_3_(*trans-***iso-Ph**)]^4+^ occurs, three forms are possible for the [{M_2_(tpbz)}(*cis-***iso-Ph**)_2_(*trans-***iso-Ph**)_2_]^4+^ double photoswitched species (Figure 7b).

So, the only way to come to terms with the mentioned uniform switching behavior, despite the existence of multiple species in solution on illumination at 365 nm (as indicated in Figure 6 for [Pd(dppp)(*trans*-**iso-Ph**)_2_]^2+^, [Pd(dppp)(*trans*-**iso-Ph**)(*cis*-**iso-Ph**)]^2+^ and [Pd(dppp)(*cis*-**iso-Ph**)_2_]^2+^), is effectively to accept a simple statistical behavior of the photoisomerisation process. That is, the trans-to-cis photoisomerisation reaction of the azo units in the complexes is governed by the process occurring *individually* on each ligand, producing very simple UV–vis changes. In order to ascertain the feasibility of this assumption, density functional theory (DFT) calculations have been conducted on the possible thermodynamic cooperativity of these processes. As an example, for the [Pd(dppp)(**iso-Ph**)_2_]^2+^ and [{Pd_2_(tpbz)}(**iso-Ph**)_4_]^4+^ complexes (Figure 7) the possible switching processes have been found to involve the same energy difference within the calculation errors (ca. 50–60 kJ mol^–1^), which agrees with a statistical photoisomerisation. Furthermore, the energy difference between the *trans-***iso-Ph** and *cis-***iso-Ph** noncoordinated ligands has been calculated also to be 58 kJ mol^–1^, equivalent to that featured in Figure 7 for the ligands in the complexes, thus indicating the effective independence between the switching processes of each ligand.**

**Figure 8:**
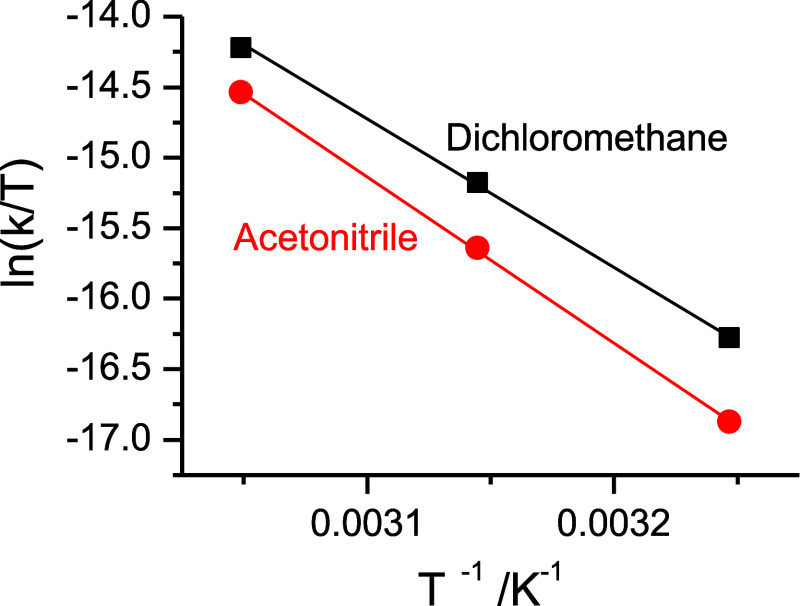
Eyring plot for the derivation of the activation parameters for the cis-to-trans isomerization process occurring on the system [Pd(dppp)(**iso-cyano**)_2_]^2+^ in different solvents.

Given the positive synergy found on the intensity of the UV–vis spectra and this lack of cooperativity in the photoisomerisation reaction, the study of the spontaneous back cis-to-trans reaction becomes even more appealing. Following our interest,^16,17^ in these spontaneous back switching processes, the reactions were studied at different temperatures, pressures and solvent conditions in order to ascertain possible changes in the thermal operating mechanism between the free and coordinated ligands. The processes were studied after illumination of the UV–vis cells at 365 nm until the photostationary state is attained (see Figure 5a,b). As found for the photoisomerisation processes, the spontaneous cis-to-trans recovery was observed occurring in a single step (Figure S27), indicating that effectively the two azo ligands in the complexes behave independent with respect to the full trans-to-cis-to-trans processes;^52−54^ in all cases, nevertheless, a final drift on the recovery of intensity of the π–π* band is observed. As for the photochemical processes indicated above, in acetonitrile or methanol solution this fact has been associated with the partial substitution of the ligands (on long-standing or relative high temperatures), and in dichloromethane solution to the protonation of the azo derivatives by photochemical generation of HCl. It is also interesting to indicate that, as illumination at 450 nm produces a fast photochemical recovery of the intensity of the π–π* band (see Figure 5a), diode array spectrophotometers produce rather unreliable results with respect to the monitoring of the spontaneous cis-to-trans process, thus obliging the use of scanning instruments with short illumination pulses (see Experimental Section part). Table 2 collects all the relevant kinetic and activation data for the systems studied; some examples of the Eyring plots obtained for the systems studied are shown in Figure 8.

**Table 2:**
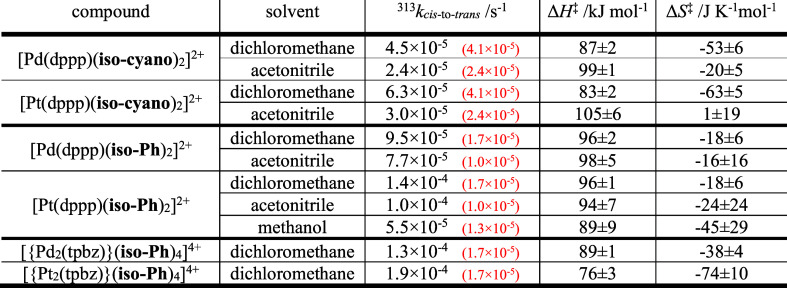
Kinetic (Interpolated at 313 K) and Activation Parameters for the Spontaneous Thermal cis-to-trans Isomerisation after Photoexcitation of the Complexes Prepared in this Work as a Function of the Solvent Used^a^

a Values in brackets in red correspond to the free ligand, as featured in Table 1.

As observed in Table 2, for both the **iso-Ph** and the **iso-cyano** complexes of the dppp family only slight differences in the *k*_*cis*-to-*trans*_ rate constants are observed on going from the palladium to the platinum complexes (with no meaningful changes of the activation parameter values). Clearly, the spontaneous cis-to-trans process seems to be occurring from a simple ligand-based perspective. Furthermore, for the **iso-cyano** ligand, only small changes in the value of the *k*_*cis*-to-*trans*_ spontaneous rate constant occur on its coordination to the metal centers, but for the **iso-Ph** an increase of ca. 1 order of magnitude for both the dppp and tpbz family of compounds is observed. Interestingly, as seen in Figure 8, a definite decrease in the value of the *k*_*cis*-to-*trans*_ rate constant for these species is evident on increasing the polarity index of the solvent (3.1 for dichloromethane, 5.8 for acetonitrile),^55^ as well as their protic/water content capability (acetonitrile, methanol), indicating the actuation of a polar transition state mechanism.

**Figure 9:**
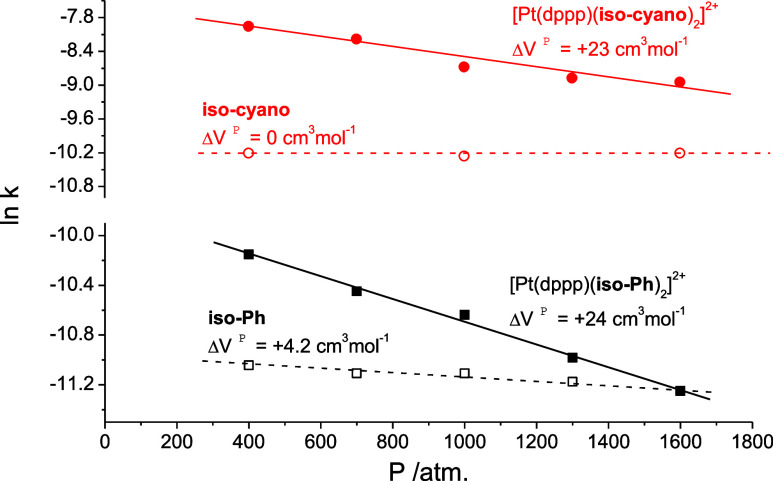
ln *k* versus *P* plots obtained for the spontaneous cis-to-trans isomerization of the **iso-Ph** and **iso-cyano** units either as free ligands or in their platinum complexes in acetonitrile solution (*T* = 63 °C for the **iso-Ph** and **iso-cyano** ligands, 60 °C for the [Pt(dppp)(**iso-Ph**)_2_]^2+^ complex, and 52 °C for the [Pt(dppp)(**iso-cyano**)_2_]^2+^ complex).

Comparison of the values of the pressure dependence of the rate constants for the **iso-Ph** and **iso-cyano** free and coordinated ligands within the dppp family of complexes is definitively more revealing, as shown in Figure 9. For both ligands, a clear shift to more positive values of Δ*V*^‡^ is observed (from 0 to +23 cm^3^ mol^–1^ for the **iso-cyano** units and from +4.2 to +24 cm^3^ mol^–1^ for the **iso-Ph** analogues) on coordination to the Pt^II^ centers.

**Scheme 2:**
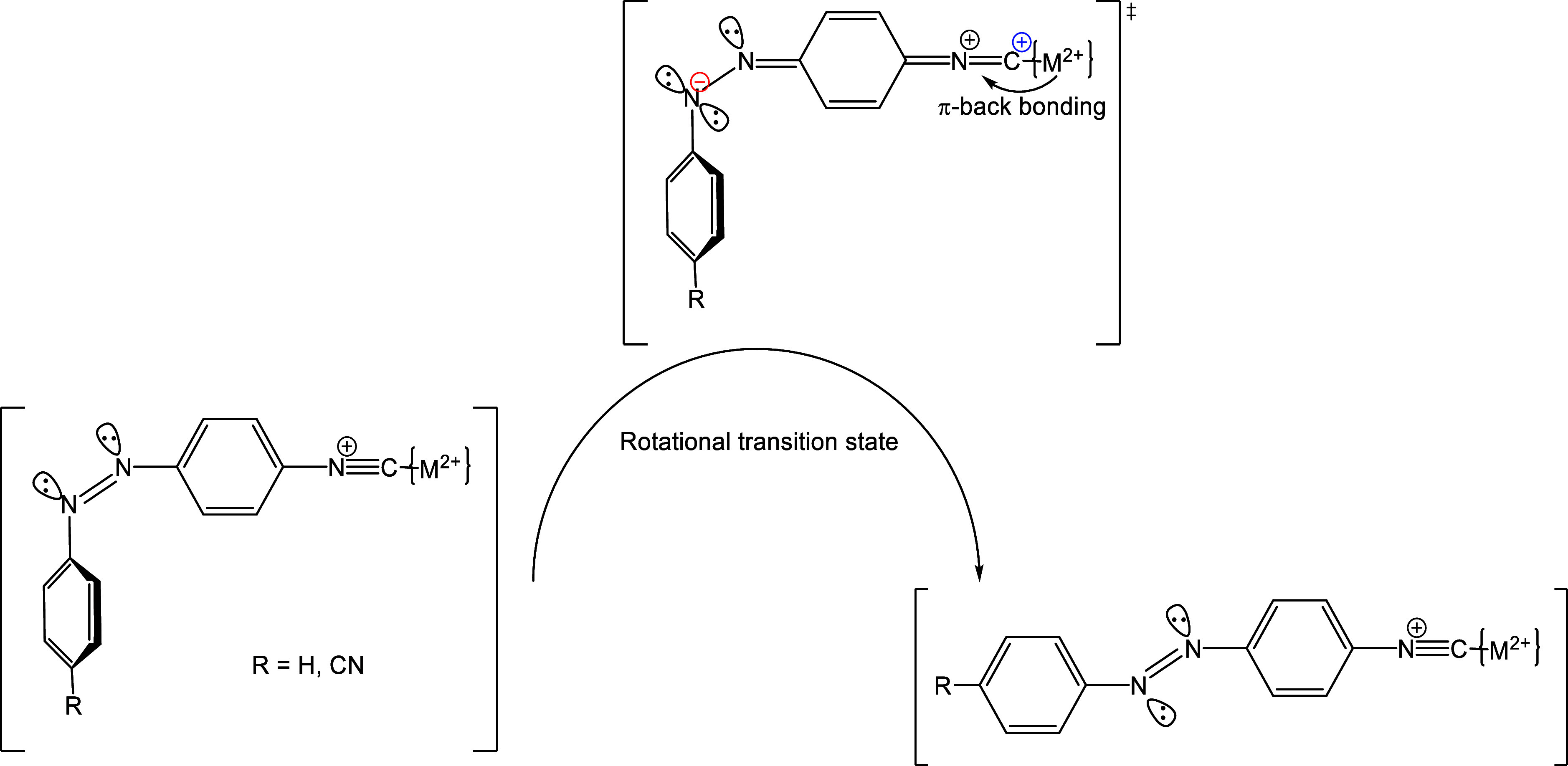


It seems clear that for the **iso-Ph** molecule the spontaneous polar mechanism for the cis-to-trans isomerization is maintained on coordination with only minor effects on Pd^II^ or Pt^II^ attachment to the isonitrile unit. For the analogous **iso-cyano** derivative, nevertheless, important and definite changes take place as “desymmetrisation” of the diazenyl unit occurs on coordination to a charged moiety to the isocyanido terminal group of the switching azo molecule. This pressure-dependence result is in full agreement with that observed when iron pentacyanido moieties have been attached to one or two of the terminal groups of the analogous **iso–iso** ligands.^36^ Clearly, the attachment of a coordination complex, either positively or negatively charged, produces a changeover of the thermal switching isomerization of the diazenyl units by changing the symmetry. In the present case the electrons of the filled d_*xy*,*xz*,*yz*_ orbitals of the palladium and platinum centers can contribute to the stabilization of the positive charge generated on the carbon donor in the transition state, thus favoring the concomitant charge separation^50^ and the rotational isomerization mechanism in both complexes (Scheme 2).

## Conclusions

The coordination of the azo derivatives studied with isonitrile terminal groups to d^8^ positively charged square-planar metal centers, with a single or double {M^II^(diphosphane-κ^2^*P*)}^2+^ scaffold has been attained. The systems have been characterized by the standard techniques and show a spread-out geometry around the metal center, as proved by the XRD structures determined for some of the species.

The photoswitching trans-to-cis capability of the coordinated azo units attached has also been determined and found to behave independently. A thorough NMR spectral study of the **iso-Ph** mononuclear systems has allowed for the detection and identification of the three expected photoisomers. DFT calculations also agree with the fact that all the photoswitching reactions within the complexes have equivalent energy differences. The values are also found equivalent to those for the free ligands, thus corroborating the independent nature of the reactions for these complexes.

The spontaneous back cis-to-trans isomerization reaction of the photoswitched azo units in the complexes has also been studied; in all cases the thermal processes have also been found occurring independently in each of the diazenyl units. Interestingly, for the ligands studied, a clear increase in the polarity of the transition state (rotational mechanism operating) occurs on coordination to the metal center. This fact takes place both starting from an inversional mechanism, for the **iso-cyano** ligand, and from an already rotational mechanism, for the **iso-Ph** ligand. The determination of the volumes of activation for these systems has been proved, again, crucial for determining the differences. The electronic nature of the metal centers and their π-donating capability to the isonitrile groups in the transition state are held responsible for such increase.

## Experimental Section

### Physical Methods

^1^H and ^31^P{^1^H} NMR spectra were recorded on a Bruker-400 or 500 spectrophotometers at 25 °C at the CCiT of the Universitat de Barcelona; infrared spectra were recorded on an FT-IR IS5 Nicolet Thermo scientific spectrophotometer; ESI mass spectra were recorded on a LC/MSD TOF Agilent Technologies 61969A or LTQ Orbitrap Velos SN03136B instruments in acetonitrile solutions using as a eluent a solution of CH_3_CN with formic acid 1% at the CCiT of the Universitat de Barcelona. Photochemical excitation of the azo derivatives was carried out using an ASAHI MAX-303 light source equipped with the desired filters and focused on a selected cell of a multicell support of an Agilent HP8453 instrument that was also utilized for monitoring the advance of the photoisomerisation process.

The kinetic profiles for the reactions at atmospheric pressure were followed by UV–vis spectroscopy in the 900–300 nm range on a Cary50 instrument equipped with thermostated multicell transport. The experiments were carried out in thigh-sealed quartz cuvettes (to avoid solvent evaporation at high temperatures) with a path length of 1 cm. For runs at elevated pressure, the previously described high-pressure setup^16,56^ was used connected to the same instrument. The full operative system and software^57,58^ used for the determination of the first order rate constants involved has already been described.^12,17,59^ All postrun fittings were carried out by the standard available commercial programs. In all instances, the rate constants were the average of two or three replicates with an error between the 10 and 15%. Table S3 collects all the first-order rate constants obtained for all the systems studied as a function of solvent, temperature and pressure.

The characterization of the products with the ligands in the *cis* form was performed irradiating the NMR tubs at 365 nm and registering the ^1^H, ^31^P{^1^H} and ^1^H–^1^H COSY at different times. The cis-to-trans reverse process was carried out either thermally (heating the tube at 40–45 °C) or photochemically by irradiating de tube at 450 nm.

### X-ray Structure Determination

XRD measurements were conducted on a D8 Venture system equipped with a multilayer monochromator and a Mo microfocus (λ = 0.71073 Å). The frames were integrated with the Bruker SAINT software package using a narrow-frame algorithm and the structure was solved and refined using the Bruker SHELXTL Software Package, using the space group *P*1̅, with *Z* = 2 for the formula unit, C_55_H_44_F_6_N_6_O_6_P_2_PdS_2_. Table S1 collect all the relevant data and the CCDC deposition numbers.

### Computational Details

All the DFT calculations have been carried out using the Gaussian09 (rev. D.01)^60^ software. The geometry optimization of all the species in the gas phase has been carried out with the BP86^61^ density functional. The 6-31G* basis set^62−64^ has been employed to describe the H, C, N and P atoms while the SDD basis set,^65−68^ along with the corresponding electron core potentials, has been used to describe the Pd and Pt atoms. Ultrafine integration grids have been used in all cases to ensure a satisfactory convergence. Vibrational analyses have been performed for all the computed structures to ensure the nature of the stationary points, which have zero imaginary frequencies. TD-DFT calculations have been performed with the time-dependent DFT method as implemented in Gaussian09.^69−75^ These calculations employ the same computational settings used in the geometry optimization process and include ten vertical singlet excitations. Table S4 collects computed DFT Cartesian coordinates and absolute Gibbs energies of the palladium and platinum complexes.

### Materials and Compounds

All preparations were carried out in a nitrogen atmosphere using standard Schlenk techniques. Chemicals used in the preparation procedures were analytical grade commercially available and were utilized as received. Solvents were dried by standard methods or, alternatively, obtained from a solvent purification system Puresolve (Innovative Technologies) and kept in a nitrogen atmosphere.

The starting materials, 1,1-bis(diphenylphosphanyl)propane (dppp) and silver trifluoromethanesulfonate (AgOTf), were commercially available, phosphane 1,2,4,5-tetrakis(diphenylphosphanyl)benzene was prepared according to the literature.^76^ Compounds [M(dppp)(H_2_O)_2_](OTf)_2_ and [{M_2_(tpbz)}(CH_3_CN)_4_](OTf)_4_ (M = Pd, Pt),^77,78^ as well as the azo derivatives (CN(C_6_H_4_)-N=N-(C_6_H_4_)CN (**iso-cyano**)) and (CN(C_6_H_4_)–N=N-(C_6_H_5_) (**iso-Ph**)) were prepared according to the methods reported in the literature.^36,43,79^

#### [Pd(dppp)(**iso-cyano**)_2_](OTf)_2_

In a purged Schlenk tube, 20 mg (0.024 mmol, 1 equiv) of [Pd(dppp)(H_2_O)_2_](OTf)_2_ were dissolved in 20 mL of CH_2_Cl_2_, resulting in a pale yellow solution. After the addition of 11 mg (0.048 mmol, 2 equiv) of **iso-cyano** the solution was protected from light and stirred for 20 min. After concentration to 5 mL, the product precipitated on addition of Et_2_O as an orange solid. The solid was filtered, washed with Et_2_O, and dried under vacuum. The yield was 74% (23 mg, 0.018 mmol).

### Before Irradiation (***trans*–*trans***)

^1^H NMR (500 MHz, CD_3_CN): 8.01 (pd, J(H–H) = 8.8 Hz, 4H, HmetaPhCN), 7.97–7.94 (m, 8H, HmetaPhNC + HorthoPhCN), 7.68–7.64 (m, 8H, Ph(dppp)), 7.58–7.55 (m, 4H, Ph(dppp)), 7.51–7.48 (m, 8H, Ph(dppp)), 7.29 (pd, J(H–H) = 8.4 Hz, 4H, HorthoPhNC), 2.93 (m, 4H, CH_2_–P(dppp)), 2.33 (m, 2H, C–CH_2_–C(dppp)).

^1^H NMR (400 MHz, CD_2_Cl_2_): 8.00 (pd, J(H–H) = 8.8 Hz, 4H, HorthoPhCN), 7.89 (pd, J(H–H) = 8.9 Hz, 4H, HmetaPhNC), 7.84 (pd, J(H–H) = 8.8 Hz, 4H, HmetaPhCN), 7.78–7.73 (m, 8H, Ph(dppp)), 7.46–7.42 (m, 12H, Ph(dppp)), 7.38 (pd, J(H–H) = 8.8 Hz, 4H, HorthoPhNC), 3.03 (m, 4H, CH_2_–P(dppp)), 2.37 (m, 2H, C–CH_2_–C(dppp)).

^31^P{^1^H} NMR (202 MHz, CD_3_CN): 0.9 s.

^31^P{^1^H} NMR (162 MHz, CD_2_Cl_2_): 1.0 s.

IR (cm^–1^): 2224 br (C–C≡N + M-C≡N), 1487 (dppp), 1438 (dppp), 1248 (OTf), 1147 (OTf), 1101 (dppp), 753 (dppp).

MS (ESI+) *m*/*z*: {[Pd(dppp)(CN(C_6_H_4_)-N=N-(C_6_H_4_)CN)_2_)]}^2+^ 491.11 (calc: 491.10); {[Pd(dppp)(CN)(CH_3_CN)]}^+^, 585.09 (calc: 585.09); {[Pd(dppp)(CN)(CN(C_6_H_4_)–N=N-(C_6_H_4_)CN)]}^+^, 776.14 (calc: 776.13).

UV–vis [λ_max_, nm (ε, M^–1^ cm^–1^)]: (CH_3_CN), 329 (79,600), 458 (1770); (CH_2_Cl_2_), 332 (72,900), 470 (1200).

### After Irradiation

^1^H NMR (500 MHz, CD_3_CN) (***cis*–*trans***): 7.68–7.64 (superimposed with phosphane signals, HmetaPhCN_***cis***_), 6.99 (pd, J(H–H) = 8.5 Hz, HmetaPhNC_***cis***_), 6.94 (pd, J(H–H) = 8.6 Hz, HorthoPhCN_***cis***_), 6.86 (pd, J(H–H) = 8.8 Hz, HorthoPhNC_***cis***_). All the resonances of the ligand in ***trans*** are superimposed with those corresponding to the analogous ***trans–trans*** photoisomer.

^1^H NMR (400 MHz, CD_2_Cl_2_) (***cis*–*trans***): 8.00 (superimposed with signals of ***trans*–*trans***, HorthoPhCN_***trans***_), 7.89 (superimposed with signals of ***trans*–*trans***, HmetaPhNC_***trans***_), 7.84 (superimposed with signals of ***trans*–*trans***, HmetaPhCN_***trans***_), 7.78–7.71 (m, superimposed with signals of ***trans*–*trans***, Ph(dppp)), 7.57 (pd, J(H–H) = 8.7 Hz, HorthoPhNC_***cis***_), 7.46–7.42 (m, superimposed with signals of ***trans*–*trans***, Ph(dppp)), 7.35 (pd, J(H–H) = 8.9 Hz, HorthoPhNC_***trans***_), 7.17 (pd, J(H–H) = 8.8 Hz, HmetaPhNC_***cis***_), 6.84 (pd, J(H–H) = 8.8 Hz, HmetaPhCN_***cis***_), 6.75 (pd, J(H–H) = 8.8 Hz, HorthoPhNC_***cis***_), 3.03 (m, superimposed with signals of ***trans*–*trans***, CH_2_–P(dppp)), 2.37 (m, superimposed with signals of ***trans*–*trans***, C–CH_2_–C(dppp)).

^31^P{^1^H} NMR (162 MHz, CD_2_Cl_2_): 1.03 s, 1.06 s (***cis*–*trans***).

#### [Pt(dppp)(**iso-cyano**)_2_](OTf)_2_

The procedure used was analogue to that described for [Pd(dppp)(**iso-cyano**)_2_](OTf)_2_. Twenty-five mg (0.027 mmol, 1 equiv) of [Pt(dppp)(H_2_O)_2_](OTf)_2_ were reacted with 12 mg (0.053 mmol, 2 equiv) of **iso-cyano** to give an 80% yield of a red-orange solid (30 mg, 0.022 mmol).

### Before Irradiation (***trans*–*trans***)

^1^H NMR (400 MHz, CD_3_CN): 8.03 (pd, J(H–H) = 8.9 Hz, 4H, HmetaPhCN), 7.97–7.95 (m, 8H, HmetaPhNC + HorthoPhCN), 7.70–7.65 (m, 8H, Ph(dppp)), 7.69–7.51 (m, 12H, Ph(dppp)), 7.28 (d, J(H–H) = 8.6 Hz, 4H, HorthoPhNC), 3.06 (m, 4H, CH_2_–P(dppp)), 2.36 (m, 2H, C–CH_2_–C(dppp)) ppm.

^1^H NMR (400 MHz, CD_2_Cl_2_): 8.00 (pd, J(H–H) = 8.6 Hz, 4H, HorthoPhCN), 7.90 (pd, J(H–H) = 8.9 Hz, 4H, HmetaPhNC), 7.84 (pd, J(H–H) = 8.6 Hz, 4H, HmetaPhCN), 7.81–7.75 (m br, 8H, Ph(dppp)), 7.50–7.46 (m br, 12H, Ph(dppp)), 7.38 (pd, J(H–H) = 8.8 Hz, 4H, HorthoPhNC), 3.16 (m, 4H, CH_2_–P(dppp)), 2.40 (m, 2H, C–CH_2_–C(dppp)).

^31^P{^1^H} NMR (162 MHz, CD_3_CN): −15.8 s (J(Pt–P) = 2465 Hz).

^31^P{^1^H} NMR (162 MHz, CD_2_Cl_2_): −17.2 s (J(Pt–P) = 2500 Hz).

IR (cm^–1^): 2230 (C–C≡N), 2218 (M-C≡N), 1486 (dppp), 1438 (dppp), 1250 (OTf), 1153 (OTf), 1104 (dppp), 751 (dppp).

MS (ESI+) *m*/*z*: {[Pt(dppp)(CN(C_6_H_4_)-N=N-(C_6_H_4_)CN)_2_]}^2+^, 535.63 (calc: 535.63); {[Pt(dppp)(CN)(CN(C_6_H_4_)-N=N-(C_6_H_4_)CN)]}^+^ 865.19 (calc: 865.19); {[Pt(dppp)(CN(C_6_H_4_)-N=N-(C_6_H_4_)CN)_2_](OTf)}^+^, 1220.22 (calc: 1220.22).

UV–vis [λ_max_, nm (ε, M^–1^ cm^–1^)]: (CH_3_CN) 330 (87,800), 451 (2190); (CH_2_Cl_2_) 332 (79,000), 462 (1830).

### After Irradiation

^1^H NMR (400 MHz, CD_3_CN) (***cis*–*trans***): 7.69–7.66 (superimposed with phosphane signals, HmetaPhCN_***cis***_), 6.98 (pd, J(H–H) = 8.5 Hz, HmetaPhNC_***cis***_), 6.95 (pd, J(H–H) = 8.7 Hz, HorthoPhCN_***cis***_), 6.86 (pd, J(H–H) = 8.6 Hz, HorthoPhNC_***cis***_). All the resonances of the ligand in ***trans*** are superimposed with those corresponding to the analogous ***trans–trans*** photoisomer.

^1^H NMR (400 MHz, CD_2_Cl_2_) (***cis*–*trans***): 8.00 (superimposed with signals of ***trans*–*trans***, HorthoPhCN_***trans***_), 7.90 (superimposed with signals of ***trans*–*trans***, HmetaPhNC_***trans***_), 7.84 (superimposed with signals of ***trans*–*trans***, HmetaPhCN_***trans***_), 7.81–7.71 (m br, superimposed with signals of ***trans*–*trans***, Ph(dppp)), 7.57 (pd, J(H–H) = 8.3 Hz, HorthoPhNC_***cis***_), 7.50–7.44 (m br, superimposed with signals of ***trans*–*trans***, Ph(dppp)), 7.34 (pd, J(H–H) = 8.8 Hz, HorthoPhNC_***trans***_), 7.16 (pd, J(H–H) = 8.6 Hz, HmetaPhNC_***cis***_), 6.84 (pd, J(H–H) = 8.1 Hz, HmetaPhCN_***cis***_), 6.75 (pd, J(H–H) = 8.4 Hz, HorthoPhNC_***cis***_), 3.16 (m, superimposed with signals of ***trans*–*trans***, CH_2_–P(dppp)), 2.40 (m, superimposed with signals of ***trans*–*trans***, C–CH_2_–C(dppp)).

^31^P{^1^H} NMR (162 MHz, CD_2_Cl_2_): −17.24 s (^195^Pt satellites superimposed with those of ***trans*–*trans***) (***cis*–*trans***).

#### [Pd(dppp)(**iso-Ph**)_2_](OTf)_2_

The procedure used was analogue to that described for [Pd(dppp)(**iso-cyano**)_2_](OTf)_2_. Thirty mg (0.035 mmol, 1 equiv) of [Pd(dppp)(H_2_O)_2_](OTf)_2_ was reacted with 14.6 mg (0.070 mmol, 2 equiv) of **iso-Ph** to give an 76% yield of a red–orange solid (33 mg, 0.027 mmol).

### Before Irradiation (***trans*–*trans***)

^1^H NMR (400 MHz, CD_3_CN): 7.96–7.92 (m, 4H, HmetaPh), 7.91 (ps, 4H, J(H–H) = 9.1, HmetaPhNC), 7.68–7.63 (m, 8H, Ph(dppp)), 7.63–7.59(m, 6H, HorthoPh + HparaPh), 7.59–7.47 (m, 12H, Ph(dppp)), 7.27 (pd, J(H–H) = 8.8, 4H, HorthoPhNC), 2.93 (m, 4H, CH_2_–P(dppp)), 2.32 (m, 2H, C–CH_2_–C(dppp)).

^1^H NMR (400 MHz, CD_2_Cl_2_): 7.94–7.91 (m, 4H, HmetaPh), 7.85 (pd, J(H–H) = 9.1, 4H, HmetaPhNC), 7.79–7.73 (m, 8H, Ph(dppp)), 7.55–7.53 (m, 6H, HorthoPh + HparaPh), 7.47–7.44 (m, 12H, Ph(dppp)), 7.34 (pd, J(H–H) = 8.8 Hz, 4H, HorthoPhNC), 3.03 (m, 4H, CH_2_–P(dppp)), 2.38 (m, 2H, C–CH_2_–C(dppp)).

^31^P{^1^H} NMR (162 MHz, CD_3_CN): 0.8 s.

^31^P{^1^H} NMR (162 MHz, CD_2_Cl_2_): −0.8 s.

IR (cm^–1^): 2225 (M-C≡N), 1485 (dppp), 1435 (dppp), 1101 (dppp), 1247 (OTf), 1152 (OTf), 751 (dppp).

MS (ESI+) *m*/*z*: {[Pd(dppp)(CN(C_6_H_4_)-N=N-(C_6_H_5_))]}^2+^, 362.57 (calc: 362.57); {[Pd(dppp)(CN(C_6_H_4_)–N=N-(C_6_H_5_))_2_]}^2+^, 466.11 (calc: 466.11); {[Pd(dppp)(CN)(CN(C_6_H_4_)-N=N-(C_6_H_5_))]}^+^ 751.14 (calc: 751.14); {[Pd(dppp)(CN(C_6_H_4_)-N=N-(C_6_H_5_))_2_](OH)}^+^, 949.22 (calc: 949.22).

UV–vis [λ_max_, nm (ε, M^–1^ cm^–1^)]: (CH_3_CN) 326 (65,930), 433 (1550); (CH_2_Cl_2_) 330 (59,310), 432 (2340).

### After Irradiation

^1^H NMR (400 MHz, CD_3_CN) (***cis*–*trans***): 7.35–7.30 (m, HmetaPh_***cis***_), 7.27–7.23 (superimposed with HorthoPhNC of ***trans*–*trans***, HparaPh_***cis***_), 6.97 (pd, J(H–H) = 8.4, HorthoPhNC_***cis***_), 6.84 (ps, J(H–H) = 8.9, HmetaPhNC_***cis***_), 6.83–6.80 (m, HorthoPh_***cis***_). All the resonances of the ligand in ***trans*** are superimposed with those corresponding to the analogous ***trans*–*trans*** photoisomer.

^1^H NMR (400 MHz, CD_2_Cl_2_): (***cis*–*trans***): 7.94–7.91 (superimposed with signals of ***trans*–*trans***, HmetaPh_***trans***_), 7.84 (pd, J(H–H) = 9.2 Hz, HmetaPhNC_***trans***_), 7.79–7.67 (m br, superimposed with signals of ***trans*–*trans*** and ***cis*–*cis***, Ph(dppp)), 7.56–7.52 (m, superimposed with signals of ***trans*–*trans***, HorthoPh_***tran*s**_ + HparaPh_***trans***_), 7.47–7.37 (m br, superimposed with signals of ***trans*–*trans*** and *cis–cis*, Ph(dppp)), 7.31–7.26 (m, superimposed with signals of ***cis*–*cis***, HorthoPhNC_***trans***_ + HmetaPh_***cis***_), 7.23–7.17 (m, superimposed with signals of ***cis*–*cis***, HparaPh_***cis***_), 7.09 (pd, J(H–H) = 8.7 Hz, HmetaPhNC_***cis***_), 6.78 (pd br, J(H–H) = 7.7 Hz, superimposed with signals of ***cis*–*cis***, HorthoPh_***cis***_), 6.73 (pd, J(H–H) = 8.1 Hz, superimposed with signals of ***cis*–*cis***, HorthoPhNC_***cis***_), 3.03 (m, superimposed with signals of ***trans*–*trans*** and ***cis*–*cis***, CH_2_–P(dppp)), 2.38 (m, superimposed with signals of ***trans*–*trans*** and ***cis*–*cis***, C–CH_2_–C(dppp)).

(***cis***–***cis***): 7.79–7.67 (m br, superimposed with signals of ***trans*–*trans*** and ***trans*–*cis***, Ph(dppp)), 7.47–7.37 (m br, superimposed with signals of ***trans*–*trans*** and ***trans*–*cis***, Ph(dppp)), 7.31–7.26 (m, superimposed with signals of ***trans*–*cis***, HmetaPh), 7.23–7.17 (m, superimposed with signals of ***cis*–*trans***, HparaPh), 7.05 (pd, J(H–H) = 8.7 Hz, HmetaPhNC), 6.78 (pd br, J(H–H) = 7.7 Hz, superimposed with signals of ***cis*–*trans***, HorthoPh), 6.71 (pd, superimposed with signals of ***cis*–*trans***, HorthoPhNC), 3.03 (m, superimposed with signals of ***trans*–*trans*** and ***cis*–*trans***, CH_2_–P(dppp)), 2.38 (m, superimposed with signals of ***trans*–*trans*** and ***cis*–*trans***, C–CH_2_–C(dppp)).

^31^P{^1^H} NMR (162 MHz, CD_2_Cl_2_): −0.96 s, −1.00 s (***cis*–*trans***), −1.10 s (***cis*–*cis***).

#### [Pt(dppp)(**iso-Ph**)_2_](OTf)_2_

The procedure used was analogue to that described for [Pd(dppp)(**iso-cyano**)_2_](OTf)_2_. Thirty mg (0.032 mmol, 1 equiv) of [Pt(dppp)(H_2_O)_2_](OTf)_2_ was reacted with 13.20 mg (0.064 mmol, 2 equiv) of **iso-Ph** to give an 78% yield of a red-orange solid (31 mg, 0.023 mmol).

### Before Irradiation (***trans*–*trans***)

^1^H NMR (400 MHz, CD_3_CN): 7.95–7.93 (m, 4H, HmetaPh), 7.91 (pd, J(H–H) = 9.0, 4H, HmetaPhNC), 7.70–7.65 (m br, 8H, Ph(dppp)), 7.63–7.59 (m, 6H, HorthoPh + HparaPh), 7.58–7.50 (m br, 12H, Ph(dppp)), 7.25 (pd, J(H–H) = 8.4, 4H, HorthoPhNC), 3.04 (m, 4H, CH_2_–P(dppp)), 2.34 (m, 2H, C–CH_2_–C(dppp)) ppm.

^1^H NMR (400 MHz, CD_2_Cl_2_): 7.95–7.91 (m, 4H, HmetaPh), 7.86 (pd, J = 8.8, 4H, HmetaPhNC), 7.81–7.75 (m, 8H, Ph(dppp)), 7.56–7.53 (m, 6H, HorthoPh + HparaPh), 7.49–7.47 (m, 12H, Ph(dppp)), 7.33 (pd, J(H–H) = 8.8 Hz, 4H, HorthoPhNC), 3.17 (m, 4H, CH_2_–P(dppp)), 2.40 (m, 2H, C–CH_2_–C(dppp)).

^31^P{^1^H} NMR (162 MHz, CD_3_CN): −15.8 s (J(Pt–P) = 2410 Hz).

^31^P{^1^H} NMR (162 MHz, CD_2_Cl_2_): −17.1 s (J(Pt–P) = 2500 Hz).

IR (cm^–1^): 2225 (M-C≡N), 1483 (dppp), 1434 (dppp), 1248 (OTf), 1152 (OTf), 1104 (dppp), 750 (dppp).

MS (ESI+) *m*/*z*: {[Pt(dppp)(CN(C_6_H_4_)-N=N-(C_6_H_5_))(CH_3_CN)]}^2+^, 427.61 (calc: 427.61); {[Pt(dppp)(CN(C_6_H_4_)-N=N-(C_6_H_5_)_2_]}^2+^, 510.64 (calc: 510.64); {[Pt(dppp)(CN)(CN(C_6_H_4_)-N=N-(C_6_H_5_))]}^+^ 840.20 (calc: 840.20); {[Pt(dppp)(CN(C_6_H_4_)-N=N-(C_6_H_5_))_2_](OTf)}^+^, 1170.23 (calc: 1170.23).

UV–vis [λ_max_, nm (ε, M^–1^ cm^–1^)]: (CH_3_CN) 334 (67,570), 457 (2090); (CH_2_Cl_2_) 333 (59,290), 466 (1300).

### After Irradiation

^1^H NMR (400 MHz, CD_3_CN) (***cis*–*trans***): 7.35–7.30 (m, HmetaPh_***cis***_), 7.26–7.22 (superimposed with HorthoPhNC of ***trans*–*trans***, HparaPh_***cis***_), 6.96 (pd, J(H–H) = 834, HorthoPhNC_***cis***_), 6.84 (ps, J(H–H) = 8.6 HmetaPhNC_***cis***_), 6.83–6.80 (m, HorthoPh_***cis***_). All the resonances of the ligand in ***trans*** are superimposed with those corresponding to the analogous ***trans*–*****trans*** photoisomer.

^1^H NMR (500 MHz, CD_2_Cl_2_): (***cis*–*trans***): 7.95–7.91 (superimposed with signals of ***trans*–*trans***, HmetaPh_***trans***_), 7.83 (superimposed with signals of ***trans*–*trans***, HmetaPhNC_***trans***_), 7.78–7.68 (m br, superimposed with signals of ***trans*–*trans*** and ***cis*–*cis***, Ph(dppp)), 7.55–7.52 (m, superimposed with signals of ***trans*–*trans***, HorthoPh_***trans***_ + HparaPh_***trans***_), 7.47–7.37 (m br, superimposed with signals of ***trans*–*trans*** and ***cis*–*cis***, Ph(dppp)), 7.31–7.26 (m, superimposed with signals of ***cis*–*cis***, HorthoPhNC_***trans***_ + HmetaPh_***cis***_), 7.21–7.18 (m, superimposed with signals of ***cis*–*cis***, HparaPh_***cis***_), 7.08 (pd, J(H–H) = 8.5 Hz, HmetaPhNC_***cis***_), 6.82 (br, superimposed with signals of ***cis*–*cis***, HorthoPh_***cis***_), 6.72 (pd br, J(H–H) = 8.4 Hz, superimposed with signals of ***cis*–*cis***, HorthoPhNC_***cis***_), 3.03 (m, superimposed with signals of ***trans*–*trans*** and ***cis*–*cis***, CH_2_–P(dppp)), 2.38 (m, superimposed with signals of ***trans*–*trans*** and ***cis*–*cis***, C–CH_2_–C(dppp)).

(***cis***–***cis***): 7.78–7.68 (m br, superimposed with signals of ***trans*–*trans*** and ***trans*–*cis***, Ph(dppp)), 7.47–7.37 (m br, superimposed with signals of ***trans*–*trans*** and ***trans*–*cis***, Ph(dppp)), 7.31–7.26 (m, superimposed with signals of ***trans*–*cis***, HmetaPh), 7.21–7.18 (m, superimposed with signals of ***cis*–*trans***, HparaPh), 7.04 (pd, J(H–H) = 8.7 Hz, HmetaPhNC), 6.82 (br, superimposed with signals of ***cis*–*trans***, HorthoPh), 6.72 (pd, J(H–H) = 8.4 Hz, superimposed with signals of ***cis*–*trans***, HorthoPhNC), 3.03 (m, superimposed with signals of ***trans*–*trans*** and ***cis*–*trans***, CH_2_–P(dppp)), 2.38 (m, superimposed with signals of ***trans*–*trans*** and ***cis*–*trans***, C–CH_2_–C(dppp)).

^31^P{^1^H} NMR (162 MHz, CD_3_CN): −17.14 s (J(Pt–P) = 2500 Hz) (***cis*–*trans***), −17.23 s (***cis*–*cis***).

#### [{Pd_2_(tpbz)}(**iso-Ph**)_4_](OTf)_4_

The procedure used was analogue to that described for [Pd(dppp)(**iso-cyano**)_2_](OTf)_2_. Twenty mg (0.011 mmol, 1 equiv) of [Pd_2_(tpbz)(CH_3_CN)_4_](OTf)_4_ were reacted with 9.2 mg (0.047 mmol, 4 equiv) of **iso-Ph** to give an 78% yield of an orange solid (22 mg, 0.009 mmol).

^1^H NMR (500 MHz, CD_2_Cl_2_): 7.94–7.90 (m, 16H, HmetaPh + HmetaPhNC), 7.69–7.64 (m, 16H, Ph(tpbz)), 7.55–7.52 (m, 20H, HorthoPh + HparaPh + HorthoPhNC), 7.47–7.45 (m, 24H, Ph(tpbz)), 7.22 (pt, J(H–H) = 9.2 Hz, 2H, H(tpbz)).

^31^P{^1^H} NMR (202 MHz, CD_2_Cl_2_): 53.4 ppm.

IR (cm^–1^): 2224 (C≡N), 1483 (tpbz), 1438 (tpbz), 1248 (OTf), 1147 (OTf), 1098 (tpbz), 746 (tpbz).

HRMS (ESI+) *m*/*z*: {[{Pd_2_(tpbz)}(CN(C_6_H_4_)-N=N-(C_6_H_5_))_4_]}^4+^, 464.0881 (calc: 464.0884), {[{Pd_2_(tpbz)}(CN(C_6_H_4_)-N=N-(C_6_H_5_))_4_](OTf)}^3+^, 668.4355 (calc: 668.4352),{[{Pd_2_(tpbz)}(CN(C_6_H_4_)-N=N-(C_6_H_5_))_4_](OTf)_2_}^2+^, 1077.1289 (calc: 1077.1288).

UV–vis [λ_max_, nm (ε, M^–1^ cm^–1^)]: (CH_2_Cl_2_) 333 (130,560), 463 (4930).

#### [{Pt_2_(tpbz)}(**iso-Ph**)_4_](OTf)_4_

The procedure used was analogue to that described for [Pd(dppp)(**iso-cyano**)_2_](OTf)_2_. Twenty mg (0.010 mmol, 1 equiv) of [{Pt_2_(tpbz)}(CH_3_CN)_4_](OTf)_4_ were reacted with 8.4 mg (0.041 mmol, 4 equiv) of **iso-Ph** to give an 80% yield of a deep orange solid (21.0 mg, 0.008 mmol).

^1^H NMR (500 MHz, CD_2_Cl_2_): 7.94–7.91 (m, 16H, HmetaPh + HmetaPhNC), 7.73–7.69 (m, 19H, Ph(tpbz) + H(tpbz)), 7.60–7.50 (m, 36H, HorthoPh + HparaPh + Ph(tpbz)), 7.45 (pd, J(H–H) = 8.9 Hz, 8H, HorthoPhNC).

^31^P{^1^H} NMR (202 MHz, CD_2_Cl_2_): 35.0 s (J(Pt–P) = 2660 Hz).

IR (cm^–1^): 2205 (C≡N), 1483 (tpbz), 1438 (tpbz), 1248 (OTf), 1157 (OTf), 1104 (tpbz), 750 (tpbz).

HRMS (ESI+) *m*/*z*: {[{Pt_2_(tpbz)}(CN(C_6_H_4_)-N=N-(C_6_H_5_))_4_]}^4+^, 508.3673 (calc: 508.3685).

UV–vis [λ_max_, nm (ε, M^–1^ cm^–1^)]: (CH_2_Cl_2_) 339 (115,780), 497 (13,820).

## Supporting Information Available

NMR, MS and UV–vis spectra for the compounds prepared; XRD for the structures indicated in Figure 3; examples of time and irradiation-resolved UV–vis spectra of some of the complexes studied; plots of the solvent polarity trends of the activation entropies and enthalpies of the ligand utilized in this work; observed 1st-order rate constants for the spontaneous cis-to-trans isomerization reactions occurring on the compounds prepared as a function of solvent, temperature and pressure. Computed DFT Cartesian coordinates and absolute Gibbs energies of palladium and platinum complexes (PDF)
