# Supplementary material for: No Switching Cooperativity between Coordinated Azo Ligands on Complexes Having {MII(phosphane-κ2P)}2+ (M = Pd, Pt) Scaffolds
Source: Inorg Chem. 2024 Aug 21;63(35):16251–63. doi: 10.1021/acs.inorgchem.4c02169 (PMC11372757; doi:10.1021/acs.inorgchem.4c02169)
Supplement: Supplementary file 1 — ic4c02169_si_001.pdf [file ic4c02169_si_001.pdf]

No switching cooperativity between coordinated azo ligands on complexes having $\{M^{II}(\text{phosphane-}\kappa^2 P)\}^{2+}$ (M = Pd, Pt) scaffolds

Ot Raïch Panisello,[a] *Jesús Jover,*[a,b] *Cristina Puigjaner,*[c] *Montserrat Ferrer,*[a,d*] *Manuel Martínez.*[a,d*]

[a] Secció de Química Inorgànica, Departament de Química Inorgànica i Orgànica. Universitat de Barcelona, Martí i Franquès 1-11, 08028 Barcelona, Spain

[b] Institut de Química Teòrica i Computacional (IQTCUB), Universitat de Barcelona, 08028 Barcelona, Spain

[c] Unitat de Difracció de RX, Centres Científics i Tecnològics de la Universitat de Barcelona (CCiTUB). Universitat de Barcelona, Solé i Sabarís 1-3, 08028 Barcelona, Spain

[d] Institute of Nanoscience and Nanotechnology (IN2UB), Universitat de Barcelona, 08028 Barcelona, Spain

montse.ferrer@qi.ub.edu, manel.martinez@qi.ub.edu

a) b)

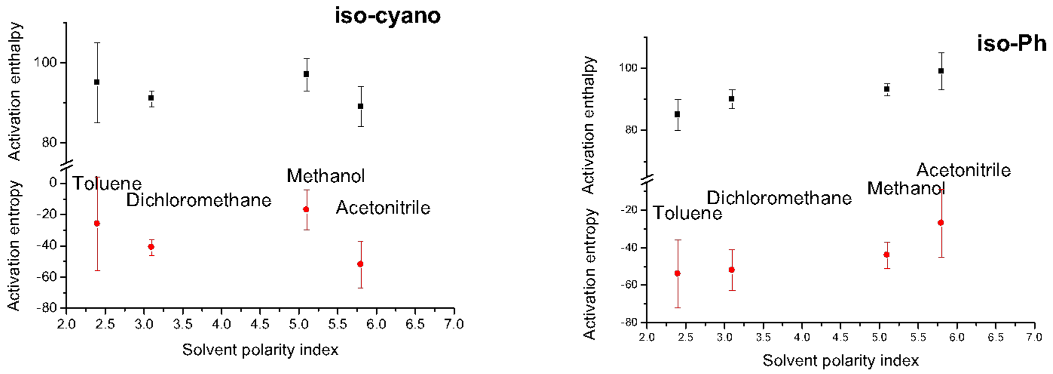

Figure S1.- Error bar plots of the values of the enthalpies and entropies of activation for the a) **iso-cyano** and b) **iso-Ph** ligands *versus* the polarity of the solvents used.

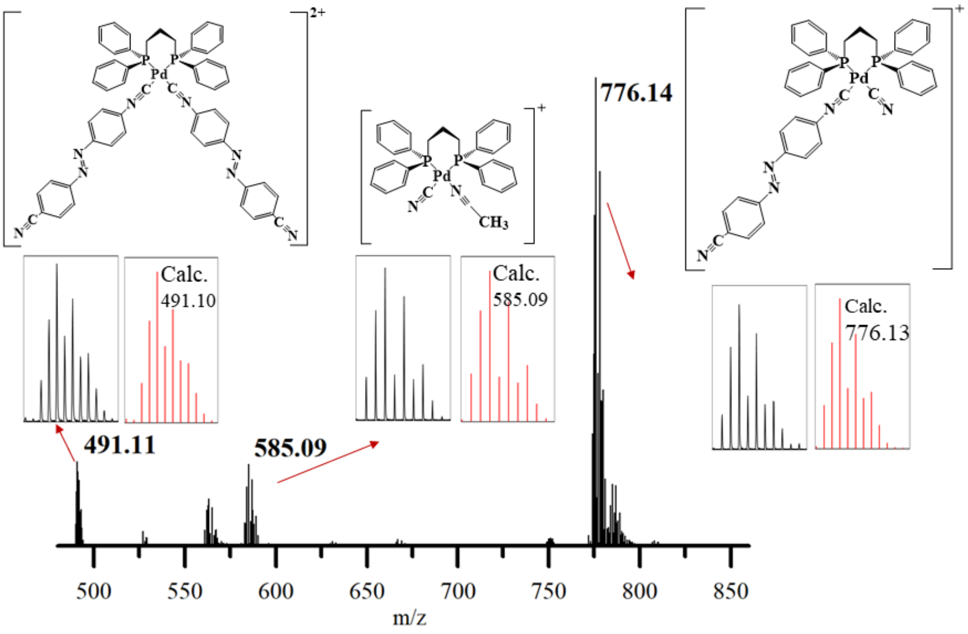

Figure S2.- ESI(+)-HRMS spectrum of [Pd(dppp)(**iso-cyano**)$_2$](OTf)$_2$.

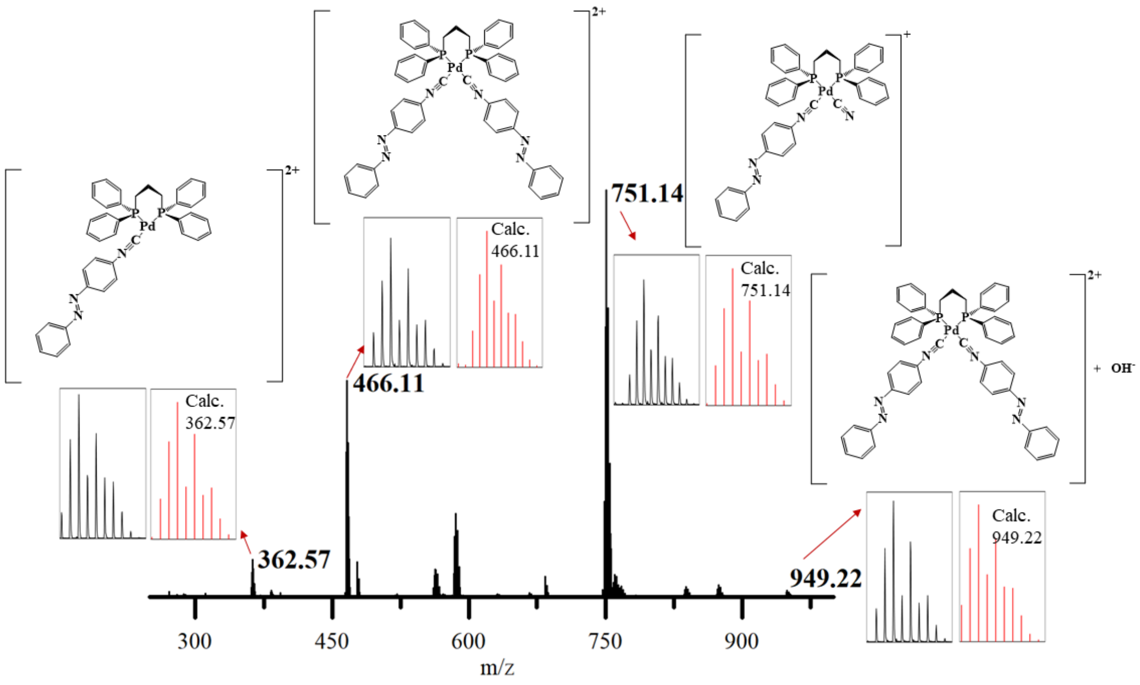

Figure S3.- ESI(+)-MS spectrum of [Pd(dppp)(**iso-Ph**)$_2$](OTf)$_2$.

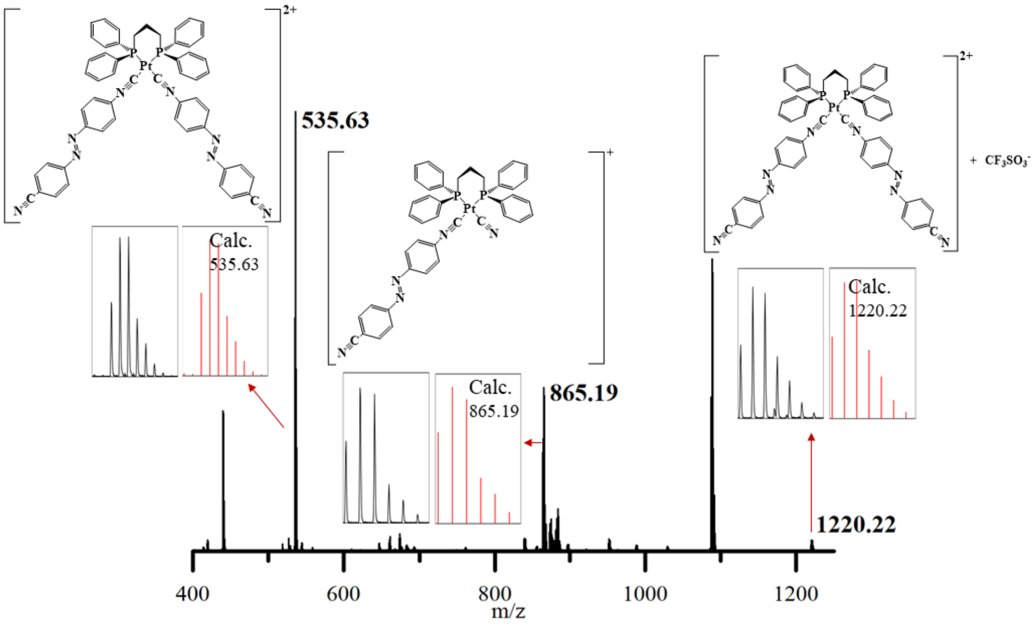

Figure S4.- ESI(+)-MS spectrum of [Pt(dppp)(**iso-cyano**)$_2$](OTf)$_2$.

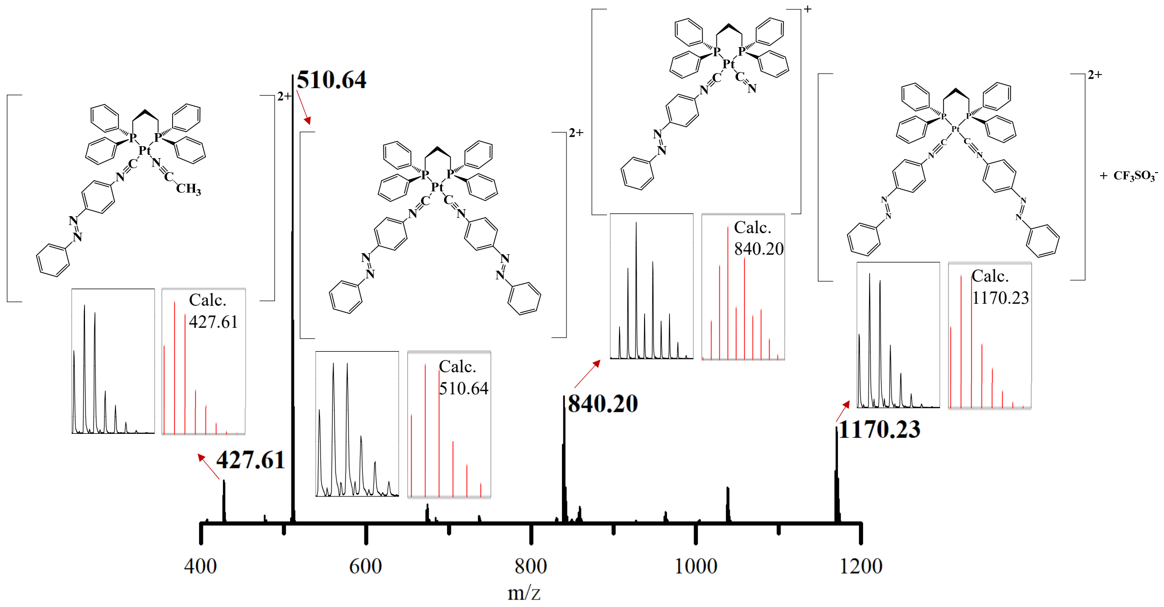

Figure S5.- ESI(+)-MS spectrum of [Pt(dppp)(**iso-Ph**)$_2$](OTf)$_2$.

a)

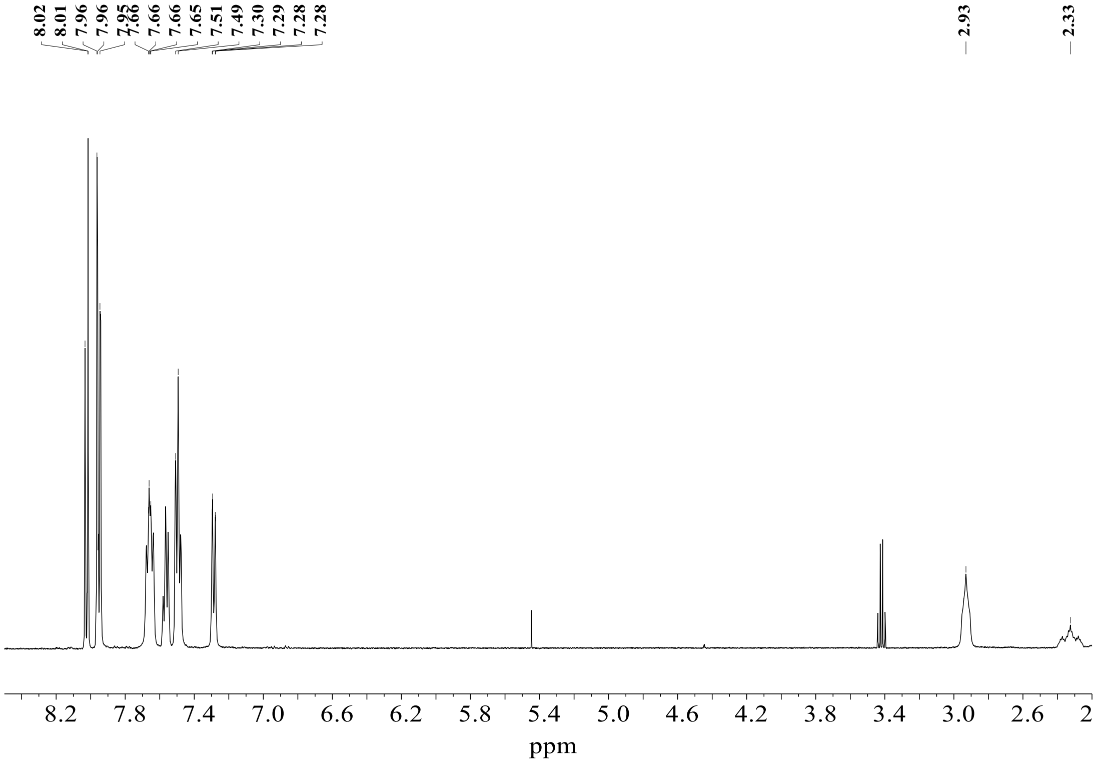

b)

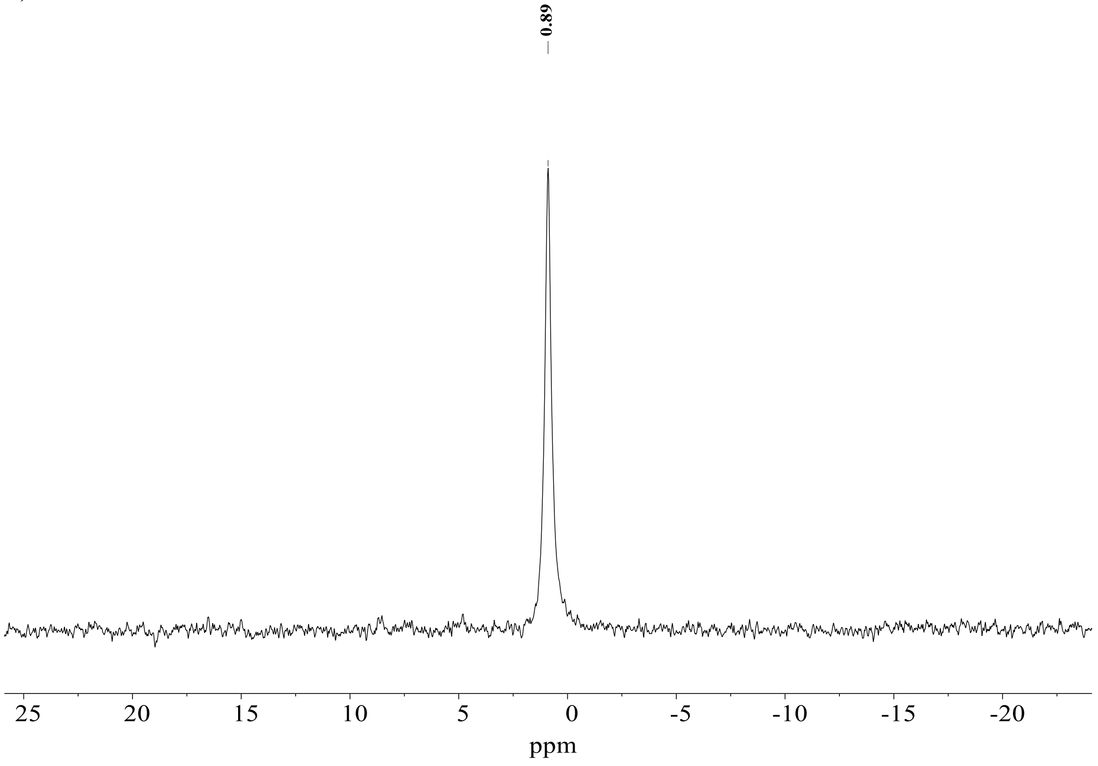

Figure S6.- a) ^{1}H and b) ^{31}P NMR (CD_3CN) spectra of [Pd(dppp)(**iso-cyano**)$_2$](OTf)$_2$.

a)

b)

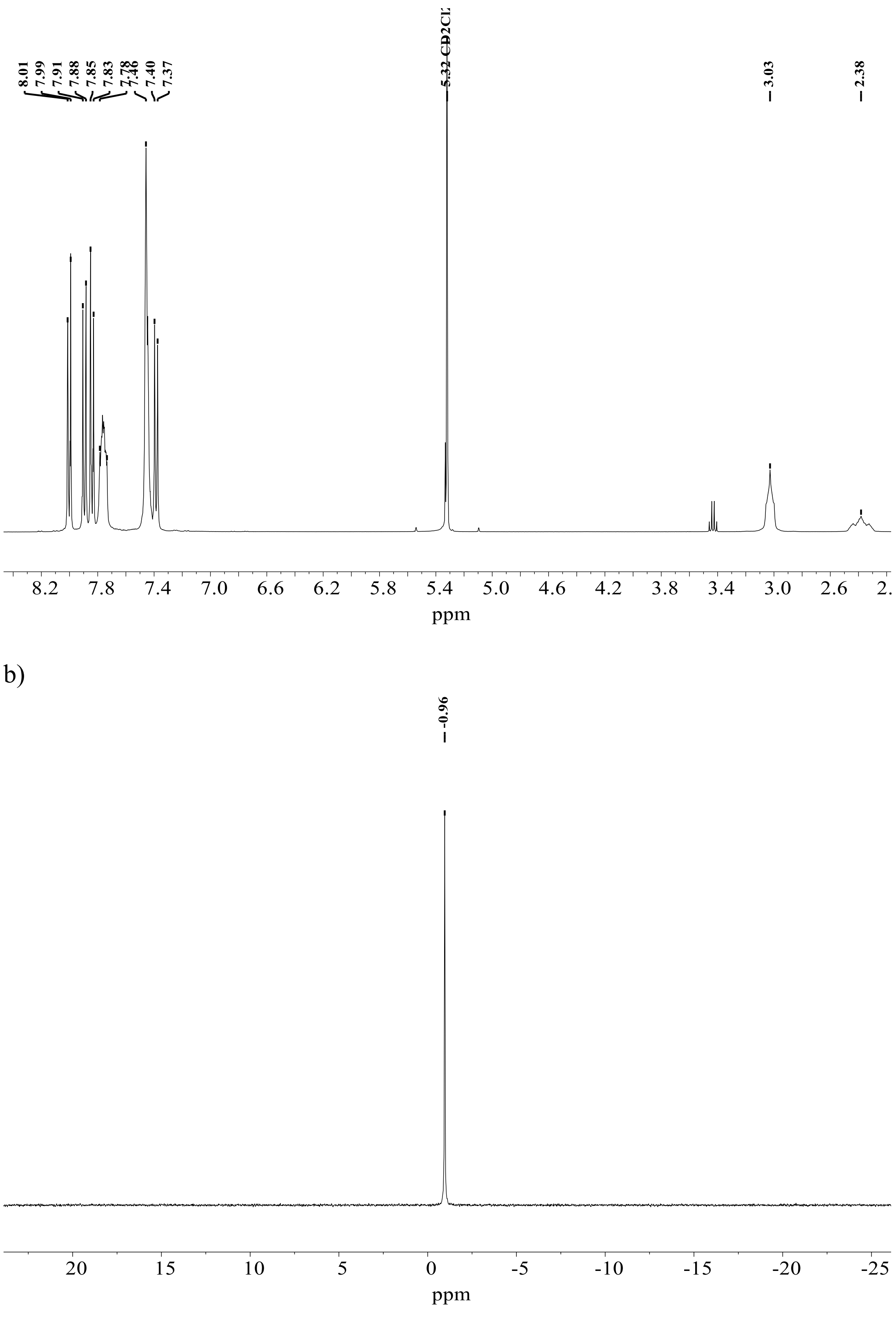

Figure S7.-a) ^{1}H and b) ^{31}P NMR (CD_2Cl_2) spectra of [Pd(dppp)(**iso-cyano**)$_2$](OTf)$_2$.

a)

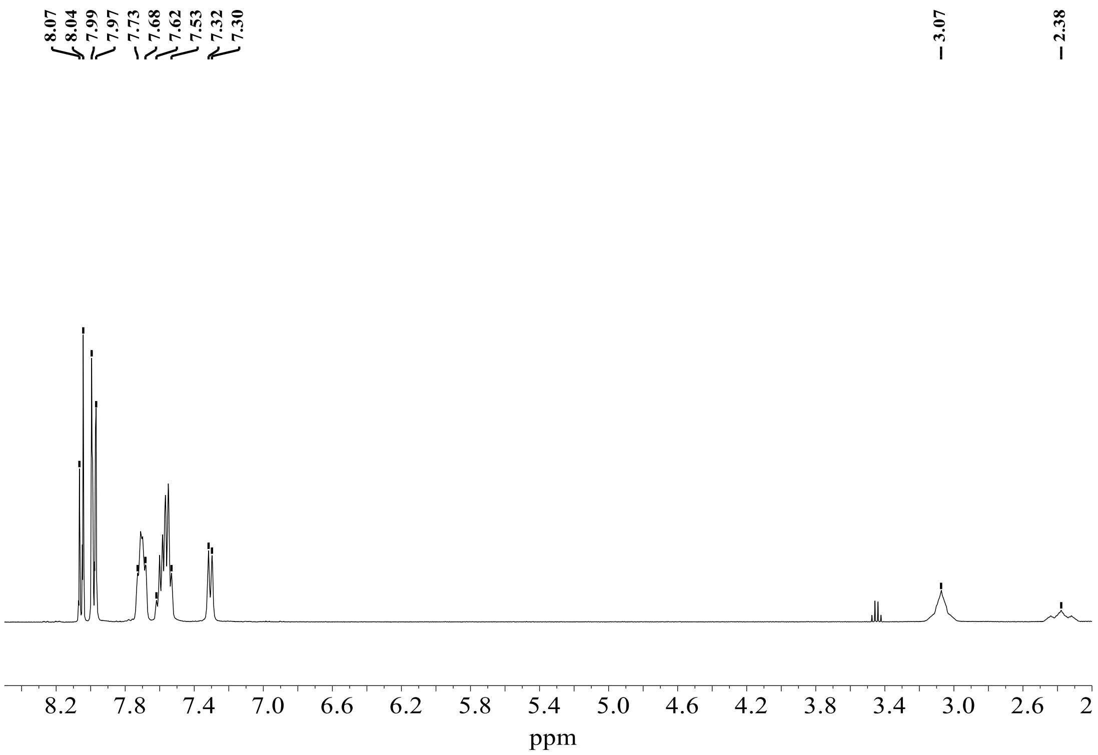

b)

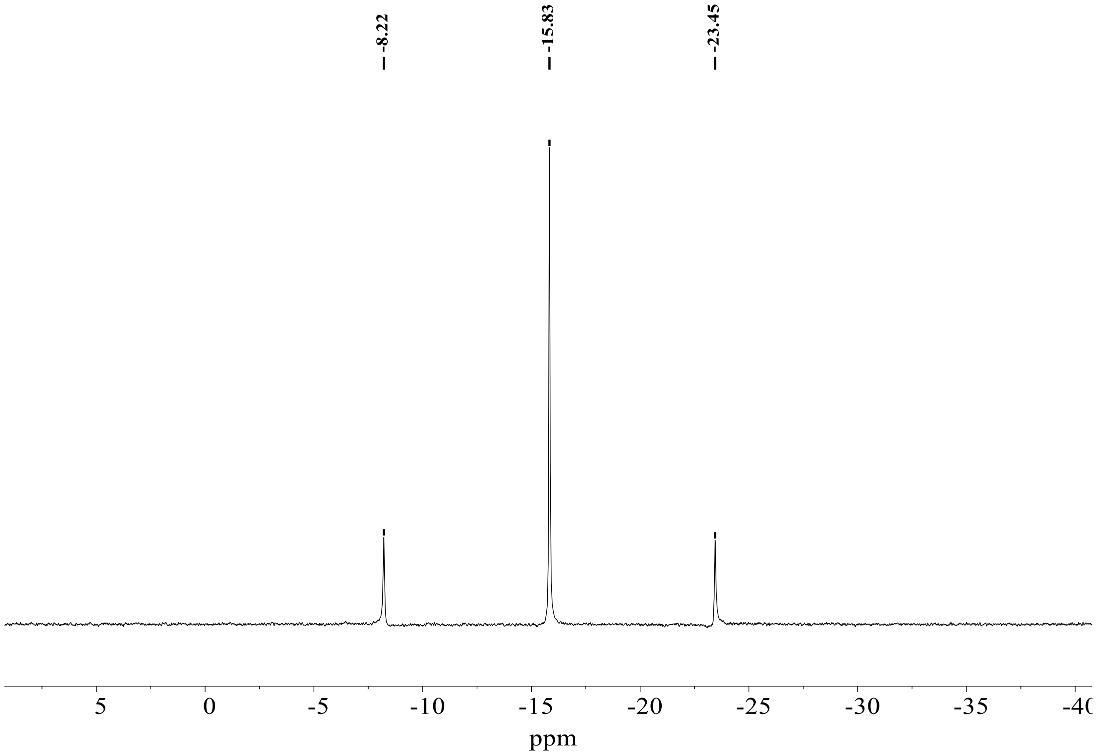

Figure S8.- a) ^{1}H and b) ^{31}P NMR (CD_3CN) spectra of [Pt(dppp)(**iso-cyano**)$_2$](OTf)$_2$.

a)

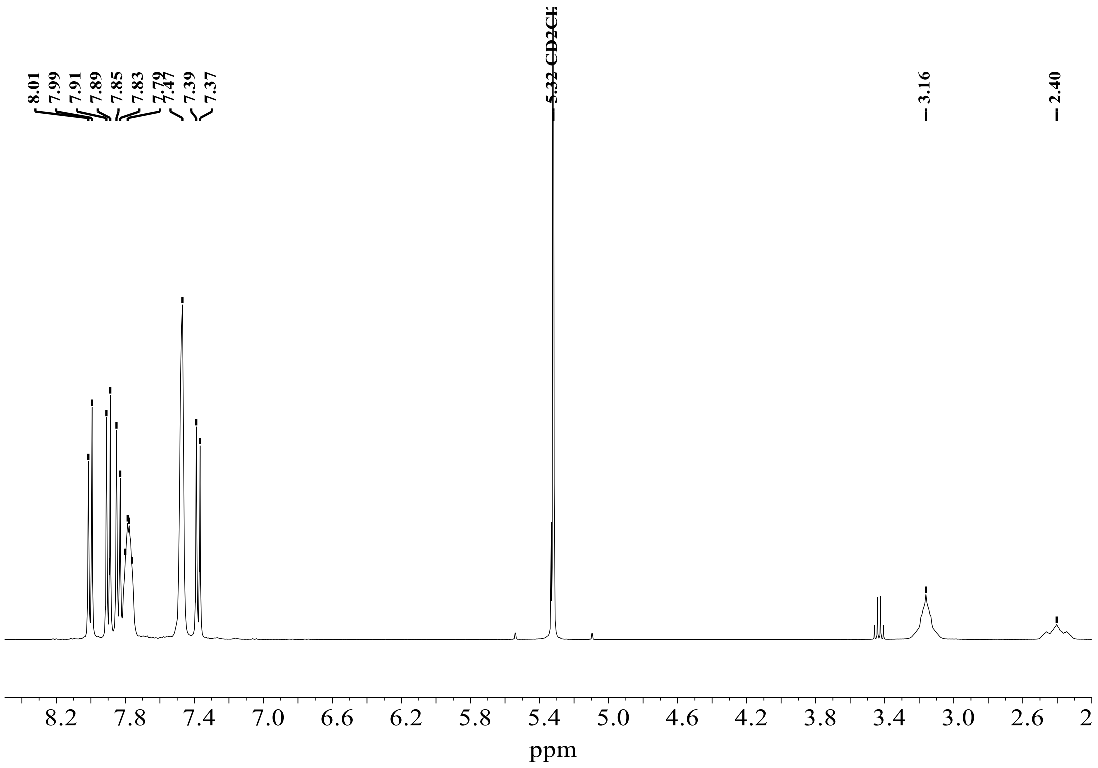

b)

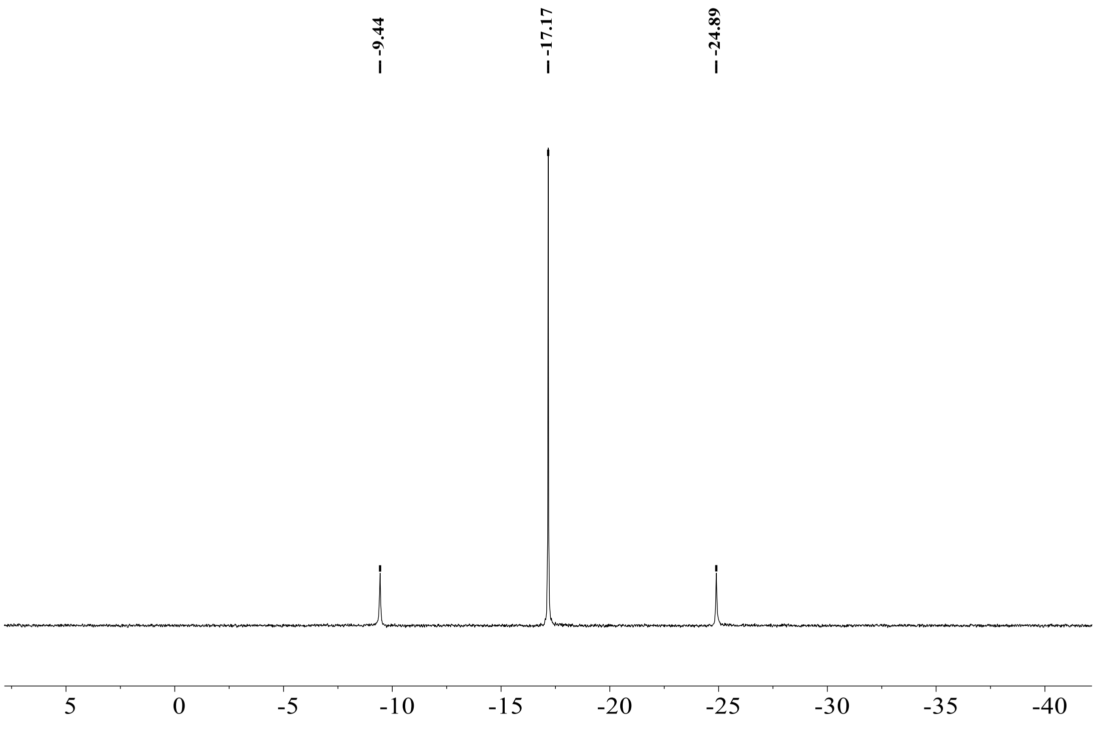

Figure S9.-a) ^{1}H and b) ^{31}P NMR (CD_2Cl_2) spectra of [Pt(dppp)(**iso-cyano**)$_2$](OTf)$_2$.

a)

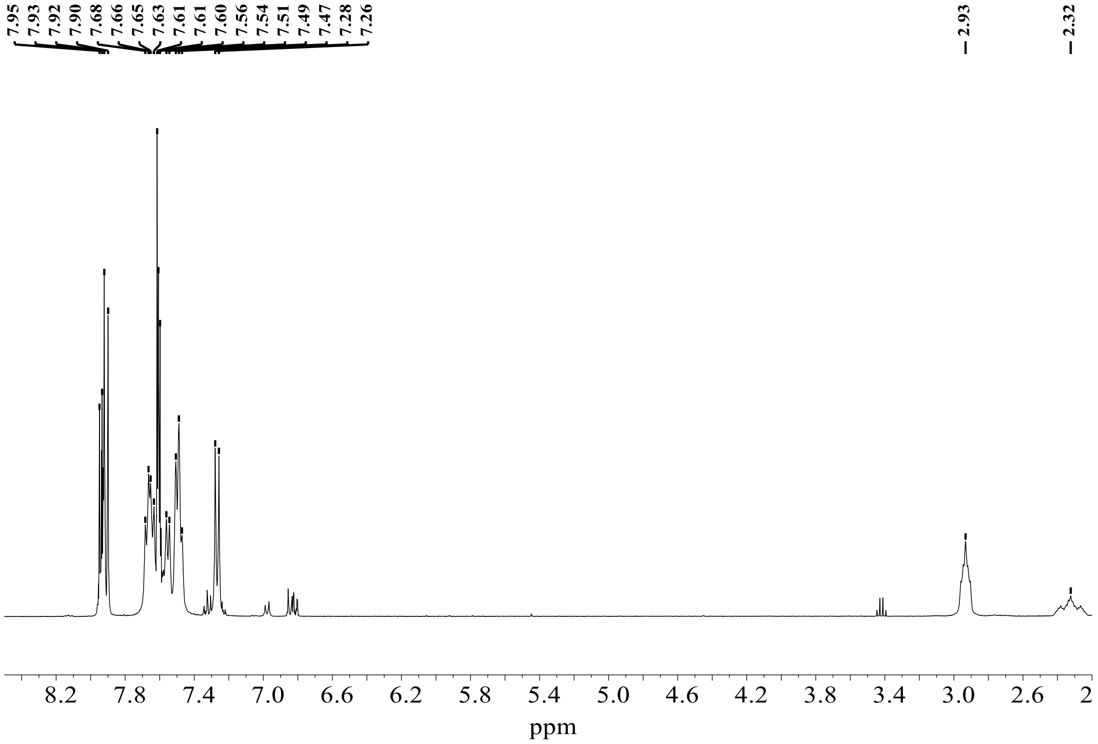

b)

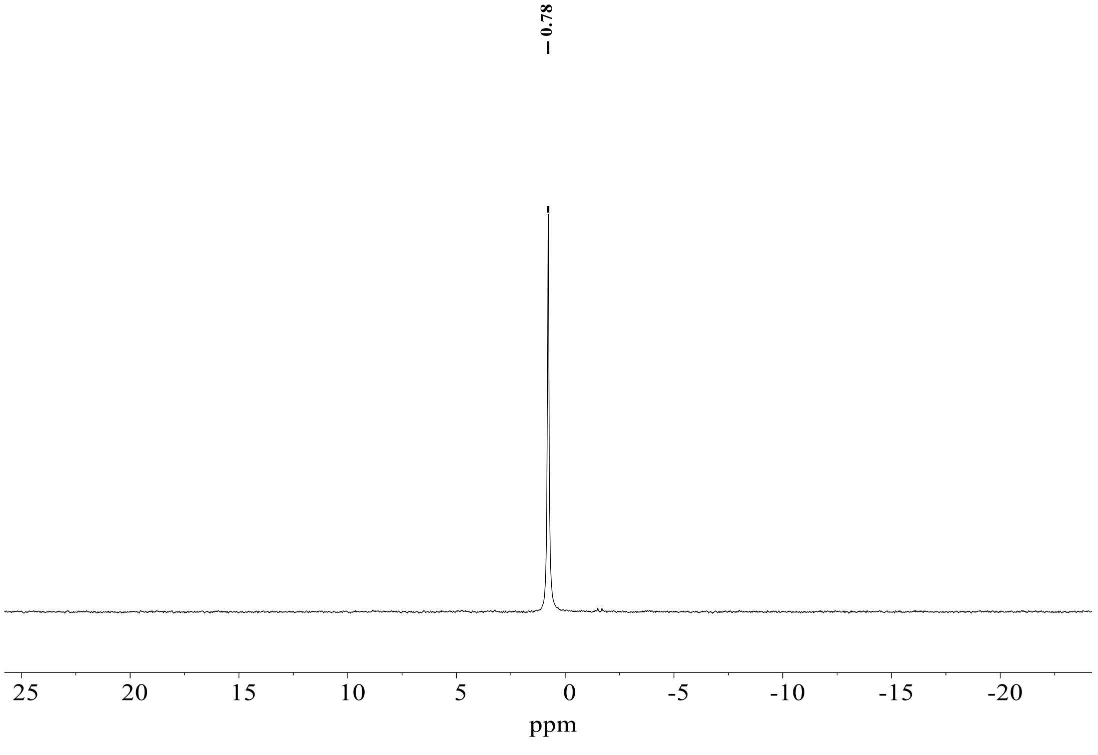

Figure S10.- a) ^{1}H and b) ^{31}P NMR (CD_3CN) spectra of [Pd(dppp)(**iso-Ph**)$_2$](OTf)$_2$.

a)

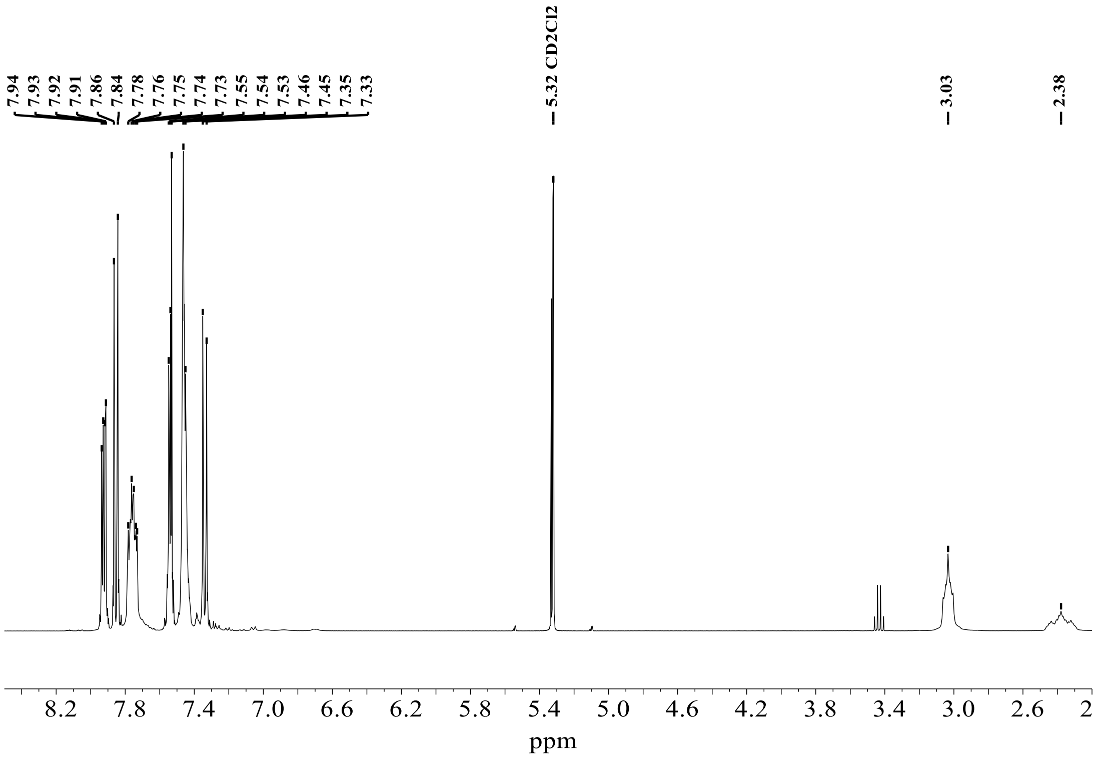

b)

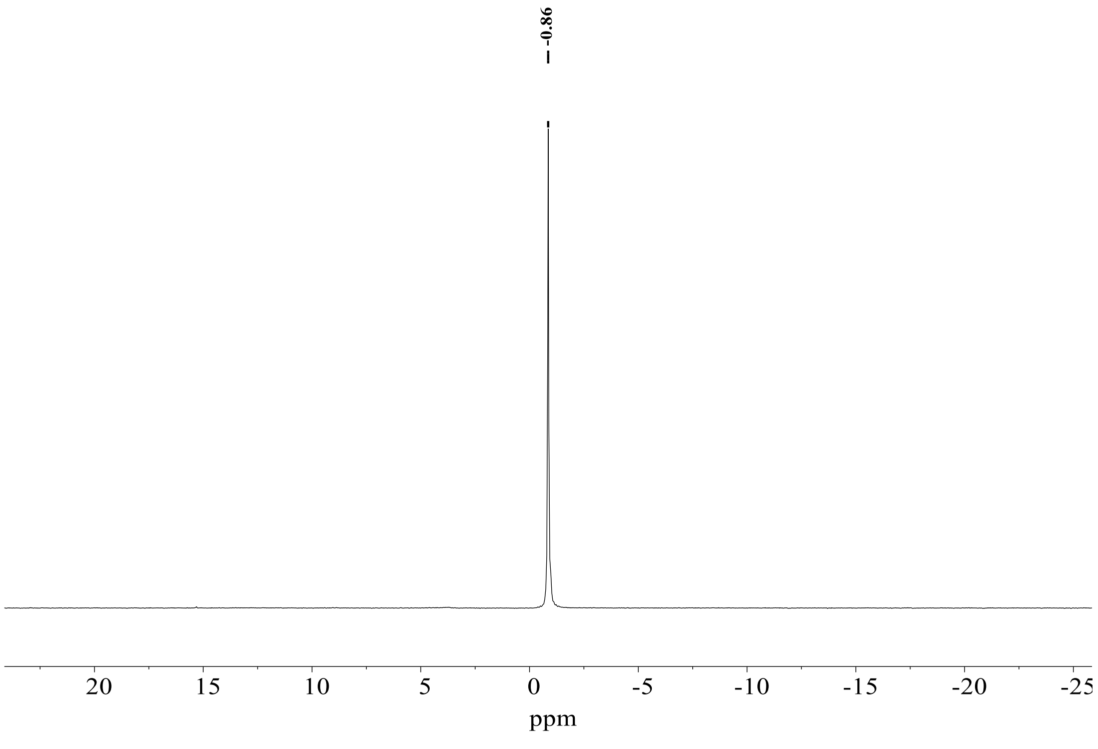

Figure S11.- a) ^{1}H and b) ^{31}P NMR (CD_2Cl_2) spectra of [Pd(dppp)(**iso-Ph**)$_2$](OTf)$_2$.

a)

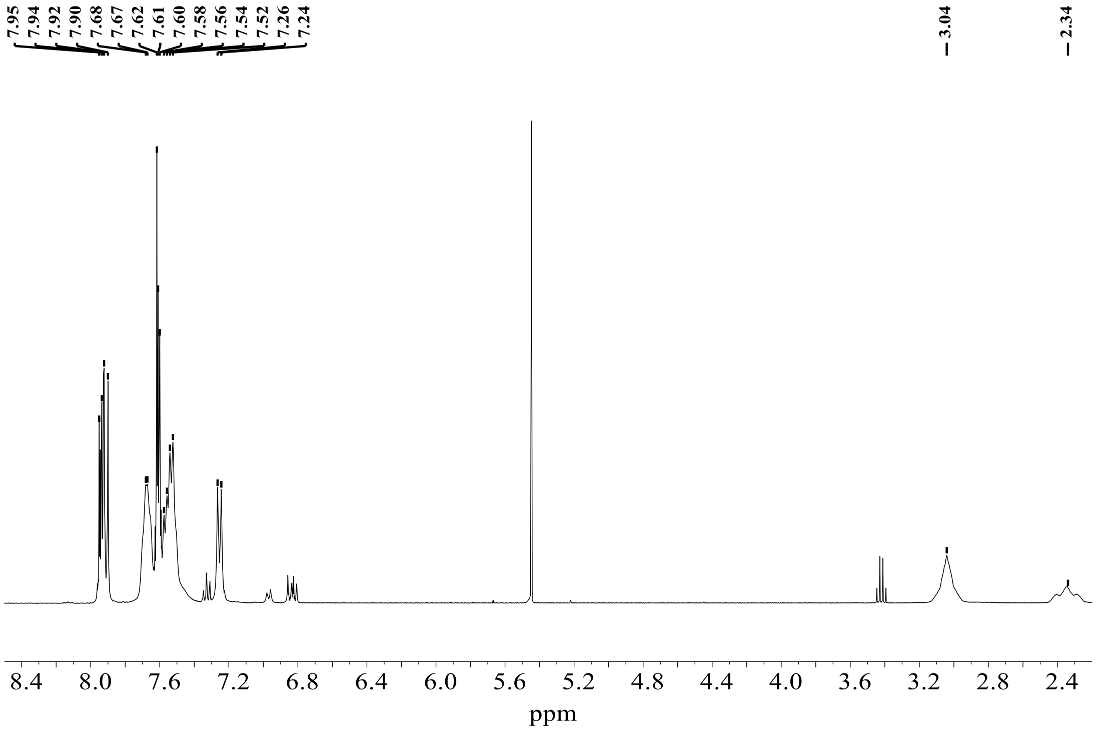

b)

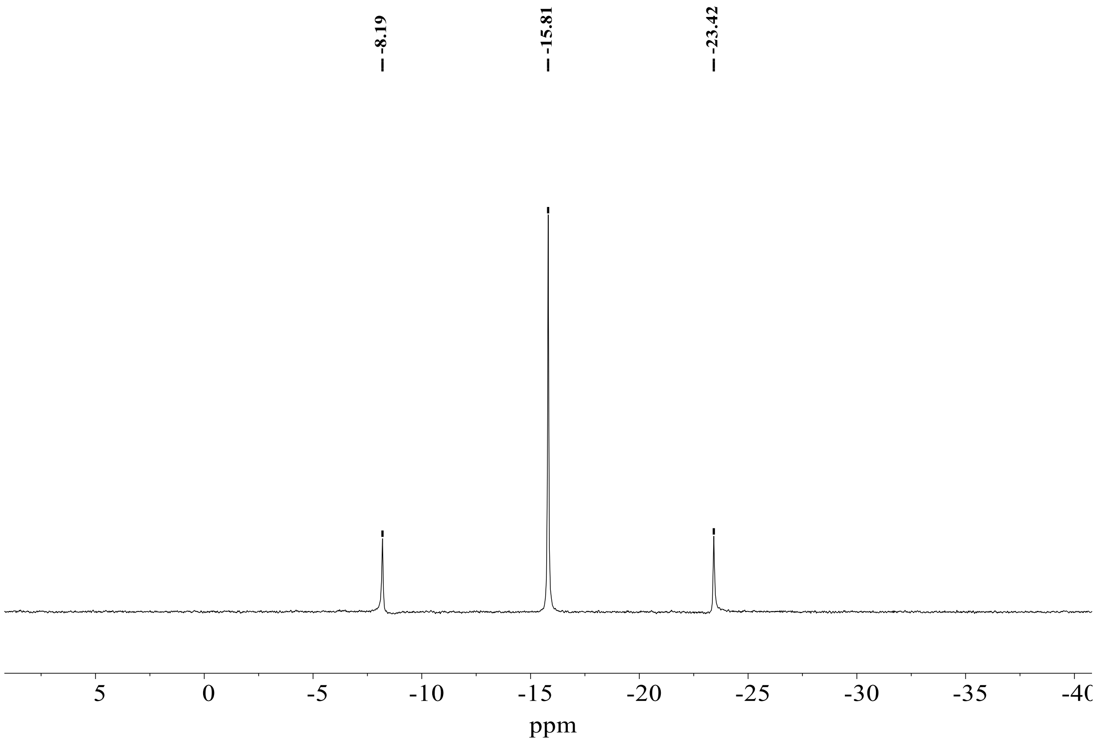

Figure S12.- a) ^{1}H and b) ^{31}P NMR (CD_3CN) spectra of [Pt(dppp)(**iso-Ph**)$_2$](OTf)$_2$.

a)

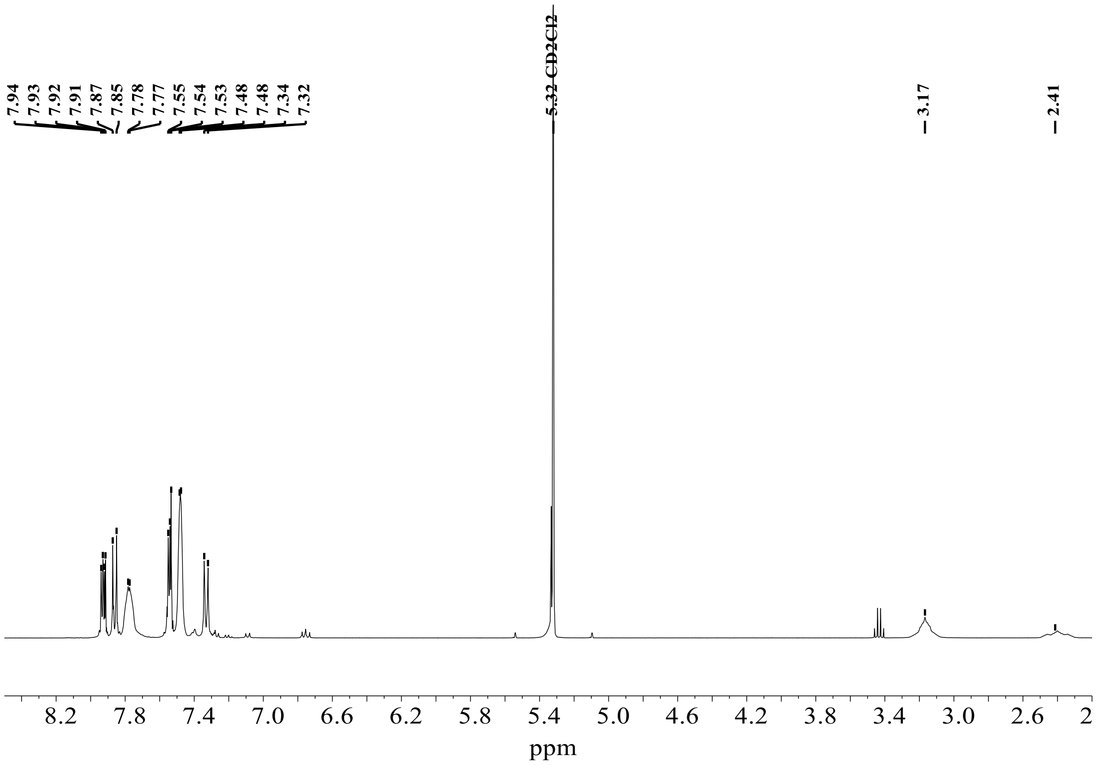

b)

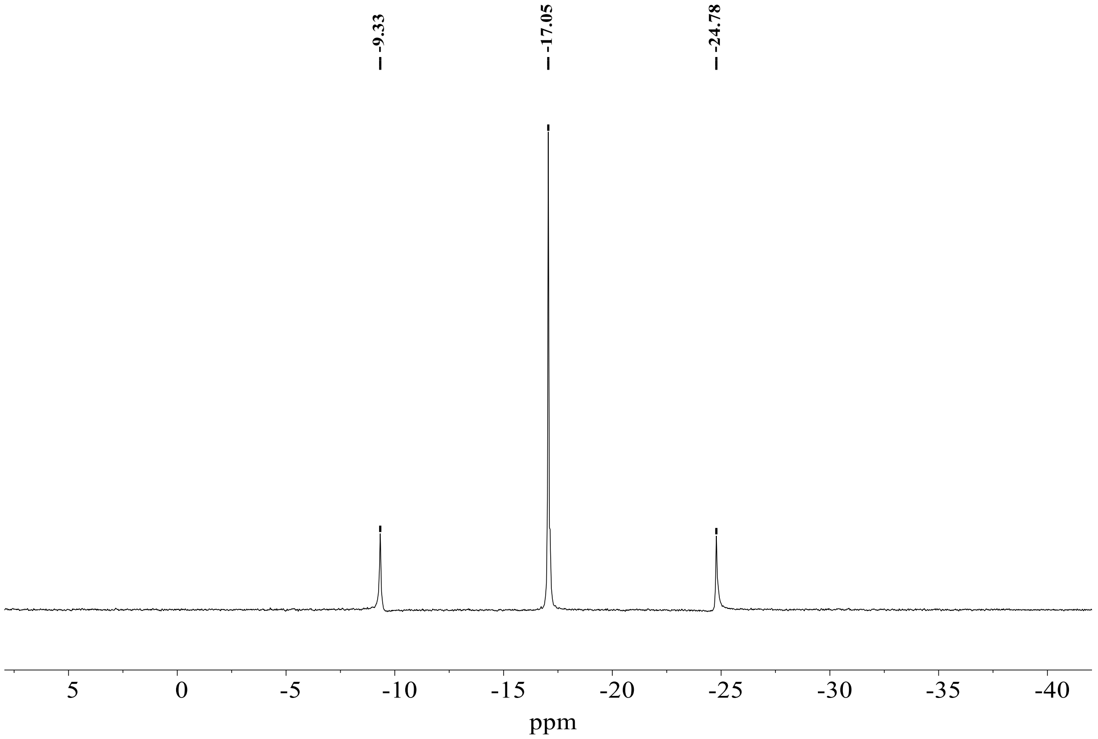

Figure S13.- a) ^{1}H and b) ^{31}P NMR (CD_2Cl_2) spectra of [Pt(dppp)(**iso-Ph**)$_2$](OTf)$_2$.

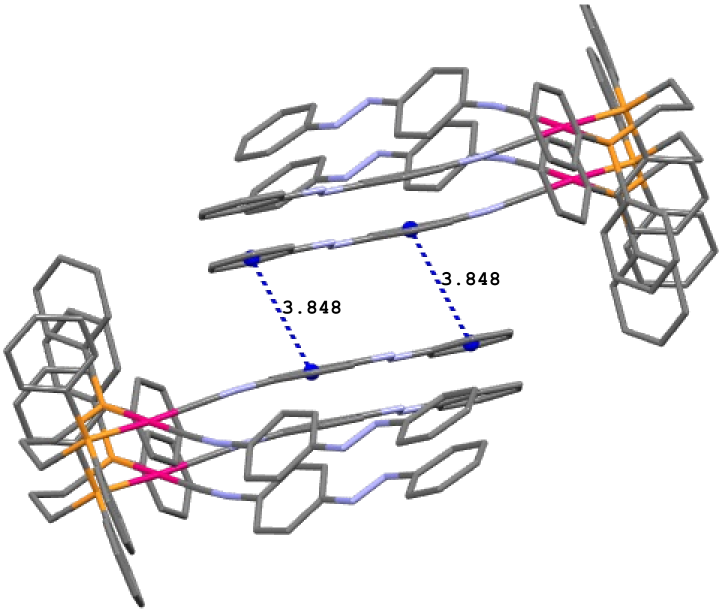

Figure S14.- View of the π-π stacking interactions in [Pd(dppp)(**iso-Ph**)$_2$](OTf)$_2$.

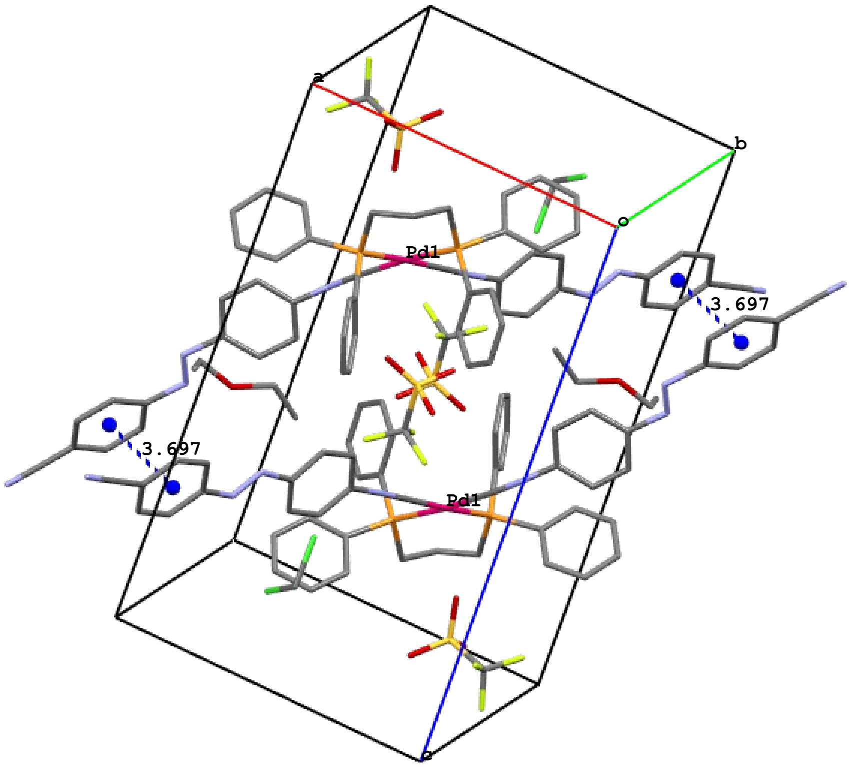

Figure S15.- View of the π-π stacking interactions of [Pd(dppp)(**iso-cyano**)$_2$](OTf)$_2$.

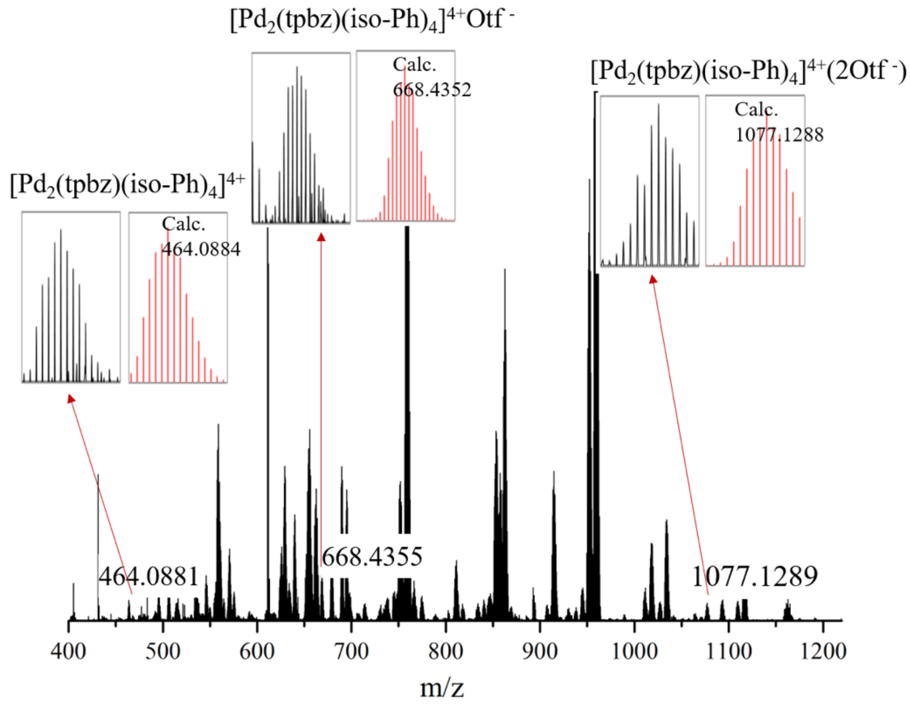

Figure S16.- ESI(+)-HRMS spectrum of [{Pd_2(tpbz)}(**iso-Ph**)$_4$]($OTf)_4$.

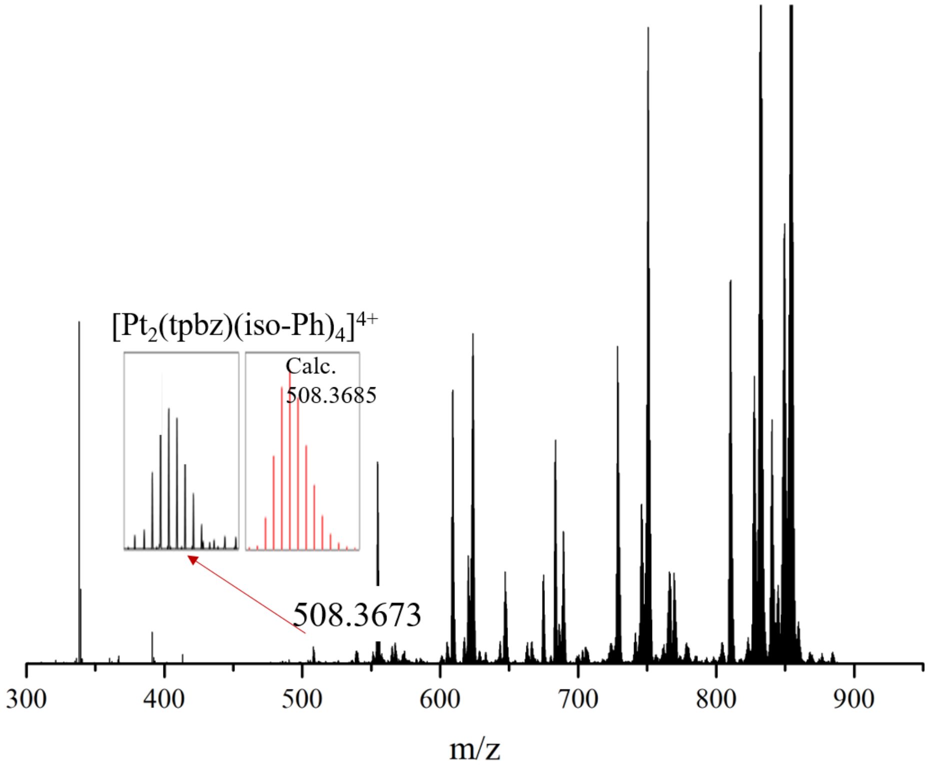

Figure S17.- ESI(+)-HRMS spectrum of [{Pt_2(tpbz)}(**iso-Ph**)$_4$]($OTf)_4$.

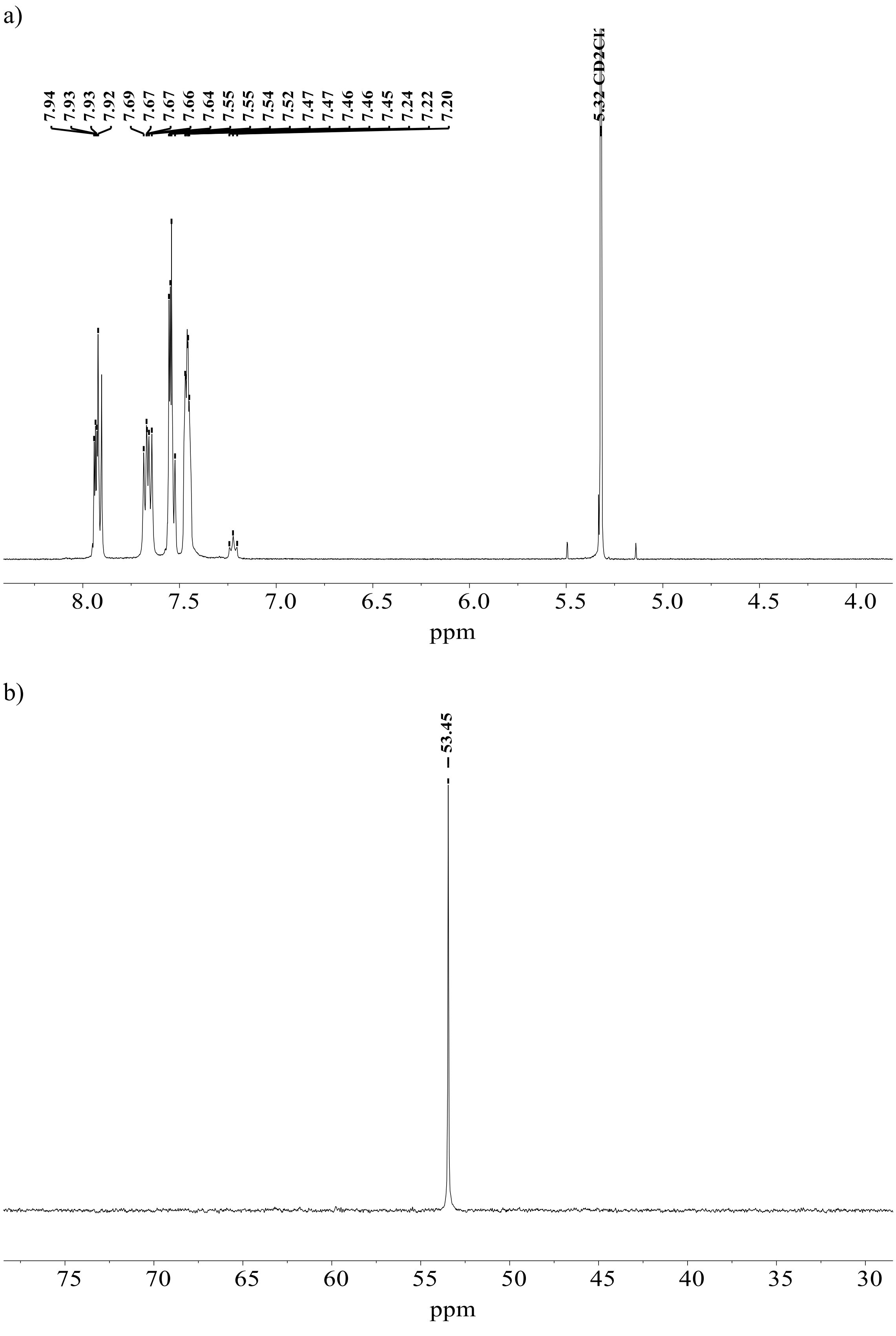

Figure S18.-a) ^{1}H and b) ^{31}P NMR (CD_2Cl_2) spectra of [{Pd_2(tpbz)}(**iso-Ph**)$_4$](OTf)$_4$.

a)

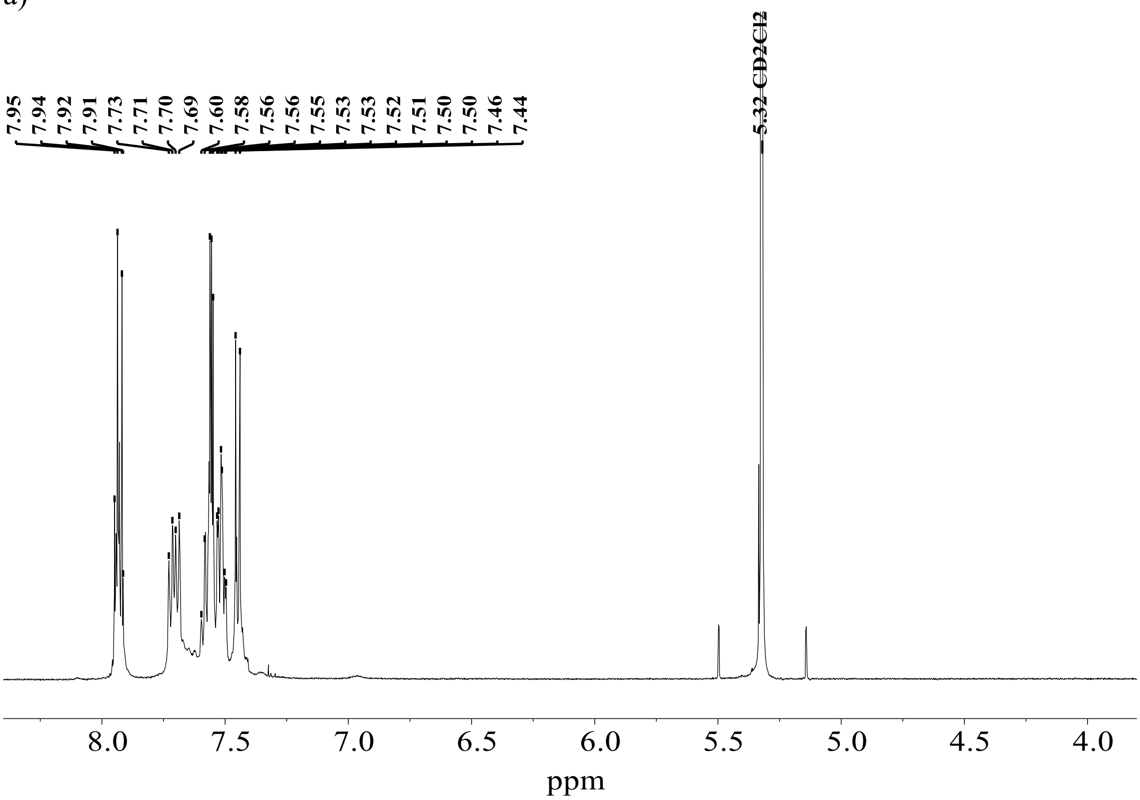

b)

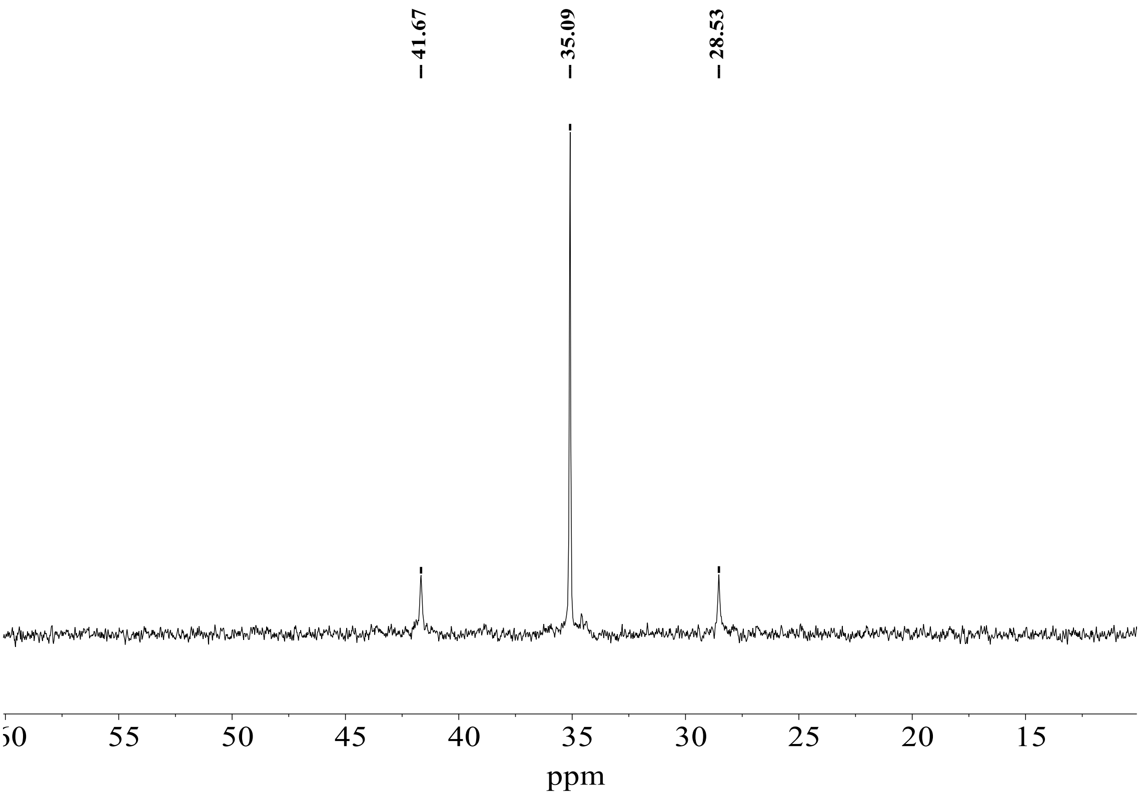

Figure S19.-a) ^{1}H and b) ^{31}P NMR (CD_2Cl_2) spectra of [{Pt_2(tpbz)}(**iso-Ph**)$_4$](OTf)$_4$.

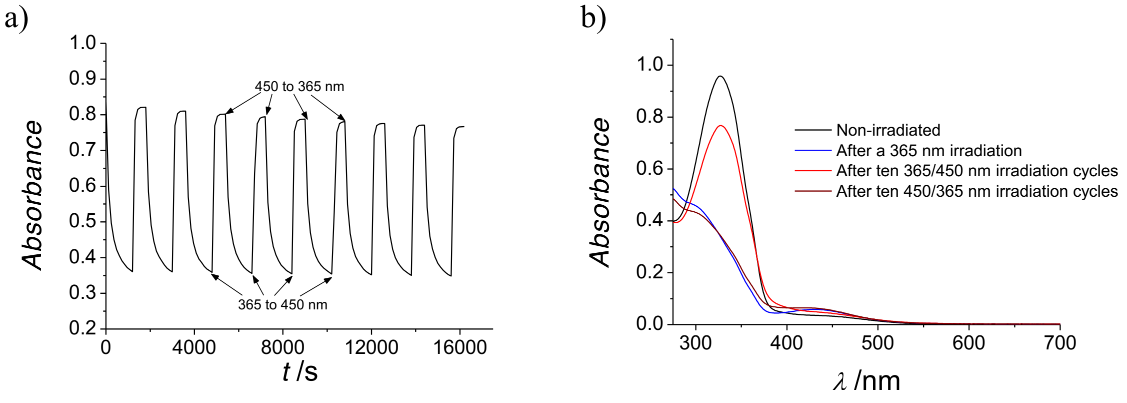

Figure S20.- a) Changes in the intensity of the UV-Vis signal at 340 nm of a acetonitrile solution of $[Pd(dppp)_2($**iso-Ph**$)_2]^{2+}$ on consecutive irradiation cycles at 365 and 450 nm. b) Initial and final spectrum (after 10 illumination cycles) of the same solution.

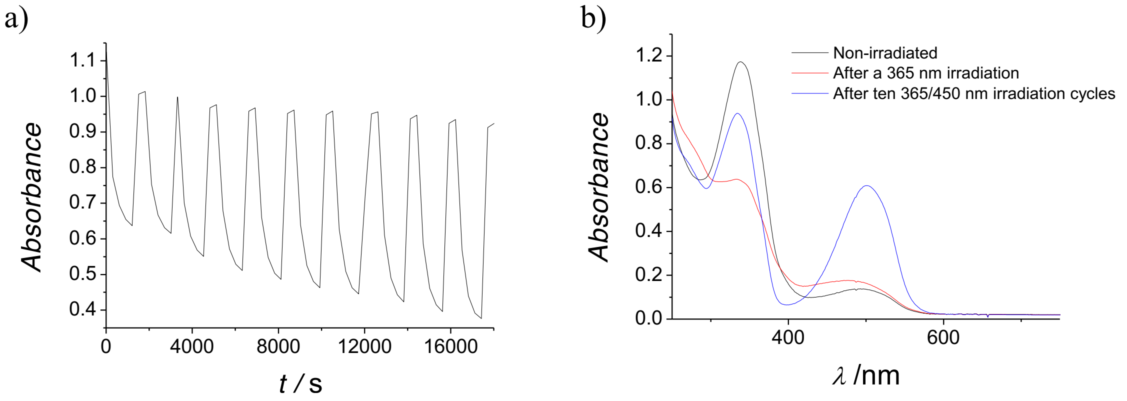

Figure S21.- a) Changes in the intensity of the UV-Vis signal at 340 nm of a dichloromethane solution of $[\{Pt_2(tpbz)\}($**iso-Ph**$)_4]^{4+}$ on consecutive irradiation cycles at 365 and 450 nm. b) Initial and final spectrum (after 10 illumination cycles) of the same solution.

a)

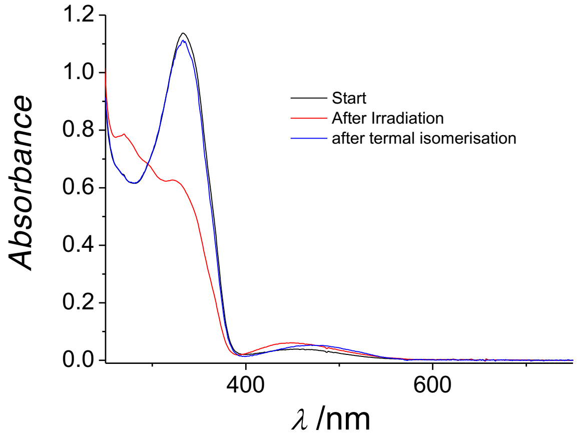

b)

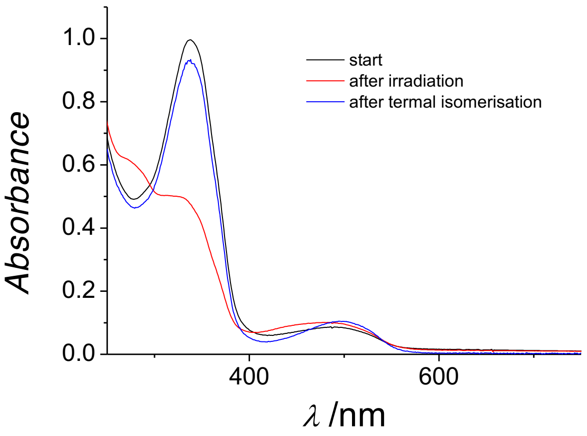

Figure S22.- Changes of the UV-Vis spectrum of a freshly prepared dichloromethane solution of a) [[{Pd_2(tpbz)}(**iso-Ph**)$_4$]$^{4+}$ and b) [{Pt_2(tpbz)}(**iso-Ph**)$_4$]$^{4+}$ after irradiation at 365 and spontaneous thermal recovery.

a)

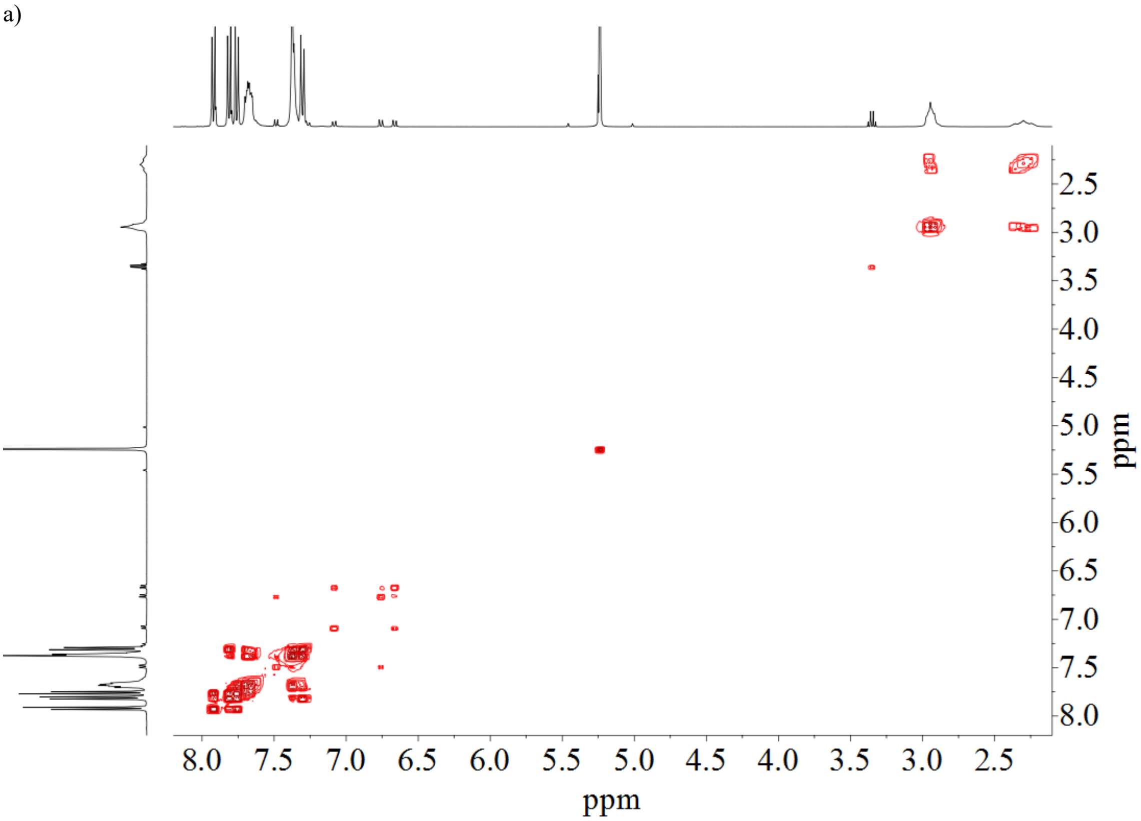

b)

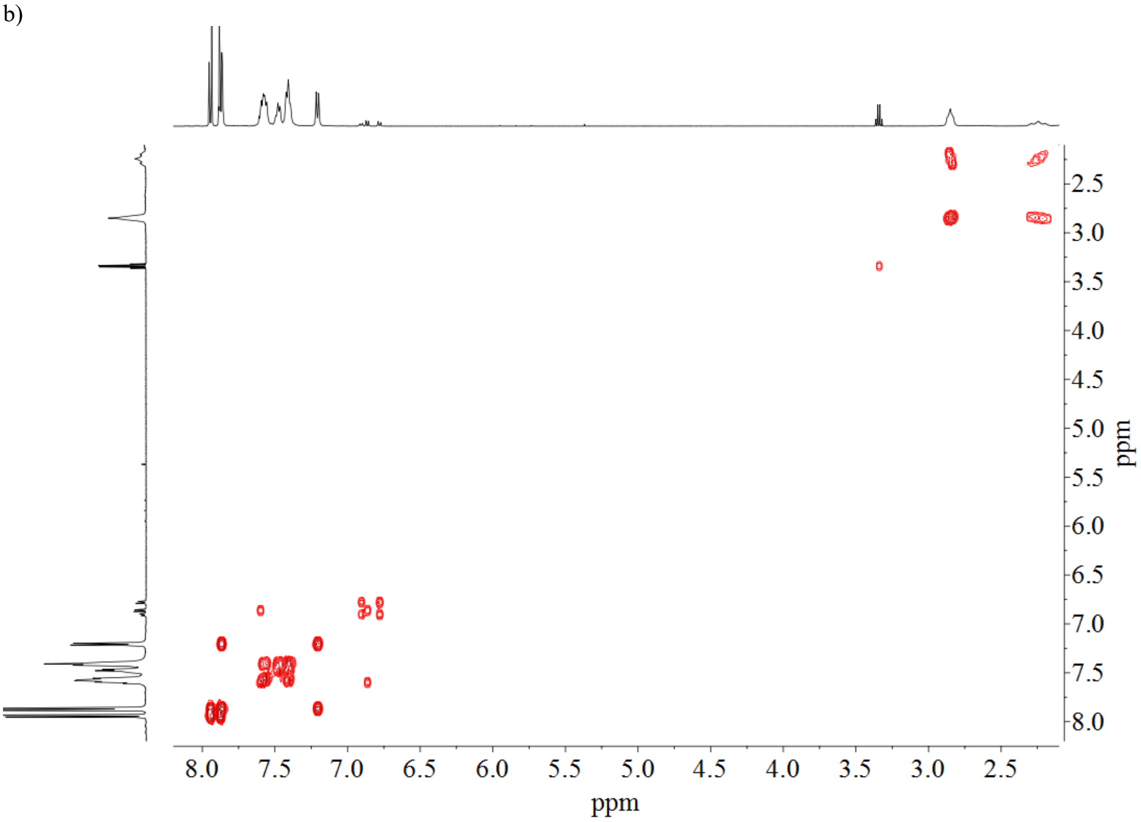

Figure S23.- COSY ^{1}H-^{1}H NMR spectra of $[Pd(dppp)($**iso-cyano**$)_2](OTf)_2$ a) in CD_2Cl_2 and b) CD_3CN after irradiation at 365 nm.

a)

a)

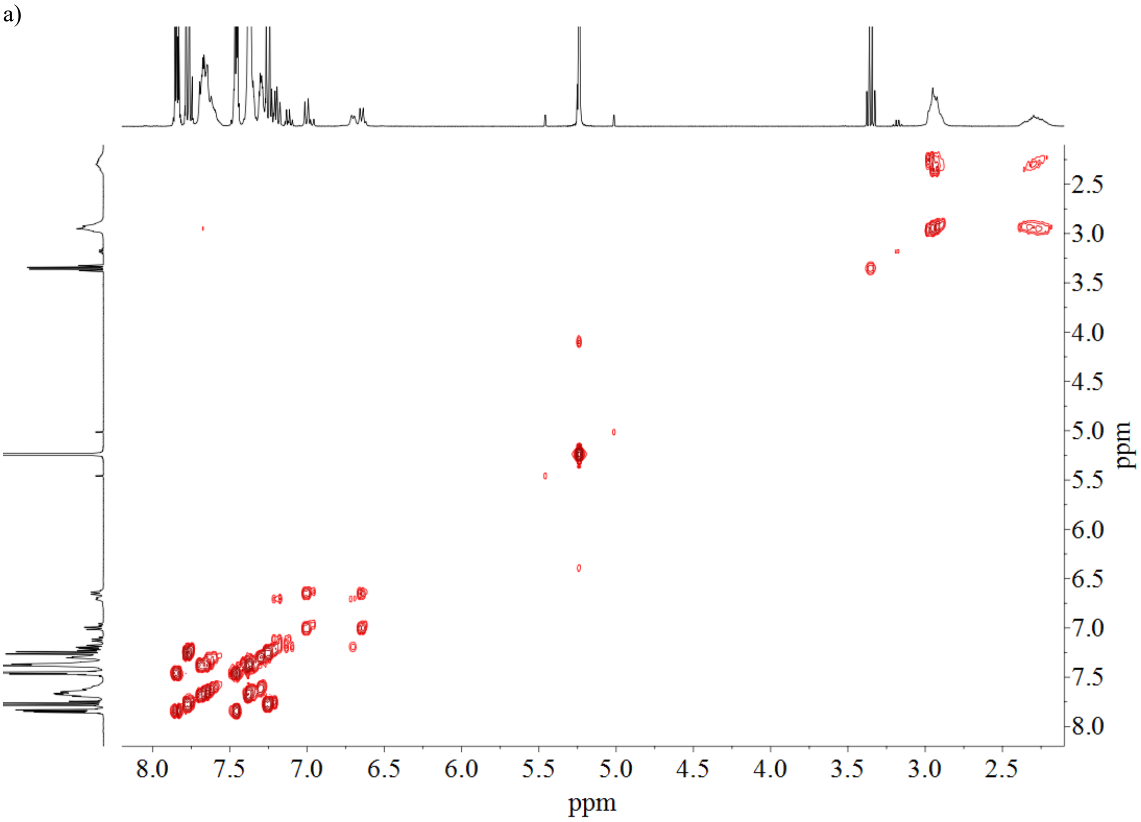

b)

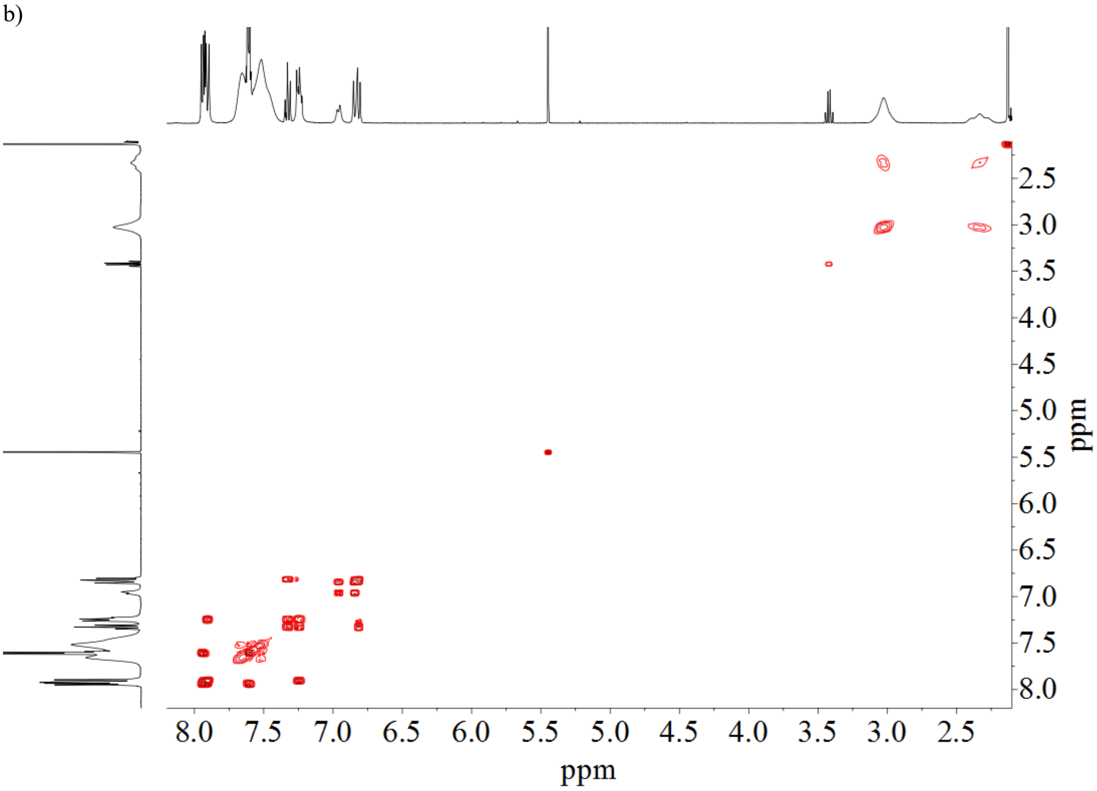

Figure S24.- COSY ^{1}H-^{1}H NMR spectra of [Pt(dppp)(**iso-Ph**)$_2$](OTf)$_2$ a) in CD_2Cl_2 and b) CD_3CN after irradiation at 365 nm.

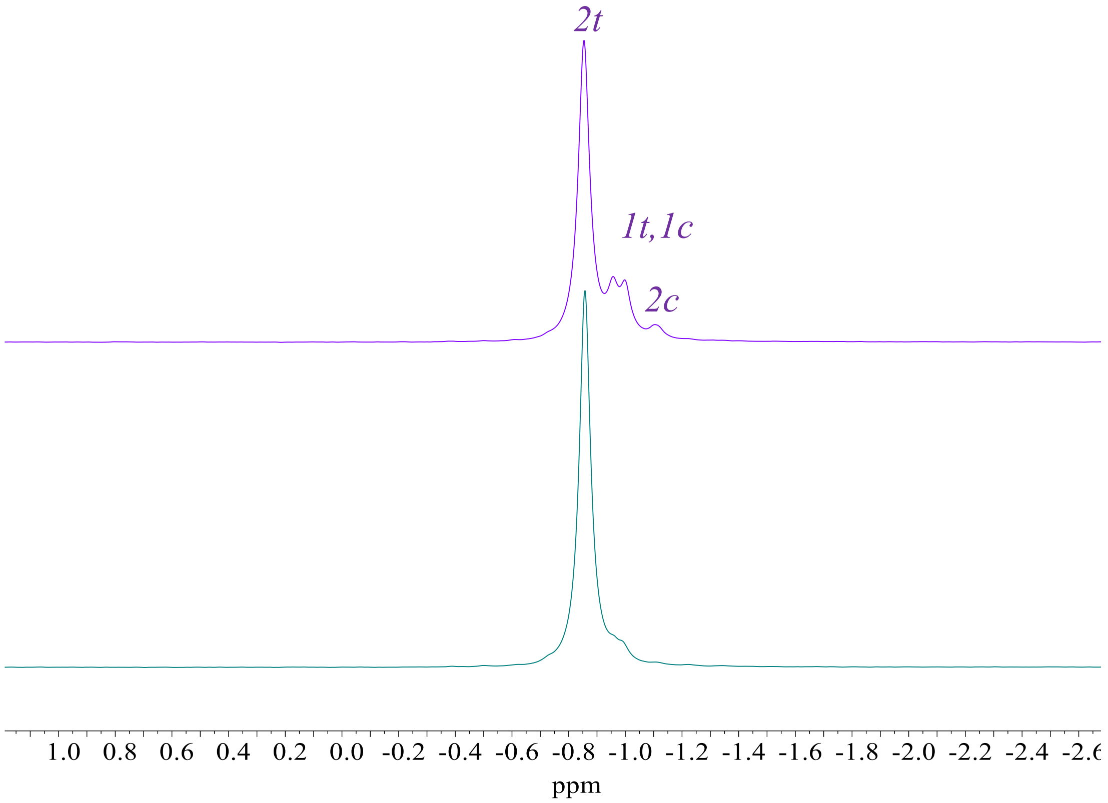

Figure S25.- ^{31}P NMR monitoring of a sample of illuminated $[Pd(dppp)_2($*trans*-**iso-Ph**$)_2]^{2+}$ where the major *trans-trans* (*2t*), the unsymmetrical *trans-cis* (*1t,1c*) and the and minor $[Pd(dppp)_2($*cis*-**iso-Ph**$)_2]^{2+}$ (*2c*) complex are observed.

a)

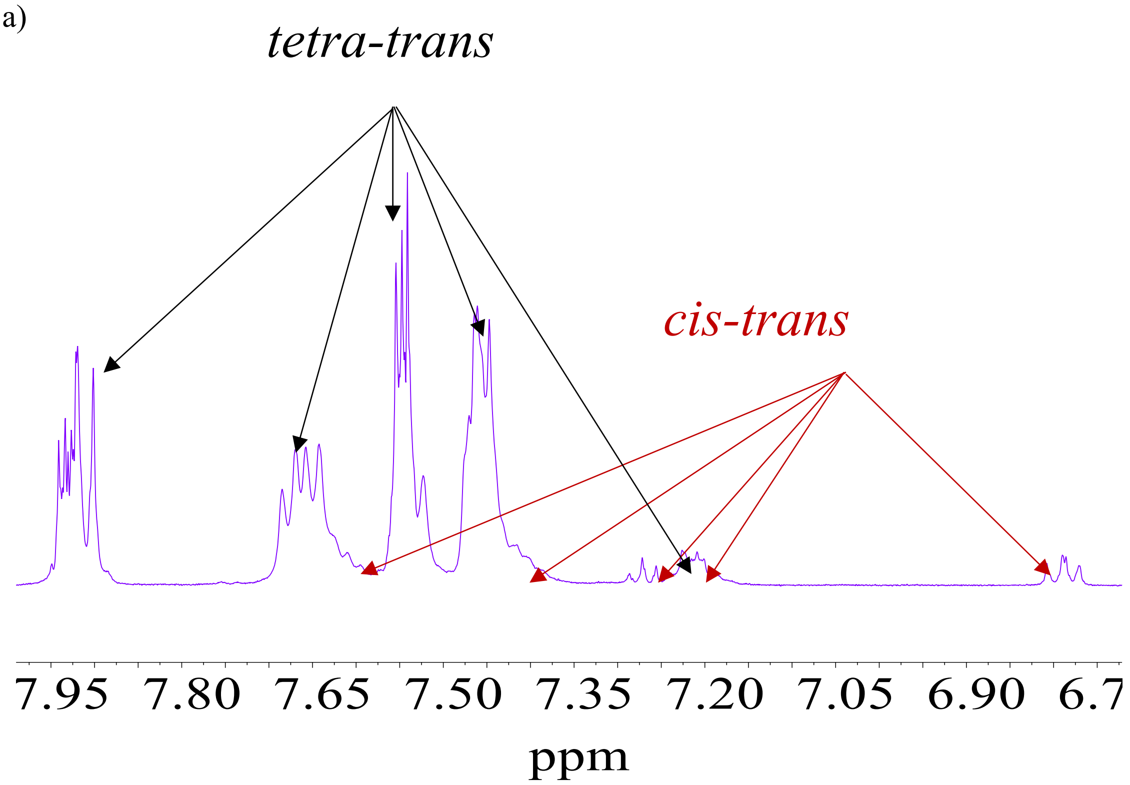

b)

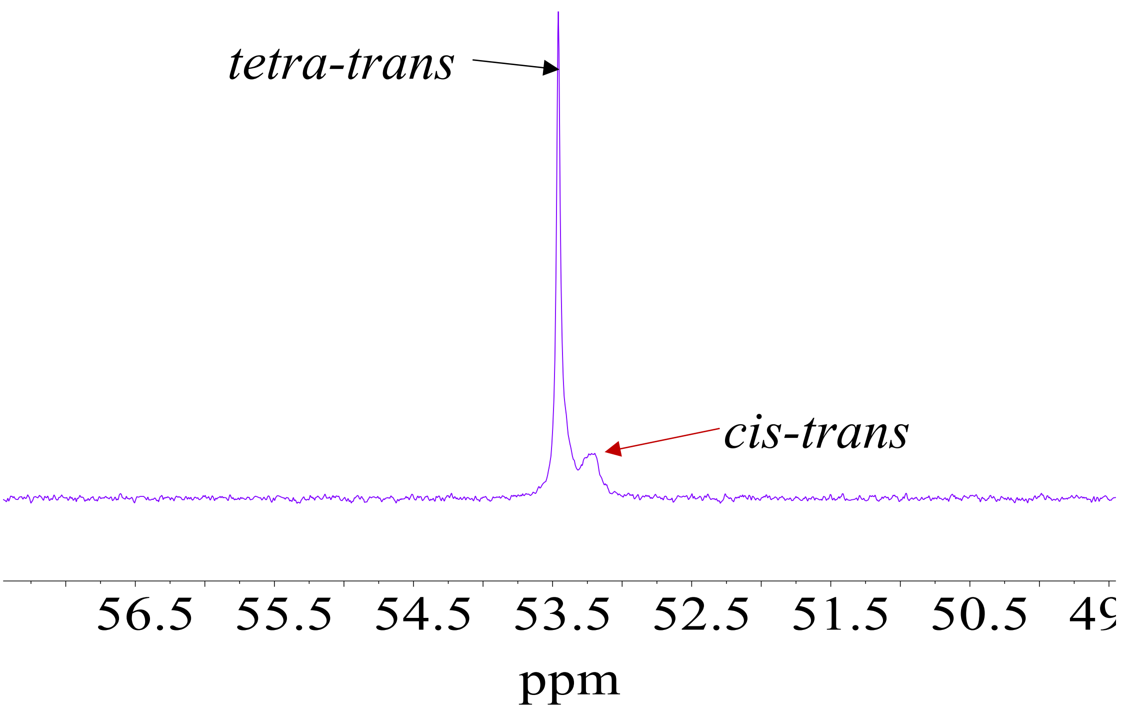

Figure S26.- a) ^{1}H and b) ^{31}P NMR of a sample of illuminated [[{Pd_2(tpbz)}(**iso-Ph**)$_4$]$^{4+}$ where the major *tetra-trans* and the unsymmetrical *trans-cis* complex mixtures are observed.

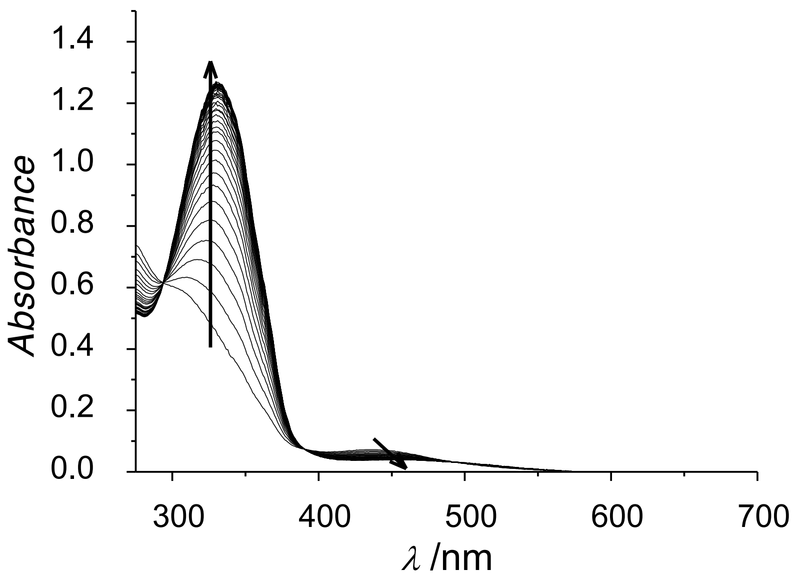

Figure S27.- Time-resolved UV-Vis monitoring of the spontaneous *cis*-to-*trans* reaction of $[Pd(dppp)_2($**iso-Ph**$)_2]^{2+}$ in CH_2Cl_2 solution at 55 ºC,

Table S1.- Crystal data and structure refinement for the [Pd(dppp)(**iso-Ph**)$_2$](OTf)$_2$ and [Pd(dppp)(**iso-cyano**)$_2$](OTf)$_2$ complexes.

**Empirical formula**	$C_{55}H_{44}F_6N_6O_6P_2PdS_2$	$C_{62}H_{54}Cl_2F_6N_8O_7P_2PdS_2$
**Compound**	[Pd(dppp)(**iso-Ph**)$_2$](TfO)$_2$	[Pd(dppp)(**iso-cyano**)$_2$](TfO)$_2$
Formula weight	1231.42 g/mol	1440.49 g/mol
Temperature	320(2) K	100(2) K
Wavelength	0.71073 Å	0.71073 Å
Crystal system	triclinic	triclinic
Space group	P -1	P -1
Unit cell dimensions	a = 11.2905(5) Å	a = 11.2047(7) Å
	b = 13.5185(7) Å	b = 16.6352(10) Å
	c = 20.7490(10) Å	c = 18.7949(12) Å
	α = 88.726(2)°	α = 103.902(2)°
	β = 84.534(2)°	β = 94.921(2)°
	γ = 66.460(2)°	γ = 104.812(2)°
Volume	2889.7(2) Å^3	3246.9(4) Å^3
Z	2	2
Density (calculated)	1.415 g/cm^3	1.473 g/cm^3
Absorption coefficient	0.521 mm^{-1}	0.557 mm^{-1}
F(000)	1252	1468
Crystal size	0.467 mm x 0.127 mm x 0.069 mm	0.196 mm x 0.169 mm x 0.094 mm
Theta range for data collection	1.90 to 30.77°	1.90 to 26.56°
Index ranges	-16<=h<=16, -19<=k<=19, -29<=l<=29	-14<=h<=14, -20<=k<=20, -23<=l<=23
Reflections collected	146224	139213
Independent reflections	17917 [R(int) = 0.0941]	13424 [R(int) = 0.0970]
Completeness to theta = 25.242°	100%	100%
Absorption correction	multi-scan method (SADABS)	multi-scan method (SADABS)
Max. and min. transmission	0.7461 and 0.5700	0.7454 and 0.5839
Refinement method	Full-matrix least-squares on F2	Full-matrix least-squares on F2
Data / restraints / parameters	17917 / 7 / 665	13424 / 15 / 753
Goodness-of-fit on F^2	1.025	1.089
Final R indices [I>2sigma(I)]	R1 = 0.0705, wR2 = 0.1864	R1 = 0.1147, wR2 = 0.2965
R indices (all data)	R1 = 0.1236, wR2 = 0.2255	R1 = 0.1275, wR2 = 0.3064
Extinction coefficient	n/a	n/a
Largest diff. peak and hole	1.267 and -1.055 eÅ^{-3}	6.940 and -3.397 eÅ^{-3}
CCDC deposition number	2343066	2343065

Table S2.- UV-Vis spectral data, λ_{max} (ε), for the free ligands and the complexes studied in this work.

Compound	Solvent	π-π* /nm($10^3M^{-1}cm^{-1}$)	nb-π*/nm($10^3M^{-1}cm^{-1}$)
**iso-cyano**	Toluene§	333 (22)	450 (0.55)
	Dichloromethane	328 (29)	458 (0.69)
	Acetonitrile	326 (32)	451 (0.77)
	Methanol§	326 (24)	450 (0.40)
**iso-Ph**	Dichloromethane	326 (23)	450 (0.66)
	Acetonitrile	322 (17)	450 (0.35)
$[Pd(dppp)($**iso-cyano**$)_2]^{2+}$	Dichloromethane	332 (73)	470 (1.2)
	Acetonitrile	330 (79)	458 (1.7)
$[Pt(dppp)($**iso-cyano**$)_2]^{2+}$	Dichloromethane	332 (79)	462 (1.8)
	Acetonitrile	330 (85)	451 (2.2)
$[Pd(dppp)($**iso-Ph**$)_2]^{2+}$	Dichloromethane	330 (59)	432 (2.3)
	Acetonitrile	326 (65)	433 (1.6)
$[Pt(dppp)($**iso-Ph**$)_2]^{2+}$	Dichloromethane	333 (59)	466 (1.3)
	Acetonitrile	334 (68)	457 (2.0)
$[\{Pd_2(tpbz)\}($**iso-Ph**$)_4]^{4+}$	Dichloromethane	335 (130)	460 (4.9)
$[\{Pt_2(tpbz)\}($**iso-Ph**$)_4]^{4+}$	Dichloromethane	336 (116)	485 (9.1)

§ Martínez, M.; Ferrer, M.; Garcia-Cirera, B.; Gallen, A.; Font-Bardia, M.; Bardaji, M., Iron complexes of bridging azo ligands in aqueous solution; changes in the thermal switching mechanism on coordination and oxidation state of metal centres. *Dalton Transactions* **2023,** *52*, 1720-1730.

Table S3.- Values of the observed 1st-order rate constants for the spontaneous *cis*-to-*trans* isomerisation reaction occurring on the compounds prepared as a function of solvent, temperature and pressure ([compounds] at the 10 μM level).

Compound	Solvent	T /ºC	P /atm.	$10^3\times k_{obs}$ /s^{-1}
**iso-Ph**	Dichloromethane	35	1	0.010
**iso-Ph**	Dichloromethane	45	1	0.028
**iso-Ph**	Dichloromethane	55	1	0.087
**iso-Ph**	Acetonitrile	35	1	0.0060
**iso-Ph**	Acetonitrile	45	1	0.016
**iso-Ph**	Acetonitrile	55	1	0.058
**iso-Ph**	Acetonitrile	63	400	0.16
**iso-Ph**	Acetonitrile	63	700	0.15
**iso-Ph**	Acetonitrile	63	1000	0.15
**iso-Ph**	Acetonitrile	63	1300	0.14
**iso-Ph**	Acetonitrile	63	1600	0.13
**iso-Ph**	Methanol	35	1	0.0070
**iso-Ph**	Methanol	45	1	0.020
**iso-Ph**	Methanol	55	1	0.063
**iso-Ph**	Toluene	35	1	0.020
**iso-Ph**	Toluene	35	1	0.015
**iso-Ph**	Toluene	45	1	0.047
**iso-Ph**	Toluene	55	1	0.13
**iso-Ph**	Toluene	60	1	0.26
**iso-Ph**	Toluene	63	400	0.38
**iso-Ph**	Toluene	63	700	0.37
**iso-Ph**	Toluene	63	1000	0.35
**iso-Ph**	Toluene	63	1300	0.33
**iso-Ph**	Toluene	63	1600	0.32
**iso-cyano**	Dichloromethane	35	1	0.023
**iso-cyano**	Dichloromethane	45	1	0.072
**iso-cyano**	Dichloromethane	55	1	0.21
**iso-cyano**	Acetonitrile	35	1	0.013
**iso-cyano**	Acetonitrile	45	1	0.037
**iso-cyano**	Acetonitrile	55	1	0.12
**iso-cyano**	Acetonitrile	63	400	0.37
**iso-cyano**	Acetonitrile	63	1000	0.35
**iso-cyano**	Acetonitrile	63	1600	0.37
[Pd(dppp)(**iso-Ph**)$_2$]$^{2+}$	Dichloromethane	35	1	0.047
[Pd(dppp)(**iso-Ph**)$_2$]$^{2+}$	Dichloromethane	45	1	0.16
[Pd(dppp)(**iso-Ph**)$_2$]$^{2+}$	Dichloromethane	55	1	0.49
[Pd(dppp)(**iso-Ph**)$_2$]$^{2+}$	Acetonitrile	35	1	0.033
[Pd(dppp)(**iso-Ph**)$_2$]$^{2+}$	Acetonitrile	45	1	0.10
[Pd(dppp)(**iso-Ph**)$_2$]$^{2+}$	Acetonitrile	55	1	0.36
[Pt(dppp)(**iso-Ph**)$_2$]$^{2+}$	Dichloromethane	35	1	0.062
[Pt(dppp)(**iso-Ph**)$_2$]$^{2+}$	Dichloromethane	45	1	0.26
[Pt(dppp)(**iso-Ph**)$_2$]$^{2+}$	Dichloromethane	55	1	0.64
[Pt(dppp)(**iso-Ph**)$_2$]$^{2+}$	Acetonitrile	35	1	0.042
[Pt(dppp)(**iso-Ph**)$_2$]$^{2+}$	Acetonitrile	45	1	0.16
[Pt(dppp)(**iso-Ph**)$_2$]$^{2+}$	Acetonitrile	55	1	0.42
[Pt(dppp)(**iso-Ph**)$_2$]$^{2+}$	Acetonitrile	60	400	0.39
[Pt(dppp)(**iso-Ph**)$_2$]$^{2+}$	Acetonitrile	60	700	0.29
[Pt(dppp)(**iso-Ph**)$_2$]$^{2+}$	Acetonitrile	60	1000	0.24
[Pt(dppp)(**iso-Ph**)$_2$]$^{2+}$	Acetonitrile	60	1300	0.17
[Pt(dppp)(**iso-Ph**)$_2$]$^{2+}$	Acetonitrile	60	1600	0.13
[Pt(dppp)(**iso-Ph**)$_2$]$^{2+}$	Methanol	35	1	0.030
[Pt(dppp)(**iso-Ph**)$_2$]$^{2+}$	Methanol	45	1	0.084
[Pt(dppp)(**iso-Ph**)$_2$]$^{2+}$	Methanol	55	1	0.30
[Pd(dppp)(**iso-cyano**)$_2$]$^{2+}$	Dichloromethane	35	1	0.026
[Pd(dppp)(**iso-cyano**)$_2$]$^{2+}$	Dichloromethane	45	1	0.081
[Pd(dppp)(**iso-cyano**)$_2$]$^{2+}$	Dichloromethane	55	1	0.22

	Acetonitrile	35	1	0.014
		45	1	0.051
		55	1	0.16
[Pt(dppp)(**iso-cyano**)$_2$]$^{2+}$	Dichloromethane	35	1	0.036
		45	1	0.10
		55	1	0.27
	Acetonitrile	35	1	0.014
		35	1	0.015
		45	1	0.051
		50	1	0.078
		55	1	0.19
		55	1	0.20
[[{Pd$_2$(tpbz)}(**iso-Ph**)$_4$]$^{4+}$	Dichloromethane	25	1	0.043
		35	1	0.12
		45	1	0.35
		55	1	0.73
[[{Pt$_2$(tpbz)}(**iso-Ph**)$_4$]$^{4+}$	Dichloromethane	25	1	0.023
		35	1	0.082
		45	1	0.23
		55	1	0.68

Table S3.- Computed DFT absolute Gibbs energies (at 298.15K) and cartesian (xyz) coordinates for the computed structures indicated.

[Pd(dppp)(*trans*-**iso-Ph**)$_2$]$^{2+}$

G=-3184.138185 Ha

Pd	-0.002580	-0.391426	1.857178
P	1.793568	-0.308991	3.398831
P	-1.601992	-0.574601	3.594755
C	1.539779	-1.274118	4.966836
H	1.567473	-2.340553	4.673269
H	2.443795	-1.108962	5.580938
C	0.264833	-0.962369	5.770066
H	0.360319	-1.456650	6.754361
H	0.185834	0.121046	5.976693
C	-1.021982	-1.485329	5.108146
H	-1.865748	-1.468387	5.821697
H	-0.906343	-2.542779	4.803883
C	2.135175	1.426141	3.869736
C	1.889284	2.454980	2.931075
H	1.460610	2.212060	1.951968
C	2.194752	3.786711	3.246882
H	2.005522	4.576434	2.512444
C	2.738939	4.105718	4.502245
H	2.974370	5.146062	4.749091
C	2.981721	3.090025	5.441655
H	3.407127	3.334820	6.420294
C	2.684420	1.753954	5.131193
H	2.892799	0.978703	5.875403
C	-2.166688	1.078232	4.138812
C	-2.102416	2.164212	3.235876
H	-1.680456	2.018996	2.234890
C	-2.577317	3.427875	3.616273
H	-2.526771	4.262660	2.909625
C	-3.112859	3.621514	4.900337
H	-3.480954	4.608915	5.197368
C	-3.176820	2.548327	5.804752
H	-3.594903	2.695522	6.805835
C	-2.708166	1.279772	5.430326
H	-2.778623	0.456074	6.148147
C	3.342725	-1.046800	2.742841
C	4.581050	-0.384759	2.879897
H	4.629890	0.614606	3.324305
C	5.759837	-1.017860	2.449300
H	6.720282	-0.504436	2.563777
C	5.708355	-2.303016	1.884875
H	6.630192	-2.794450	1.556974
C	4.473682	-2.960824	1.740878
H	4.430891	-3.961331	1.298203
C	3.292015	-2.335899	2.164634
H	2.331755	-2.852491	2.040667
C	-3.069043	-1.560871	3.093261
C	-2.863798	-2.831196	2.507138
H	-1.848186	-3.186685	2.291288
C	-3.962393	-3.644566	2.193926
H	-3.798973	-4.630200	1.745949
C	-5.268935	-3.194068	2.456393
H	-6.125260	-3.833039	2.216865
C	-5.475270	-1.927159	3.026990
H	-6.491379	-1.574110	3.231859
C	-4.379944	-1.106449	3.347115
H	-4.549169	-0.122164	3.795462
C	1.315325	-0.233895	0.355175
N	2.048503	-0.152790	-0.567526
C	2.939886	-0.066950	-1.617952
C	2.449148	0.003879	-2.943192
C	4.332411	-0.051918	-1.344227
C	3.361600	0.092031	-3.992170
H	1.371484	-0.013898	-3.129753
C	5.228096	0.038366	-2.403257
H	4.680407	-0.115041	-0.308251
H	3.024319	0.147280	-5.031556
C	4.755158	0.112618	-3.740717
H	6.308500	0.054290	-2.235577
N	5.576655	0.205792	-4.887402
N	6.832052	0.232733	-4.605332
C	7.688138	0.322330	-5.713791
C	7.262513	0.376818	-7.068618
C	9.070036	0.357281	-5.399789
C	8.216439	0.464612	-8.081133
H	6.191680	0.348285	-7.287679
C	10.018675	0.445828	-6.423259
H	9.362477	0.313709	-4.345770
C	9.592688	0.499363	-7.762713
H	7.900269	0.506865	-9.128727
H	11.086357	0.473282	-6.183895
H	10.332936	0.568504	-8.567024
C	-1.506664	-0.364909	0.530475
N	-2.357697	-0.318486	-0.287276
C	-3.377177	-0.276290	-1.217540
C	-4.648631	-0.794084	-0.874246
C	-3.129835	0.287474	-2.495910
C	-5.667945	-0.743600	-1.822956
H	-4.813421	-1.225654	0.117384
C	-4.159381	0.331949	-3.428437
H	-2.137061	0.684523	-2.730516
H	-6.665568	-1.134451	-1.600952
C	-5.442725	-0.183343	-3.103814
H	-4.009775	0.760902	-4.423026
N	-6.556943	-0.190818	-3.973980
N	-6.305965	0.346926	-5.115875
C	-7.382062	0.358995	-6.016672
C	-8.673056	-0.171670	-5.749631
C	-7.103825	0.957404	-7.271206
C	-9.658823	-0.097041	-6.732258
H	-8.865339	-0.628709	-4.775136
C	-8.100156	1.027322	-8.249879
H	-6.098855	1.356048	-7.442907
C	-9.376414	0.500726	-7.981083
H	-10.657233	-0.502761	-6.537870
H	-7.888420	1.489065	-9.219392
H	-10.159205	0.553660	-8.745353

[Pd(dppp)(*trans*-**iso-Ph**)(*cis*-**iso-Ph**)]$^{2+}$

G = -3184.114803 Ha

Pd	-0.019590	-0.341646	1.784285
P	1.732613	-0.359799	3.375647
P	-1.627675	-0.954216	3.411688

C	1.537530	-1.581831	4.761820
H	1.673311	-2.579014	4.301984
H	2.409031	-1.437556	5.426289
C	0.221155	-1.525067	5.557234
H	0.344174	-2.161650	6.452725
H	0.029760	-0.502338	5.931525
C	-0.994315	-2.050554	4.773981
H	-1.849350	-2.225582	5.451899
H	-0.770752	-3.028783	4.308131
C	1.913868	1.301819	4.122070
C	1.689475	2.442760	3.316508
H	1.372799	2.324117	2.273936
C	1.877439	3.727929	3.844826
H	1.707668	4.605459	3.212319
C	2.280296	3.888243	5.181415
H	2.423879	4.892206	5.593848
C	2.500279	2.760343	5.988806
H	2.816426	2.881196	7.029988
C	2.321074	1.470099	5.465690
H	2.509822	0.606819	6.111497
C	-2.353198	0.522554	4.210621
C	-2.263998	1.777768	3.567291
H	-1.731824	1.865454	2.613090
C	-2.851407	2.911906	4.147513
H	-2.779374	3.880267	3.641760
C	-3.526584	2.805020	5.374352
H	-3.982969	3.691017	5.827594
C	-3.617147	1.561134	6.021852
H	-4.143858	1.474671	6.977748
C	-3.035409	0.421513	5.446484
H	-3.128749	-0.539809	5.962636
C	3.356488	-0.848260	2.667927
C	4.530333	-0.129344	2.977628
H	4.480533	0.776900	3.589888
C	5.771984	-0.585715	2.502528
H	6.681946	-0.029290	2.750546
C	5.847042	-1.752804	1.724418
H	6.817855	-2.109144	1.364559
C	4.676777	-2.466390	1.409285
H	4.732682	-3.375442	0.801465
C	3.432978	-2.015877	1.874903
H	2.523310	-2.572939	1.617339
C	-2.985239	-1.967443	2.698910
C	-2.647307	-3.124763	1.959794
H	-1.596339	-3.373827	1.765468
C	-3.658191	-3.961838	1.466053
H	-3.391728	-4.860893	0.900727
C	-5.009216	-3.646851	1.699481
H	-5.797104	-4.305438	1.319401
C	-5.347197	-2.490353	2.421394
H	-6.398016	-2.242224	2.603838
C	-4.340406	-1.647001	2.922246
H	-4.612873	-0.749585	3.486719
C	-1.498688	-0.196013	0.438170
N	-2.332342	-0.067908	-0.388578
C	-3.326646	0.065753	-1.337916
C	-3.135660	0.954921	-2.421648
C	-4.520620	-0.688703	-1.203233
C	-4.148442	1.084019	-3.369745
H	-2.209447	1.531850	-2.499835
C	-5.518640	-0.550069	-2.161264
H	-4.638267	-1.367852	-0.352906
H	-4.043249	1.764276	-4.220281
C	-5.346067	0.337069	-3.256826
H	-6.452703	-1.114824	-2.095952
N	-6.297207	0.558241	-4.279544
N	-7.352226	-0.166851	-4.150322
C	-8.330694	0.026549	-5.138241
C	-8.227748	0.946545	-6.216308
C	-9.487164	-0.781140	-4.999850
C	-9.273622	1.045044	-7.132573
H	-7.326826	1.559970	-6.303311
C	-10.530383	-0.674876	-5.925044
H	-9.533314	-1.479008	-4.157687
C	-10.424169	0.237297	-6.990373
H	-9.205584	1.751117	-7.966880
H	-11.424516	-1.297516	-5.820842
H	-11.239554	0.323586	-7.716577
C	1.307085	0.165702	0.371388
N	2.050221	0.477547	-0.494716
C	2.957324	0.841626	-1.463206
C	2.501309	1.195222	-2.758399
C	4.339224	0.883124	-1.142351
C	3.420579	1.579678	-3.728139
H	1.432181	1.156302	-2.988270
C	5.248150	1.296938	-2.108252
H	4.672546	0.604637	-0.138029
C	4.810083	1.630467	-3.419544
H	3.079730	1.863152	-4.728045
H	6.313440	1.376972	-1.871355
N	5.716987	2.289595	-4.270148
N	5.953431	2.099166	-5.490090
C	5.590135	0.918400	-6.194111
C	5.446793	-0.368009	-5.617303
C	5.498631	1.085131	-7.596931
C	5.175134	-1.461531	-6.445623
H	5.596395	-0.513521	-4.543621
C	5.183980	-0.008976	-8.409639
H	5.669711	2.082480	-8.014107
C	5.025905	-1.283271	-7.835483
H	5.093403	-2.464199	-6.012737
H	5.091125	0.122926	-9.492262
H	4.814841	-2.147312	-8.474318

[Pd(dppp)(*cis*-**iso-Ph**)$_2$]$^{2+}$
G = -3184.091785 Ha

Pd	0.231383	-0.728682	1.785438
P	1.693765	-0.958759	3.633478
P	-1.661292	-0.545859	3.195953
C	0.970667	-1.828401	5.109062
H	0.836031	-2.883265	4.803553
H	1.760859	-1.837895	5.881729
C	-0.340101	-1.251355	5.671662
H	-0.527016	-1.734521	6.648340
H	-0.235061	-0.170199	5.878444
C	-1.563164	-1.518403	4.777105
H	-2.502562	-1.318474	5.323896
H	-1.607768	-2.583632	4.481386
C	2.269243	0.684541	4.198168
C	2.363638	1.740381	3.262469

H	2.057484	1.578239	2.222483
C	2.848508	2.994897	3.660028
H	2.920638	3.806332	2.928383
C	3.235665	3.210284	4.993164
H	3.610290	4.191116	5.303572
C	3.141979	2.167259	5.929435
H	3.444197	2.331047	6.968902
C	2.662958	0.907385	5.538369
H	2.609198	0.106990	6.283299
C	-1.969026	1.200818	3.646914
C	-1.570277	2.223759	2.755960
H	-1.045913	1.967104	1.828348
C	-1.845384	3.566346	3.053809
H	-1.537363	4.351887	2.356030
C	-2.511435	3.901421	4.244417
H	-2.723306	4.949998	4.477430
C	-2.906810	2.891196	5.136919
H	-3.427476	3.148647	6.064966
C	-2.640802	1.544899	4.843386
H	-2.969470	0.774623	5.548449
C	3.167523	-1.994035	3.267831
C	4.462912	-1.579766	3.642834
H	4.620077	-0.598671	4.102590
C	5.556437	-2.437636	3.432517
H	6.559318	-2.116412	3.733042
C	5.363800	-3.701995	2.851821
H	6.217577	-4.369991	2.697694
C	4.074143	-4.112748	2.469695
H	3.921916	-5.096537	2.013825
C	2.977781	-3.262260	2.672684
H	1.976106	-3.586591	2.363445
C	-3.189044	-1.223555	2.430761
C	-3.154052	-2.526078	1.881997
H	-2.213004	-3.089432	1.846308
C	-4.326651	-3.105344	1.375899
H	-4.296778	-4.117656	0.959605
C	-5.536167	-2.387770	1.406444
H	-6.452297	-2.844459	1.017623
C	-5.570098	-1.088266	1.938715
H	-6.510316	-0.527481	1.964142
C	-4.400529	-0.501688	2.452322
H	-4.436625	0.509016	2.871359
C	1.807941	-0.856373	0.557493
N	2.697308	-0.968764	-0.214307
C	3.746405	-1.129616	-1.090280
C	5.042853	-1.406465	-0.586750
C	3.512709	-1.043964	-2.486936
C	6.097996	-1.582460	-1.475539
H	5.201419	-1.478011	0.493754
C	4.565647	-1.252773	-3.368961
H	2.504874	-0.836214	-2.858371
C	5.877566	-1.501818	-2.880208
H	7.101319	-1.804791	-1.100809
H	4.398292	-1.234094	-4.450107
N	6.844758	-1.966181	-3.791719
N	8.046673	-1.623185	-3.926336
C	8.593134	-0.421677	-3.396846
C	7.869202	0.773814	-3.163222
C	9.998513	-0.448551	-3.229179
C	8.554082	1.913176	-2.728704
H	6.796065	0.821075	-3.369298
C	10.665376	0.684910	-2.752576
H	10.533527	-1.371913	-3.472496
C	9.944702	1.867438	-2.505986
H	8.008022	2.850317	-2.576904
H	11.749249	0.658670	-2.602073
H	10.470665	2.766526	-2.167959
C	-0.970752	-0.470300	0.203883
N	-1.636834	-0.281923	-0.755227
C	-2.437197	-0.054232	-1.851857
C	-3.836185	-0.264430	-1.754100
C	-1.855318	0.414241	-3.057806
C	-4.642432	-0.020236	-2.860735
H	-4.266847	-0.625197	-0.814912
C	-2.671974	0.687074	-4.148349
H	-0.775217	0.579916	-3.112765
C	-4.072584	0.452866	-4.077006
H	-5.723857	-0.171972	-2.797299
H	-2.249163	1.091131	-5.073125
N	-4.884564	0.984919	-5.096886
N	-5.843287	0.454017	-5.713032
C	-6.089809	-0.946534	-5.741897
C	-5.102547	-1.954795	-5.612984
C	-7.426634	-1.293466	-6.052422
C	-5.472481	-3.295773	-5.758173
H	-4.054193	-1.686849	-5.453417
C	-7.792257	-2.639974	-6.152003
H	-8.151869	-0.487173	-6.200677
C	-6.815162	-3.641722	-6.009233
H	-4.710435	-4.079658	-5.692663
H	-8.830170	-2.909901	-6.371140
H	-7.092591	-4.694923	-6.123607

[{Pd_2(tpbz)}(**iso-Ph**)$_4$]$^{4+}$
G = -6362.176982 Ha

Pd	-0.438338	0.064797	4.545620
Pd	0.198883	-0.031535	-4.471023
P	-0.850439	-1.515337	2.870559
P	-0.741080	1.644209	2.846813
C	-0.557529	-0.659614	1.243088
C	-0.461344	0.753317	1.239542
C	-0.378304	-1.378714	0.046614
H	-0.445427	-2.472059	0.055644
C	0.252236	-2.975673	2.881786
C	1.569412	-2.835884	3.375583
H	1.906592	-1.874340	3.781115
C	2.441299	-3.935025	3.364195
H	3.458016	-3.826429	3.755928
C	2.003450	-5.176001	2.868018
H	2.679645	-6.037360	2.878156
C	0.694462	-5.317928	2.375727
H	0.347952	-6.288505	2.005381
C	-0.184800	-4.222577	2.377782
H	-1.211794	-4.347564	2.018325
C	-2.580197	-2.104413	2.872098
C	-2.972937	-3.051613	3.848493
H	-2.237714	-3.474369	4.541703
C	-4.311403	-3.460982	3.923419
H	-4.610636	-4.199302	4.674257
C	-5.265341	-2.923686	3.040885

H	-6.310427	-3.243059	3.106503
C	-4.879505	-1.975574	2.079045
H	-5.623444	-1.554605	1.394847
C	-3.541729	-1.560257	1.991790
H	-3.253267	-0.814483	1.243736
C	0.403497	3.070304	2.851363
C	1.780701	2.832110	3.072180
H	2.145755	1.811988	3.243440
C	2.679037	3.907564	3.095390
H	3.743301	3.723766	3.275487
C	2.211032	5.221287	2.908319
H	2.913772	6.060358	2.940171
C	0.843222	5.460044	2.696717
H	0.477614	6.483607	2.563526
C	-0.066626	4.389508	2.668583
H	-1.133977	4.582451	2.518989
C	-2.463115	2.267435	2.812033
C	-3.193468	2.304587	4.022608
H	-2.745137	1.929014	4.948856
C	-4.498037	2.820546	4.039135
H	-5.058078	2.847851	4.979383
C	-5.083302	3.295672	2.854018
H	-6.101891	3.696740	2.869636
C	-4.363625	3.253423	1.647194
H	-4.820232	3.622083	0.722893
C	-3.058206	2.739816	1.619071
H	-2.511585	2.712516	0.670327
P	0.298567	1.575338	-2.783322
P	0.233115	-1.611751	-2.748952
C	-0.005629	0.710513	-1.163654
C	-0.099199	-0.699871	-1.159602
C	-0.177428	1.431365	0.037415
H	-0.083957	2.523529	0.038455
C	-0.963628	2.894933	-2.903616
C	-2.188495	2.609285	-3.549364
H	-2.353217	1.626280	-4.007053
C	-3.186493	3.592782	-3.624343
H	-4.129755	3.372317	-4.134847
C	-2.966474	4.863063	-3.063111
H	-3.740136	5.634466	-3.137292
C	-1.748666	5.150872	-2.422419
H	-1.571422	6.145898	-2.001006
C	-0.745095	4.172484	-2.337496
H	0.211962	4.413866	-1.862467
C	1.945326	2.359472	-2.662426
C	2.281382	3.388957	-3.574068
H	1.540345	3.763529	-4.288223
C	3.569381	3.942266	-3.557422
H	3.823514	4.743415	-4.258857
C	4.530489	3.468864	-2.646779
H	5.536427	3.900844	-2.641063
C	4.202095	2.440708	-1.747578
H	4.950818	2.070753	-1.039526
C	2.915268	1.880607	-1.752795
H	2.672123	1.076732	-1.049886
C	-1.054894	-2.902024	-2.862081
C	-2.395347	-2.574490	-2.548509
H	-2.648361	-1.581716	-2.158754
C	-3.409340	-3.523636	-2.739394
H	-4.445788	-3.268325	-2.495918

C	-3.098431	-4.796515	-3.249550
H	-3.893475	-5.533517	-3.402188
C	-1.769692	-5.121462	-3.569403
H	-1.526392	-6.111964	-3.967239
C	-0.746476	-4.178588	-3.384423
H	0.285595	-4.439731	-3.639132
C	1.867529	-2.416865	-2.580440
C	2.953783	-1.916089	-3.333729
H	2.803841	-1.073702	-4.019949
C	4.222505	-2.504036	-3.212738
H	5.058518	-2.118957	-3.805510
C	4.413704	-3.589508	-2.342391
H	5.401823	-4.053048	-2.255516
C	3.336089	-4.088688	-1.588902
H	3.484189	-4.940603	-0.917562
C	2.064784	-3.508064	-1.701569
H	1.230575	-3.920165	-1.123607
C	0.235805	-1.463804	-5.886306
N	0.253263	-2.240082	-6.776825
C	0.259716	-3.132500	-7.828876
C	1.088604	-4.279605	-7.766601
C	-0.560629	-2.877165	-8.960464
C	1.092786	-5.163349	-8.842049
H	1.721332	-4.451410	-6.890638
C	-0.549531	-3.773686	-10.021227
H	-1.194124	-1.984467	-8.981199
H	1.727080	-6.054937	-8.838169
C	0.275798	-4.931454	-9.977824
H	-1.170354	-3.614563	-10.907332
N	0.376208	-5.891304	-11.004397
N	-0.459570	-5.688936	-11.965683
C	-0.376492	-6.591483	-13.030341
C	0.561389	-7.658075	-13.123904
C	0.542230	-8.485995	-14.243633
H	1.279260	-7.804713	-12.312444
C	-1.330994	-7.215661	-15.187393
C	-0.400855	-8.268367	-15.274786
H	1.258768	-9.309411	-14.329111
H	-2.056927	-7.055353	-15.990425
H	-0.406002	-8.926759	-16.149992
C	0.109858	1.357676	-5.921164
N	0.100298	2.118780	-6.826001
C	0.098595	2.992747	-7.891923
C	-0.481446	4.277138	-7.740676
C	0.676797	2.586548	-9.125068
C	-0.480393	5.146121	-8.827544
H	-0.930324	4.565163	-6.785286
C	0.674976	3.469019	-10.197240
H	1.120255	1.589598	-9.214111
H	-0.926660	6.142552	-8.755256
C	0.100126	4.763871	-10.064288
H	1.114287	3.194896	-11.160452
N	0.025326	5.721316	-11.093282
N	0.695368	5.386788	-12.144032
C	0.606353	6.275189	-13.219115
C	-0.184337	7.459085	-13.235515
C	-0.191168	8.257861	-14.376184
H	-0.770584	7.716664	-12.349336
C	1.365856	6.726908	-15.493559
C	0.581385	7.895689	-15.504360

H	-0.795231	9.170607	-14.403192
H	1.960415	6.455764	-16.371329
H	0.568242	8.532879	-16.395060
C	-0.127609	1.476572	5.937764
N	0.111866	2.262523	6.788609
C	0.388638	3.169830	7.787057
C	0.266677	4.557311	7.522571
C	0.784722	2.696989	9.067744
C	0.537468	5.461441	8.544630
H	-0.039420	4.898732	6.529301
C	1.056396	3.613360	10.074656
H	0.874071	1.620370	9.244368
H	0.446557	6.539985	8.384290
C	0.939956	5.010160	9.828213
H	1.366003	3.288492	11.071853
N	1.165137	6.017751	10.784136
N	1.672180	5.568606	11.882627
C	1.856808	6.519776	12.889408
C	1.490800	7.892348	12.791990
C	1.722431	8.737471	13.874184
H	1.034548	8.254261	11.866698
C	2.681713	6.879953	15.157429
C	2.316764	8.235749	15.055674
H	1.445037	9.794874	13.813791
H	3.141180	6.501843	16.075794
H	2.494435	8.910888	15.899547
C	-0.253600	-1.349691	5.957446
N	-0.181665	-2.130168	6.842691
C	-0.113093	-3.033338	7.882234
C	0.435371	-4.319957	7.653773
C	-0.594908	-2.655375	9.164675
C	0.500770	-5.219024	8.714111
H	0.807372	-4.586607	6.659956
C	-0.528336	-3.567737	10.209443
H	-1.018863	-1.656821	9.312268
H	0.925162	-6.218910	8.582219
C	0.017720	-4.864891	10.000026
H	-0.893964	-3.316428	11.208986
N	0.149056	-5.854708	10.992852
N	-0.419883	-5.530393	12.104897
C	-0.280632	-6.457887	13.141003
C	0.453748	-7.674458	13.053809
C	0.518914	-8.511863	14.164564
H	0.951985	-7.925961	12.113700
C	-0.867943	-6.955149	15.456635
C	-0.139667	-8.156246	15.364765
H	1.080799	-9.450082	14.112086
H	-1.374397	-6.689307	16.389545
H	-0.081594	-8.823936	16.230976
C	-1.320346	-6.377620	-14.069488
H	-2.026829	-5.548133	-13.964735
C	1.379507	5.916732	-14.355472
H	1.975925	5.000144	-14.307919
C	2.452792	6.022073	14.078930
H	2.721640	4.961793	14.118914
C	-0.939194	-6.106247	14.349348
H	-1.495202	-5.163925	14.380766

[{Pd_2(tpbz)}(*cis*-**iso-Ph**)(*trans*-**iso-Ph**)$_3$]$^{4+}$

G = -6362.154287 Ha

Pd	-0.430969	0.069144	4.509340
Pd	0.289907	0.064064	-4.487985
P	-0.749605	-1.503450	2.805761
P	-0.682280	1.655865	2.810127
C	-0.412425	-0.631373	1.197247
C	-0.321743	0.780932	1.210815
C	-0.192023	-1.337829	-0.002704
H	-0.269119	-2.431408	-0.016134
C	0.393709	-2.932455	2.855925
C	1.695151	-2.747920	3.377145
H	1.993870	-1.772345	3.779692
C	2.598450	-3.821245	3.400035
H	3.601932	-3.678709	3.814637
C	2.207583	-5.081005	2.911299
H	2.908581	-5.921598	2.945142
C	0.914115	-5.267213	2.394361
H	0.605227	-6.251790	2.028078
C	0.003715	-4.198257	2.361899
H	-1.010302	-4.356762	1.979872
C	-2.460482	-2.139993	2.733953
C	-2.882181	-3.057089	3.726939
H	-2.178628	-3.421023	4.483565
C	-4.207943	-3.511340	3.737944
H	-4.529512	-4.225512	4.502774
C	-5.121208	-3.049185	2.773479
H	-6.157041	-3.403204	2.789110
C	-4.707046	-2.132048	1.793799
H	-5.418814	-1.770842	1.044397
C	-3.381253	-1.671763	1.770528
H	-3.070634	-0.952311	1.005880
C	0.440463	3.097668	2.872740
C	1.812733	2.876680	3.137610
H	2.186838	1.859924	3.309164
C	2.693997	3.964365	3.203147
H	3.754368	3.793683	3.416021
C	2.213207	5.273367	3.015356
H	2.902213	6.121886	3.079974
C	0.849936	5.494951	2.760369
H	0.474083	6.514644	2.626039
C	-0.042561	4.411932	2.688823
H	-1.106635	4.590914	2.503185
C	-2.410126	2.254563	2.707418
C	-3.186462	2.283858	3.889303
H	-2.768343	1.917092	4.833041
C	-4.497858	2.781000	3.854840
H	-5.093703	2.802072	4.772972
C	-5.044145	3.245198	2.647011
H	-6.068286	3.631409	2.622717
C	-4.278406	3.210740	1.468731
H	-4.704636	3.570672	0.526682
C	-2.965724	2.715863	1.491498
H	-2.383172	2.693390	0.564331
P	0.556738	1.644549	-2.781458
P	0.508461	-1.519220	-2.782396
C	0.224744	0.768575	-1.172959
C	0.136367	-0.643709	-1.185917
C	0.005315	1.474719	0.027687
H	0.086721	2.567819	0.042725

C	-0.624569	3.042878	-2.847405
C	-1.895380	2.833757	-3.430593
H	-2.144830	1.859859	-3.869425
C	-2.830626	3.879190	-3.466047
H	-3.810399	3.717066	-3.927496
C	-2.502100	5.136026	-2.927309
H	-3.227130	5.955552	-2.970465
C	-1.239005	5.347332	-2.348173
H	-0.977911	6.330542	-1.943035
C	-0.297391	4.306417	-2.303517
H	0.694147	4.486659	-1.874693
C	2.247314	2.331009	-2.682515
C	2.635021	3.324892	-3.613046
H	1.912958	3.726438	-4.332153
C	3.950729	3.808385	-3.608655
H	4.245572	4.582303	-4.324599
C	4.887498	3.300401	-2.691204
H	5.915059	3.678079	-2.694716
C	4.507243	2.307226	-1.773796
H	5.237362	1.909862	-1.061245
C	3.192179	1.817041	-1.766673
H	2.908946	1.037202	-1.052061
C	-0.612879	-2.962303	-2.850006
C	-1.972758	-2.750309	-3.177250
H	-2.336091	-1.740540	-3.403674
C	-2.853925	-3.838894	-3.233917
H	-3.905060	-3.675179	-3.493256
C	-2.384437	-5.139998	-2.976313
H	-3.072888	-5.989601	-3.033018
C	-1.032390	-5.353192	-2.660602
H	-0.664646	-6.367408	-2.472846
C	-0.140936	-4.268932	-2.595862
H	0.914867	-4.441145	-2.362235
C	2.234277	-2.123259	-2.657324
C	2.993894	-2.237414	-3.845256
H	2.564583	-1.931371	-4.805322
C	4.302382	-2.740343	-3.795845
H	4.884888	-2.826809	-4.718704
C	4.863978	-3.125385	-2.567195
H	5.886492	-3.514951	-2.531253
C	4.115357	-3.006568	-1.383748
H	4.553037	-3.304858	-0.425530
C	2.804921	-2.505840	-1.421420
H	2.236211	-2.417430	-0.489746
C	0.266473	1.490954	-5.903013
N	0.242205	2.267859	-6.793538
C	0.235573	3.176038	-7.832779
C	-0.518224	4.369128	-7.710685
C	0.988358	2.897342	-9.004892
C	-0.516332	5.274566	-8.768271
H	-1.094846	4.560712	-6.800767
C	0.983207	3.814770	-10.047886
H	1.568940	1.971287	-9.069229
H	-1.090161	6.204845	-8.716910
C	0.229748	5.017043	-9.946389
H	1.553837	3.638752	-10.964035
N	0.152165	6.011340	-10.942633
N	0.860238	5.733553	-11.984797
C	0.819767	6.687312	-13.006357
C	0.066084	7.894197	-12.965899
C	0.111330	8.762684	-14.053717
H	-0.534820	8.113573	-12.079294
C	1.646718	7.255669	-15.230655
C	0.899120	8.447366	-15.185170
H	-0.464177	9.693956	-14.036351
H	2.253605	7.020650	-16.110427
H	0.926533	9.138752	-16.034134
C	-0.190788	1.473915	5.922342
N	0.002092	2.256363	6.787927
C	0.223675	3.159644	7.803725
C	0.099974	4.547527	7.542661
C	0.565290	2.682082	9.098245
C	0.314187	5.447273	8.581925
H	-0.163173	4.892668	6.538432
C	0.780652	3.594155	10.122498
H	0.657498	1.605222	9.271871
H	0.220427	6.525986	8.424368
C	0.660680	4.991322	9.880062
H	1.047174	3.265653	11.130878
N	0.833043	5.995570	10.850864
N	1.275702	5.540676	11.974652
C	1.412206	6.489268	12.991567
C	1.067228	7.865757	12.874952
C	1.245782	8.707830	13.969532
H	0.668305	8.232962	11.925575
C	2.109976	6.839472	15.303440
C	1.766133	8.199186	15.182509
H	0.983671	9.768200	13.894528
H	2.512089	6.455932	16.246142
H	1.902494	8.871944	16.035919
C	-0.279397	-1.354184	5.917407
N	-0.213524	-2.137051	6.800868
C	-0.151851	-3.044459	7.837324
C	0.445590	-4.311103	7.620887
C	-0.689276	-2.690623	9.104420
C	0.504283	-5.214238	8.678192
H	0.859313	-4.559401	6.638900
C	-0.628100	-3.606809	10.146195
H	-1.150647	-1.707417	9.242609
H	0.964806	-6.199205	8.555558
C	-0.032202	-4.883756	9.949071
H	-1.035210	-3.373793	11.134056
N	0.096467	-5.876715	10.939550
N	-0.500933	-5.567881	12.041112
C	-0.373396	-6.502302	13.072571
C	0.372909	-7.711901	12.990061
C	0.423711	-8.557742	14.095176
H	0.891682	-7.951387	12.057964
C	-1.001054	-7.023266	15.372377
C	-0.260919	-8.217423	15.285165
H	0.994611	-9.490699	14.046168
H	-1.527665	-6.769198	16.297381
H	-0.213786	-8.891530	16.147063
C	1.608339	6.376190	-14.145630
H	2.175657	5.440084	-14.142838
C	1.933823	5.984658	14.212653
H	2.188504	4.921508	14.266765
C	-1.058158	-6.166074	14.270654
H	-1.622698	-5.228730	14.298771
C	0.035519	-1.335197	-5.900288

N	-0.009887	-2.126769	-6.781872
C	-0.056247	-3.049903	-7.794753
C	-0.073141	-4.437423	-7.481950
C	-0.054379	-2.615407	-9.148253
C	-0.054171	-5.369780	-8.507671
H	-0.083107	-4.757325	-6.435654
C	-0.060755	-3.552318	-10.172367
H	-0.052628	-1.544891	-9.375251
H	-0.023476	-6.440856	-8.286671
C	-0.059998	-4.949461	-9.871438
H	-0.042869	-3.226816	-11.216456
N	0.249587	-5.891924	-10.847247
N	-0.214509	-6.132867	-11.984644
C	-1.506198	-5.757920	-12.423239
C	-2.597703	-5.419497	-11.582066
C	-1.679106	-5.840998	-13.827803
C	-3.835423	-5.131011	-12.161283
H	-2.485794	-5.429477	-10.493927
C	-2.914729	-5.516288	-14.395989
H	-0.826343	-6.153120	-14.438574
C	-3.992738	-5.165049	-13.563470
H	-4.693800	-4.894900	-11.523802
H	-3.047973	-5.561939	-15.481172
H	-4.970363	-4.942556	-14.003781

[{Pd_2(tpbz)}(*cis*-**iso-Ph**)$_2$(*trans*-**iso-Ph**)$_2$]$^{4+}$
cis groups in gem arrangement
G = -6362.128621 Ha

Pd	-0.396582	0.043111	4.528194
Pd	0.481444	0.030276	-4.451974
P	-0.621699	-1.554917	2.833790
P	-0.635258	1.607022	2.806372
C	-0.277141	-0.688539	1.224553
C	-0.218860	0.725144	1.224668
C	-0.032198	-1.400153	0.032220
H	-0.096796	-2.494299	0.026326
C	0.563222	-2.945859	2.925965
C	1.842928	-2.717082	3.481725
H	2.097951	-1.731282	3.889304
C	2.781587	-3.758454	3.529017
H	3.769282	-3.581066	3.967185
C	2.447701	-5.030308	3.030284
H	3.177179	-5.845369	3.080369
C	1.175261	-5.261119	2.480393
H	0.910880	-6.255814	2.106620
C	0.229959	-4.224276	2.422954
H	-0.767555	-4.417107	2.014121
C	-2.311601	-2.242044	2.735686
C	-2.716372	-3.188223	3.708149
H	-2.008182	-3.550060	4.461384
C	-4.030840	-3.674461	3.702535
H	-4.339091	-4.410955	4.451532
C	-4.949817	-3.215822	2.741889
H	-5.976991	-3.594590	2.744976
C	-4.552512	-2.270502	1.782156
H	-5.268547	-1.912182	1.035454
C	-3.238213	-1.778051	1.775724
H	-2.940738	-1.037041	1.026559
C	0.463793	3.067428	2.886012
C	1.821574	2.875275	3.235167
H	2.196943	1.870466	3.464632
C	2.683555	3.977854	3.315731
H	3.730885	3.830064	3.599228
C	2.197566	5.273004	3.057505
H	2.869917	6.133670	3.136077
C	0.848849	5.465691	2.716521
H	0.467833	6.474660	2.527359
C	-0.023998	4.367794	2.630636
H	-1.078068	4.525506	2.379936
C	-2.367868	2.180536	2.651052
C	-3.169876	2.226615	3.815191
H	-2.768058	1.886114	4.775643
C	-4.486022	2.706513	3.741863
H	-5.101668	2.740246	4.646459
C	-5.011804	3.137171	2.512705
H	-6.039605	3.510298	2.457984
C	-4.220605	3.085802	1.352086
H	-4.631370	3.420039	0.393796
C	-2.902873	2.607579	1.413450
H	-2.300793	2.571356	0.499164
P	0.921008	1.539349	-2.716383
P	0.547469	-1.598275	-2.769891
C	0.401633	0.700171	-1.141431
C	0.296272	-0.711086	-1.152119
C	0.131539	1.414131	0.045272
H	0.194798	2.508829	0.050772
C	0.011506	3.129018	-2.763941
C	-1.371943	3.103526	-3.061428
H	-1.875296	2.150308	-3.265058
C	-2.095443	4.302677	-3.124134
H	-3.163183	4.282085	-3.366455
C	-1.445735	5.530718	-2.900411
H	-2.010849	6.466577	-2.962546
C	-0.071134	5.558770	-2.613442
H	0.437210	6.514636	-2.448844
C	0.663595	4.362374	-2.545975
H	1.737360	4.391912	-2.334267
C	2.710747	1.897076	-2.544683
C	3.538484	1.773322	-3.684116
H	3.116277	1.432971	-4.635925
C	4.904367	2.082587	-3.596037
H	5.540146	1.985173	-4.481955
C	5.453575	2.510148	-2.376381
H	6.519771	2.749972	-2.310152
C	4.635874	2.626273	-1.238770
H	5.064407	2.957462	-0.287208
C	3.269046	2.319616	-1.315583
H	2.646917	2.411795	-0.418736
C	-0.768777	-2.869164	-2.839284
C	-2.036680	-2.510393	-3.351895
H	-2.205354	-1.500648	-3.744917
C	-3.074290	-3.454180	-3.376588
H	-4.052991	-3.175780	-3.781186
C	-2.852196	-4.758234	-2.899180
H	-3.659949	-5.496876	-2.931027
C	-1.591663	-5.118884	-2.393177
H	-1.414789	-6.138511	-2.035055
C	-0.547828	-4.179812	-2.357820
H	0.437770	-4.472884	-1.980965
C	2.159528	-2.454872	-2.670604

C	2.476036	-3.414757	-3.662508
H	1.743532	-3.682010	-4.432003
C	3.732169	-4.036253	-3.656651
H	3.970557	-4.783287	-4.420609
C	4.682976	-3.698986	-2.676868
H	5.664809	-4.183272	-2.679437
C	4.375115	-2.739333	-1.698753
H	5.115781	-2.475020	-0.936982
C	3.119127	-2.112824	-1.691999
H	2.892647	-1.362325	-0.927661
C	-0.239647	1.462887	5.938814
N	-0.101264	2.252894	6.807994
C	0.057975	3.163598	7.828966
C	-0.125004	4.544768	7.567292
C	0.395578	2.699636	9.129448
C	0.026214	5.451247	8.611813
H	-0.384802	4.879666	6.558717
C	0.547921	3.618549	10.158935
H	0.534330	1.627836	9.303536
H	-0.114978	6.524717	8.453799
C	0.367830	5.009126	9.915986
H	0.809342	3.300579	11.172014
N	0.473665	6.018225	10.891422
N	0.915514	5.581679	12.022861
C	0.987036	6.533396	13.043667
C	0.579511	7.892221	12.922421
C	0.696479	8.739163	14.021592
H	0.182172	8.242414	11.965978
C	1.622097	6.910683	15.369332
C	1.216561	8.252836	15.243770
H	0.385797	9.786077	13.943052
H	2.023444	6.544293	16.319162
H	1.304126	8.929208	16.100720
C	-0.248895	-1.366540	5.949613
N	-0.184864	-2.143465	6.838294
C	-0.124587	-3.046135	7.879000
C	0.525622	-4.290354	7.685896
C	-0.715960	-2.709927	9.126631
C	0.582497	-5.188835	8.747303
H	0.980425	-4.524845	6.718782
C	-0.655535	-3.621429	10.172614
H	-1.217267	-1.744037	9.246675
H	1.082721	-6.156381	8.642815
C	-0.007170	-4.875970	9.998901
H	-1.103062	-3.401701	11.145922
N	0.125547	-5.861726	10.996189
N	-0.519089	-5.573242	12.076427
C	-0.390332	-6.500325	13.114547
C	0.403567	-7.680801	13.060510
C	0.449952	-8.521989	14.169409
H	0.961509	-7.902202	12.146744
C	-1.073169	-7.040301	15.394156
C	-0.285870	-8.205639	15.335170
H	1.057123	-9.432676	14.142116
H	-1.639214	-6.804554	16.300540
H	-0.241626	-8.875774	16.200315
C	1.508028	6.051153	14.273965
H	1.811722	5.001125	14.331435
C	-1.126389	-6.188048	14.288371
H	-1.726628	-5.272739	14.294941

C	0.013246	-1.323756	-5.853963
N	-0.062048	-2.168346	-6.682949
C	-0.165077	-3.166665	-7.619798
C	-0.678150	-4.436201	-7.236301
C	0.269163	-2.937462	-8.952557
C	-0.722680	-5.465280	-8.165187
H	-1.024108	-4.593480	-6.210076
C	0.200525	-3.964086	-9.885719
H	0.659630	-1.955112	-9.235550
H	-1.077798	-6.459773	-7.878762
C	-0.297008	-5.248745	-9.509150
H	0.552272	-3.802053	-10.908700
N	-0.084876	-6.365664	-10.316178
N	-0.349916	-6.611508	-11.515122
C	-1.336251	-5.941069	-12.276876
C	-2.428629	-5.206139	-11.747788
C	-1.218686	-6.148244	-13.674233
C	-3.365932	-4.657753	-12.626377
H	-2.563690	-5.110774	-10.666474
C	-2.144449	-5.563446	-14.543742
H	-0.390517	-6.762783	-14.040524
C	-3.219374	-4.822350	-14.020283
H	-4.230014	-4.113556	-12.231138
H	-2.047632	-5.706625	-15.624314
H	-3.965331	-4.393023	-14.697320
C	0.613684	1.469762	-5.849164
N	0.458817	2.322576	-6.659000
C	0.307894	3.325211	-7.582760
C	0.459775	4.679930	-7.178181
C	-0.018424	3.007408	-8.928575
C	0.258456	5.696098	-8.099983
H	0.714685	4.909343	-6.139195
C	-0.194122	4.027105	-9.854644
H	-0.127127	1.960575	-9.228125
H	0.331849	6.745155	-7.798157
C	-0.056672	5.392332	-9.457519
H	-0.461616	3.792032	-10.888863
N	-0.522687	6.424799	-10.268459
N	-0.293654	6.747367	-11.456043
C	0.857354	6.370357	-12.187332
C	2.084034	5.930512	-11.625637
C	0.736491	6.567749	-13.585926
C	3.159063	5.658042	-12.474729
H	2.203355	5.851417	-10.541185
C	1.809876	6.257828	-14.426592
H	-0.209535	6.955696	-13.976067
C	3.021205	5.807042	-13.871388
H	4.120281	5.345396	-12.053747
H	1.715465	6.392553	-15.508453
H	3.874345	5.596386	-14.524769

[{Pd_2(tpbz)}(*cis*-**iso-Ph**)$_2$(*trans*-**iso-Ph**)$_2$]$^{4+}$
cis groups in *cis* arrangement
G = -6362.128630 Ha

Pd	-0.396681	0.043049	4.528291
Pd	0.481138	0.030289	-4.451832
P	-0.622121	-1.554917	2.833881
P	-0.635565	1.606988	2.806513
C	-0.277451	-0.688548	1.224669
C	-0.219146	0.725132	1.224794

C	-0.032504	-1.400172	0.032343
H	-0.097124	-2.494318	0.026461
C	0.562468	-2.946146	2.925947
C	1.842367	-2.717613	3.481367
H	2.097748	-1.731817	3.888728
C	2.780757	-3.759227	3.528611
H	3.768604	-3.582033	3.966514
C	2.446408	-5.031079	3.030175
H	3.175679	-5.846328	3.080231
C	1.173777	-5.261644	2.480629
H	0.909036	-6.256331	2.107090
C	0.228737	-4.224555	2.423239
H	-0.768923	-4.417182	2.014668
C	-2.312191	-2.241651	2.735830
C	-2.717298	-3.187288	3.708683
H	-2.009310	-3.548864	4.462233
C	-4.031840	-3.673322	3.703062
H	-4.340350	-4.409395	4.452366
C	-4.950562	-3.215010	2.742013
H	-5.977796	-3.593615	2.745089
C	-4.552922	-2.270230	1.781889
H	-5.268757	-1.912167	1.034872
C	-3.238543	-1.777990	1.775461
H	-2.940804	-1.037406	1.025982
C	0.463482	3.067405	2.886133
C	1.821387	2.875154	3.234758
H	2.196849	1.870276	3.463769
C	2.683385	3.977715	3.315382
H	3.730814	3.829843	3.598473
C	2.197296	5.272945	3.057745
H	2.869668	6.133590	3.136359
C	0.848460	5.465728	2.717294
H	0.467367	6.474752	2.528583
C	-0.024408	4.367848	2.631357
H	-1.078563	4.525638	2.381070
C	-2.368197	2.180417	2.651228
C	-3.170589	2.225243	3.815146
H	-2.769027	1.883848	4.775388
C	-4.486780	2.705034	3.741857
H	-5.102727	2.737803	4.646284
C	-5.012209	3.136834	2.512953
H	-6.040036	3.509895	2.458260
C	-4.220628	3.086697	1.352535
H	-4.631127	3.421816	0.394440
C	-2.902858	2.608587	1.413861
H	-2.300488	2.573306	0.499728
P	0.920783	1.539323	-2.716233
P	0.547172	-1.598272	-2.769780
C	0.401360	0.700143	-1.141296
C	0.295979	-0.711111	-1.151998
C	0.131273	1.414111	0.045401
H	0.194570	2.508806	0.050899
C	0.011376	3.129042	-2.763744
C	-1.372086	3.103617	-3.061183
H	-1.875488	2.150429	-3.264830
C	-2.095537	4.302801	-3.123810
H	-3.163286	4.282264	-3.366097
C	-1.445768	5.530806	-2.900059
H	-2.010844	6.466692	-2.962142
C	-0.071158	5.558789	-2.613137

H	0.437235	6.514625	-2.448521
C	0.663524	4.362358	-2.545746
H	1.737300	4.391845	-2.334083
C	2.710547	1.896911	-2.544553
C	3.538282	1.772970	-3.683974
H	3.116045	1.432558	-4.635748
C	4.904186	2.082129	-3.595921
H	5.539969	1.984569	-4.481820
C	5.453424	2.509772	-2.376304
H	6.519641	2.749512	-2.310098
C	4.635731	2.626083	-1.238710
H	5.064285	2.957344	-0.287182
C	3.268876	2.319528	-1.315496
H	2.646754	2.411849	-0.418659
C	-0.769158	-2.869083	-2.839256
C	-2.036997	-2.510293	-3.351995
H	-2.205599	-1.500560	-3.745080
C	-3.074643	-3.454043	-3.376734
H	-4.053293	-3.175620	-3.781440
C	-2.852647	-4.758077	-2.899236
H	-3.660428	-5.496687	-2.931118
C	-1.592173	-5.118747	-2.393095
H	-1.415375	-6.138361	-2.034897
C	-0.548305	-4.179717	-2.357694
H	0.437244	-4.472817	-1.980738
C	2.159172	-2.454984	-2.670429
C	2.475642	-3.414823	-3.662392
H	1.743144	-3.681981	-4.431927
C	3.731723	-4.036417	-3.656533
H	3.970086	-4.783418	-4.420531
C	4.682523	-3.699286	-2.676692
H	5.664318	-4.183649	-2.679259
C	4.374702	-2.739676	-1.698526
H	5.115362	-2.475463	-0.936715
C	3.118761	-2.113068	-1.691777
H	2.892307	-1.362596	-0.927404
C	-0.239826	1.462857	5.938885
N	-0.101404	2.252931	6.807997
C	0.057800	3.163735	7.828885
C	-0.125356	4.544865	7.567115
C	0.395528	2.699913	9.129383
C	0.025808	5.451444	8.611555
H	-0.385256	4.879651	6.558529
C	0.547829	3.618925	10.158788
H	0.534404	1.628142	9.303548
H	-0.115549	6.524881	8.453467
C	0.367588	5.009463	9.915733
H	0.809344	3.301062	11.171878
N	0.473245	6.018593	10.891138
N	0.916060	5.582371	12.022315
C	0.987240	6.534065	13.043169
C	0.578214	7.892472	12.922312
C	0.694961	8.739422	14.021501
H	0.179931	8.242350	11.966146
C	1.623331	6.911777	15.368482
C	1.216305	8.253515	15.243308
H	0.383128	9.786016	13.943262
H	2.025649	6.545712	16.318026
H	1.303687	8.929890	16.100275
C	-0.248492	-1.366600	5.949632

N	-0.184363	-2.143625	6.838221
C	-0.123970	-3.046369	7.878850
C	0.525589	-4.290875	7.685365
C	-0.714542	-2.709944	9.126800
C	0.582630	-5.189417	8.746707
H	0.979798	-4.525521	6.718010
C	-0.654003	-3.621526	10.172708
H	-1.215321	-1.743820	9.247155
H	1.082424	-6.157155	8.641934
C	-0.006336	-4.876372	9.998591
H	-1.100943	-3.401639	11.146252
N	0.126624	-5.862052	10.995887
N	-0.519006	-5.574290	12.075714
C	-0.389440	-6.500952	13.114111
C	0.406514	-7.680079	13.060896
C	0.453554	-8.520939	14.170016
H	0.965464	-7.900740	12.147566
C	-1.072982	-7.041597	15.393347
C	-0.283643	-8.205594	15.335180
H	1.062301	-9.430592	14.143357
H	-1.640082	-6.806623	16.299272
H	-0.238876	-8.875467	16.200502
C	1.509492	6.052239	14.273095
H	1.814324	5.002525	14.330270
C	-1.126870	-6.189676	14.287337
H	-1.728682	-5.275396	14.293275
C	0.013013	-1.323714	-5.853878
N	-0.062137	-2.168252	-6.682933
C	-0.164945	-3.166510	-7.619862
C	-0.677761	-4.436190	-7.236481
C	0.269267	-2.937112	-8.952600
C	-0.722064	-5.465200	-8.165444
H	-1.023700	-4.593624	-6.210272
C	0.200858	-3.963670	-9.885846
H	0.659531	-1.954658	-9.235511
H	-1.076987	-6.459789	-7.879110
C	-0.296403	-5.248480	-9.509392
H	0.552592	-3.801478	-10.908806
N	-0.084050	-6.365259	-10.316484
N	-0.348839	-6.611078	-11.515493
C	-1.335235	-5.940834	-12.277323
C	-2.427772	-5.206061	-11.748339
C	-1.217529	-6.148027	-13.674669
C	-3.365091	-4.657850	-12.627017
H	-2.562927	-5.110677	-10.667038
C	-2.143321	-5.563410	-14.544266
H	-0.389228	-6.762439	-14.040874
C	-3.218402	-4.822470	-14.020909
H	-4.229287	-4.113772	-12.231863
H	-2.046401	-5.706608	-15.624826
H	-3.964377	-4.393279	-14.698012
C	0.613304	1.469805	-5.848975
N	0.458669	2.322732	-6.658727
C	0.307986	3.325515	-7.582380
C	0.460805	4.680115	-7.177768
C	-0.019023	3.007980	-8.928085
C	0.259727	5.696458	-8.099435
H	0.716240	4.909313	-6.138864
C	-0.194483	4.027845	-9.854022
H	-0.128461	1.961230	-9.227658
H	0.333842	6.745453	-7.797571
C	-0.056088	5.392957	-9.456862
H	-0.462510	3.792993	-10.888153
N	-0.521855	6.425769	-10.267564
N	-0.292972	6.748244	-11.455208
C	0.857565	6.370483	-12.186865
C	2.084187	5.930016	-11.625539
C	0.736348	6.567805	-13.585436
C	3.158786	5.656862	-12.474961
H	2.203830	5.850981	-10.541118
C	1.809275	6.257185	-14.426429
H	-0.209584	6.956254	-13.975306
C	3.020542	5.805784	-13.871586
H	4.119974	5.343743	-12.054261
H	1.714575	6.391849	-15.508272
H	3.873346	5.594590	-14.525233

[{Pd$_2$(tpbz)}(*cis*-**iso-Ph**)$_2$(*trans*-**iso-Ph**)$_2$]$^{4+}$
cis groups in *trans* arrangement
G = -6362.130286 Ha

Pd	0.658999	-0.018142	4.379725
Pd	-0.287342	-0.183868	-4.599065
P	0.897772	-1.627393	2.700013
P	0.888860	1.534064	2.641620
C	0.478720	-0.787990	1.095504
C	0.539335	0.625165	1.055309
C	0.138567	-1.508702	-0.068297
H	0.080101	-2.602738	-0.033267
C	2.634320	-2.194937	2.556711
C	3.457845	-2.150606	3.705350
H	3.067350	-1.749811	4.647161
C	4.779226	-2.617778	3.639266
H	5.412044	-2.581294	4.531854
C	5.288449	-3.124924	2.432699
H	6.320404	-3.487312	2.383620
C	4.475256	-3.163887	1.286692
H	4.872209	-3.557299	0.345241
C	3.152490	-2.699654	1.341351
H	2.533115	-2.734811	0.438756
C	-0.192356	-3.093229	2.807307
C	0.317856	-4.402106	2.663397
H	1.384744	-4.564056	2.478588
C	-0.551260	-5.501476	2.770014
H	-0.154120	-6.516447	2.663553
C	-1.918723	-5.301473	3.020894
H	-2.589871	-6.161969	3.110789
C	-2.426558	-3.997716	3.170434
H	-3.490062	-3.842958	3.379909
C	-1.568514	-2.894223	3.069741
H	-1.963830	-1.880820	3.210899
C	2.574166	2.227349	2.510860
C	3.495454	1.746338	1.553957
H	3.196516	0.984837	0.826176
C	4.806203	2.247734	1.536133
H	5.517717	1.876680	0.791281
C	5.205835	3.218832	2.468871
H	6.230041	3.605169	2.452437
C	4.292863	3.693389	3.427483
H	4.602942	4.449552	4.155858
C	2.982007	3.198366	3.457410

H	2.278745	3.572664	4.209286
C	-0.300882	2.925950	2.686704
C	-1.583009	2.709687	3.242266
H	-1.837708	1.734516	3.674740
C	-2.523349	3.751036	3.260211
H	-3.511756	3.583545	3.700954
C	-2.188733	5.011175	2.732347
H	-2.918145	5.827413	2.762169
C	-0.914711	5.229073	2.180607
H	-0.649242	6.214253	1.783190
C	0.031745	4.191789	2.152337
H	1.030179	4.375904	1.741817
P	-0.487964	1.425229	-2.916487
P	-0.475501	-1.738641	-2.860035
C	-0.062460	0.584703	-1.314859
C	-0.122722	-0.828238	-1.275037
C	0.277839	1.305721	-0.151039
H	0.331820	2.400231	-0.184505
C	-2.219515	2.005571	-2.758524
C	-3.009160	2.092077	-3.929092
H	-2.598369	1.780020	-4.895251
C	-4.323905	2.575195	-3.854643
H	-4.929581	2.640175	-4.764256
C	-4.862071	2.967784	-2.617932
H	-5.889550	3.341679	-2.562397
C	-4.083450	2.876537	-1.451570
H	-4.502528	3.180731	-0.486928
C	-2.766483	2.395812	-1.514349
H	-2.174552	2.328901	-0.595478
C	0.608515	2.883720	-3.039010
C	0.122818	4.187337	-2.795582
H	-0.929249	4.347651	-2.537892
C	0.995293	5.283666	-2.902082
H	0.616707	6.295365	-2.722457
C	2.341976	5.085463	-3.248940
H	3.015386	5.944282	-3.338087
C	2.824984	3.787270	-3.495970
H	3.871589	3.634881	-3.779391
C	1.962692	2.686631	-3.397531
H	2.335933	1.678571	-3.615255
C	-2.147480	-2.459630	-2.706414
C	-3.083665	-1.940204	-1.784705
H	-2.801780	-1.135612	-1.097502
C	-4.388198	-2.456590	-1.751045
H	-5.111574	-2.054791	-1.034113
C	-4.766775	-3.481437	-2.633708
H	-5.786240	-3.879485	-2.605600
C	-3.838939	-3.994857	-3.557167
H	-4.132579	-4.793241	-4.246237
C	-2.533805	-3.485391	-3.602212
H	-1.818786	-3.891132	-4.325971
C	0.734820	-3.111494	-2.933154
C	1.983118	-2.886876	-3.557577
H	2.197708	-1.917210	-4.023322
C	2.940353	-3.911997	-3.599150
H	3.902422	-3.738049	-4.092429
C	2.656127	-5.164080	-3.025387
H	3.397867	-5.968248	-3.073318
C	1.415468	-5.390825	-2.404993
H	1.188411	-6.370808	-1.972447

C	0.452317	-4.370114	-2.353897
H	-0.522119	-4.563051	-1.892600
C	-0.269154	-1.634880	-5.988744
N	-0.254148	-2.429162	-6.863998
C	-0.260156	-3.357751	-7.884808
C	0.514861	-4.536900	-7.759049
C	-1.045753	-3.113190	-9.042905
C	0.501198	-5.462370	-8.798981
H	1.117270	-4.701821	-6.860678
C	-1.052897	-4.050931	-10.067667
H	-1.640997	-2.196750	-9.110910
H	1.092388	-6.381574	-8.745243
C	-0.280593	-5.240768	-9.961204
H	-1.649057	-3.901519	-10.972212
N	-0.205492	-6.247465	-10.944709
N	-1.003970	-6.039542	-11.936885
C	-0.949395	-6.994563	-12.956874
C	-0.079975	-8.121451	-12.973787
C	-0.122427	-8.998867	-14.054589
H	0.604842	-8.274361	-12.135370
C	-1.884251	-7.659388	-15.110607
C	-1.021727	-8.771579	-15.122182
H	0.541454	-9.869130	-14.080968
H	-2.577007	-7.492237	-15.941093
H	-1.046083	-9.469365	-15.965993
C	0.439822	1.425301	5.756120
N	0.366270	2.243306	6.606730
C	0.284317	3.194674	7.602219
C	-0.384969	4.416643	7.344577
C	0.871347	2.930130	8.868631
C	-0.464951	5.364996	8.360376
H	-0.836060	4.595905	6.364013
C	0.787920	3.890521	9.868584
H	1.387461	1.979718	9.038953
H	-0.980650	6.317530	8.205306
C	0.120492	5.123830	9.629380
H	1.231730	3.726497	10.854522
N	-0.038150	6.153359	10.577756
N	0.614329	5.937076	11.669488
C	0.455228	6.907448	12.663495
C	-0.380792	8.054589	12.557212
C	-0.455038	8.945056	13.625505
H	-0.948506	8.212464	11.636288
C	1.123370	7.578760	14.912248
C	0.294414	8.710922	14.801814
H	-1.094924	9.830894	13.557718
H	1.699755	7.406577	15.826401
H	0.228015	9.419086	15.634636
C	0.544842	-1.408233	5.823841
N	0.254226	-2.183839	6.672667
C	-0.066028	-3.092423	7.647848
C	-0.115365	-4.477689	7.328845
C	-0.369672	-2.643523	8.961943
C	-0.490291	-5.390622	8.302331
H	0.124555	-4.808593	6.313946
C	-0.721700	-3.563440	9.940061
H	-0.323699	-1.575445	9.195537
H	-0.571623	-6.455769	8.066064
C	-0.786730	-4.956710	9.629036
H	-0.974637	-3.224843	10.948911

N	-1.425257	-5.851209	10.482647
N	-1.286028	-6.126271	11.695758
C	-0.111999	-5.885175	12.446813
C	1.183730	-5.676444	11.907023
C	2.263086	-5.516540	12.778815
H	1.344767	-5.688272	10.825108
C	0.781405	-5.772724	14.710152
C	2.063470	-5.550420	14.175645
H	3.272946	-5.383237	12.377180
H	0.636113	-5.819335	15.793745
H	2.919815	-5.430283	14.847486
C	-1.849493	-6.771506	-14.032281
H	-2.504198	-5.895481	-13.986291
C	1.204607	6.677598	13.847610
H	1.837527	5.785789	13.894903
C	-0.302493	-5.969301	13.849131
H	-1.308304	-6.180404	14.225208
C	-0.108418	1.195629	-6.041777
N	-0.097114	1.974106	-6.935906
C	-0.094149	2.882617	-7.963048
C	-0.071680	4.274543	-7.671027
C	-0.147240	2.428436	-9.308984
C	-0.135159	5.191752	-8.708584
H	-0.022261	4.609544	-6.630585
C	-0.185700	3.350431	-10.345868
H	-0.153098	1.354724	-9.520279
H	-0.162081	6.265816	-8.502025
C	-0.181215	4.751747	-10.065304
H	-0.243152	3.009719	-11.383660
N	-0.534305	5.678919	-11.041481
N	-0.116250	5.902829	-12.200345
C	1.157971	5.522182	-12.683373
C	2.283715	5.203218	-11.880686
C	1.273978	5.578354	-14.095067
C	3.498136	4.906748	-12.503674
H	2.215875	5.234533	-10.789315
C	2.486691	5.245516	-14.706309
H	0.396316	5.876495	-14.676876
C	3.598629	4.913436	-13.911403
H	4.382367	4.685771	-11.896829
H	2.575843	5.270247	-15.796683
H	4.558443	4.684923	-14.386449

$[Pt(dppp)($*trans*-**iso-Ph**$)_2]^{2+}$

G = -3175.661219 Ha

Pt	-0.008261	-0.382886	1.827291
P	1.801926	-0.318000	3.368470
P	-1.614598	-0.539744	3.574229
C	1.530604	-1.298866	4.922778
H	1.535069	-2.362119	4.616946
H	2.438107	-1.159124	5.537516
C	0.264136	-0.968157	5.731799
H	0.355514	-1.466828	6.714279
H	0.204305	0.115764	5.942548
C	-1.033576	-1.469529	5.074219
H	-1.872015	-1.448996	5.793592
H	-0.932426	-2.525333	4.759448
C	2.175067	1.402304	3.867948
C	1.872001	2.461285	2.981528
H	1.375892	2.249720	2.027403
C	2.202976	3.781552	3.319972
H	1.968023	4.595393	2.626204
C	2.831050	4.058177	4.545715
H	3.086485	5.089448	4.810149
C	3.132165	3.011783	5.433275
H	3.622001	3.223775	6.389159
C	2.809566	1.687349	5.099567
H	3.063819	0.887470	5.802595
C	-2.158386	1.113764	4.137276
C	-2.079285	2.209990	3.248056
H	-1.652265	2.072165	2.248237
C	-2.543820	3.473334	3.642383
H	-2.481948	4.316383	2.946501
C	-3.083680	3.656171	4.926263
H	-3.443574	4.643351	5.233906
C	-3.162444	2.572513	5.816807
H	-3.582988	2.711473	6.818015
C	-2.704913	1.304110	5.428133
H	-2.786516	0.472313	6.135452
C	3.337480	-1.072841	2.699732
C	4.588288	-0.435751	2.840091
H	4.656771	0.558189	3.293977
C	5.754235	-1.085279	2.398957
H	6.724126	-0.590367	2.515197
C	5.677749	-2.363386	1.821611
H	6.589527	-2.868268	1.486066
C	4.430864	-2.997019	1.674832
H	4.368541	-3.992083	1.222276
C	3.261913	-2.355353	2.109065
H	2.291955	-2.853105	1.983848
C	-3.097909	-1.502624	3.075126
C	-2.911885	-2.766415	2.468376
H	-1.902314	-3.126370	2.232845
C	-4.021870	-3.566304	2.160157
H	-3.873288	-4.546560	1.695499
C	-5.320555	-3.109481	2.448949
H	-6.185529	-3.738141	2.213208
C	-5.508056	-1.849550	3.041050
H	-6.518081	-1.491502	3.266270
C	-4.401129	-1.042720	3.356762
H	-4.555600	-0.063876	3.822105
C	1.303036	-0.247930	0.330303
N	2.042265	-0.163133	-0.587196
C	2.942270	-0.072672	-1.629021
C	2.462834	0.008962	-2.957992
C	4.332703	-0.062863	-1.343612
C	3.384102	0.102680	-3.998529
H	1.386755	-0.004330	-3.153824
C	5.237188	0.033106	-2.394454
H	4.671951	-0.134345	-0.305306
H	3.055583	0.166553	-5.040217
C	4.775709	0.118314	-3.735393
H	6.316161	0.045443	-2.217558
N	5.606717	0.218090	-4.874226
N	6.859924	0.241814	-4.581442
C	7.725150	0.337498	-5.681992
C	7.310854	0.400816	-7.040059
C	9.104521	0.369276	-5.356251
C	8.273247	0.494061	-8.043967
H	6.241866	0.374664	-7.268204

C	10.061680	0.463364	-6.371178
H	9.388102	0.318910	-4.300122
C	9.646853	0.525614	-7.713812
H	7.965920	0.543148	-9.093884
H	11.127357	0.488407	-6.122818
H	10.393841	0.599133	-8.511474
C	-1.505351	-0.366851	0.507853
N	-2.362782	-0.319258	-0.303210
C	-3.391091	-0.274488	-1.222461
C	-4.682112	-0.709326	-0.838352
C	-3.133778	0.209055	-2.531619
C	-5.710505	-0.656338	-1.776815
H	-4.854548	-1.080579	0.176122
C	-4.172414	0.256882	-3.453538
H	-2.125741	0.542320	-2.798110
H	-6.723203	-0.984223	-1.523391
C	-5.475532	-0.175030	-3.087848
H	-4.015257	0.625392	-4.470920
N	-6.600151	-0.171956	-3.943902
N	-6.340171	0.299627	-5.112971
C	-7.425795	0.317138	-6.001721
C	-8.735221	-0.143559	-5.696557
C	-7.137594	0.844019	-7.285892
C	-9.728953	-0.071493	-6.671230
H	-8.934914	-0.545974	-4.699729
C	-8.142083	0.912067	-8.256217
H	-6.118535	1.189847	-7.486557
C	-9.436490	0.454834	-7.949612
H	-10.741488	-0.423636	-6.447703
H	-7.922778	1.318622	-9.248482
H	-10.225748	0.506506	-8.707286

[Pt(dppp)(*trans*-**iso-Ph**)(*cis*-**iso-Ph**)]$^{2+}$
G = -3175.638380 Ha

Pt	-0.022496	-0.346768	1.736474
P	1.726312	-0.376144	3.347668
P	-1.656531	-0.919739	3.366596
C	1.503232	-1.619200	4.710108
H	1.614093	-2.612516	4.235717
H	2.376064	-1.505387	5.378080
C	0.188871	-1.538471	5.506158
H	0.297621	-2.184007	6.397094
H	0.022667	-0.514090	5.888092
C	-1.037239	-2.033215	4.719139
H	-1.894636	-2.204735	5.394664
H	-0.829846	-3.008877	4.240549
C	1.922118	1.265286	4.132354
C	1.556218	2.424653	3.410450
H	1.115424	2.329146	2.411484
C	1.754209	3.695649	3.969521
H	1.472044	4.588640	3.402235
C	2.310065	3.821692	5.253539
H	2.461641	4.814266	5.690021
C	2.672318	2.674320	5.978547
H	3.106188	2.768978	6.979241
C	2.483467	1.399128	5.423730
H	2.784177	0.520124	6.003022
C	-2.357921	0.567561	4.168881
C	-2.343419	1.800587	3.477625
H	-1.872280	1.866889	2.490349
C	-2.927290	2.938971	4.052634
H	-2.914621	3.889368	3.509120
C	-3.523093	2.860061	5.322264
H	-3.976035	3.749939	5.771334
C	-3.538193	1.639208	6.017174
H	-4.001814	1.574169	7.006835
C	-2.961348	0.494374	5.446345
H	-2.995192	-0.448442	6.002107
C	3.348691	-0.873880	2.641820
C	4.527687	-0.159754	2.941726
H	4.485160	0.752011	3.546225
C	5.765486	-0.626429	2.466107
H	6.679244	-0.073033	2.706869
C	5.831696	-1.799855	1.696859
H	6.799419	-2.164982	1.337437
C	4.656253	-2.508653	1.390319
H	4.704960	-3.422604	0.789232
C	3.416475	-2.048035	1.857012
H	2.503014	-2.602366	1.607242
C	-3.037798	-1.905064	2.660344
C	-2.727640	-3.030980	1.862977
H	-1.685053	-3.261992	1.610313
C	-3.755658	-3.857224	1.386134
H	-3.510737	-4.731247	0.773759
C	-5.095857	-3.562942	1.695327
H	-5.896644	-4.212399	1.326702
C	-5.407004	-2.438444	2.477446
H	-6.449626	-2.206734	2.718982
C	-4.382779	-1.607091	2.962940
H	-4.633494	-0.735037	3.575448
C	-1.474817	-0.241316	0.374221
N	-2.308502	-0.133978	-0.455889
C	-3.310658	-0.027920	-1.398901
C	-3.049867	0.641608	-2.617939
C	-4.584566	-0.589472	-1.123087
C	-4.072310	0.745953	-3.558266
H	-2.061307	1.070465	-2.806203
C	-5.592313	-0.477001	-2.073923
H	-4.754865	-1.103237	-0.171686
H	-3.913350	1.258331	-4.511872
C	-5.350369	0.191566	-3.303650
H	-6.587322	-0.896088	-1.900807
N	-6.302753	0.365981	-4.333579
N	-7.440879	-0.169974	-4.061949
C	-8.421625	-0.016354	-5.054038
C	-8.235380	0.672928	-6.282835
C	-9.672492	-0.612293	-4.754688
C	-9.293214	0.755663	-7.186707
H	-7.262349	1.125314	-6.492709
C	-10.727048	-0.523616	-5.668663
H	-9.781152	-1.135168	-3.799094
C	-10.538004	0.159624	-6.883538
H	-9.161476	1.284613	-8.136483
H	-11.693991	-0.982818	-5.440319
H	-11.361879	0.231349	-7.601687
C	1.315907	0.149558	0.343895
N	2.075927	0.470676	-0.503899
C	3.003863	0.847782	-1.446496
C	2.574224	1.252139	-2.736014
C	4.381472	0.853075	-1.104484

C	3.514669	1.650347	-3.679310
H	1.508030	1.243267	-2.981945
C	5.312153	1.280146	-2.043262
H	4.694477	0.536764	-0.104901
C	4.900336	1.663748	-3.349496
H	3.194020	1.973790	-4.673748
H	6.375052	1.332069	-1.788521
N	5.831945	2.331036	-4.165078
N	6.083672	2.178530	-5.386919
C	5.711842	1.029575	-6.137176
C	5.531133	-0.272201	-5.607073
C	5.651888	1.245276	-7.535080
C	5.254088	-1.331322	-6.477160
H	5.656111	-0.456834	-4.536260
C	5.331996	0.185779	-8.390437
H	5.851118	2.252387	-7.914593
C	5.136759	-1.103435	-7.862904
H	5.143383	-2.346034	-6.080295
H	5.263739	0.355733	-9.469564
H	4.921398	-1.941051	-8.534655

[Pt(dppp)(*cis*-**iso-Ph**)$_2$]$^{2+}$
G = -3175.614390 Ha

Pt	0.107252	-0.847420	2.019218
P	1.725526	-0.596347	3.742556
P	-1.682452	-0.317558	3.492682
C	1.133110	-1.076296	5.437048
H	0.996051	-2.173847	5.413045
H	1.979348	-0.893401	6.123598
C	-0.141729	-0.377410	5.940608
H	-0.238943	-0.595721	7.020044
H	-0.046657	0.721219	5.857203
C	-1.424714	-0.869127	5.248257
H	-2.321743	-0.547887	5.807748
H	-1.461538	-1.974696	5.228571
C	2.325709	1.129766	3.850137
C	2.266689	1.955431	2.704327
H	1.824476	1.570302	1.778348
C	2.767812	3.264699	2.751350
H	2.719875	3.897225	1.858836
C	3.324668	3.763696	3.940523
H	3.711927	4.787139	3.977401
C	3.384244	2.950226	5.084287
H	3.817507	3.335852	6.012833
C	2.890059	1.637393	5.043763
H	2.956280	1.018878	5.944797
C	-2.002850	1.484298	3.534379
C	-1.655612	2.275091	2.415216
H	-1.158449	1.812929	1.554593
C	-1.942104	3.648168	2.405179
H	-1.672096	4.253157	1.533369
C	-2.569461	4.245313	3.511119
H	-2.789710	5.317774	3.503554
C	-2.914449	3.466835	4.628398
H	-3.403654	3.928751	5.492066
C	-2.636299	2.091378	4.643948
H	-2.923961	1.503253	5.521516
C	3.177954	-1.701191	3.521209
C	4.495835	-1.222440	3.674395
H	4.681541	-0.159867	3.861647
C	5.576682	-2.117651	3.592750
H	6.597869	-1.743238	3.720154
C	5.348867	-3.484149	3.360797
H	6.193217	-4.179310	3.307788
C	4.035928	-3.961940	3.199381
H	3.855707	-5.026096	3.015106
C	2.951929	-3.075388	3.276028
H	1.931167	-3.454651	3.141028
C	-3.249133	-1.172805	3.053792
C	-3.216658	-2.565680	2.812271
H	-2.261361	-3.105478	2.804925
C	-4.409396	-3.264628	2.575767
H	-4.379906	-4.343934	2.393922
C	-5.638044	-2.580328	2.573930
H	-6.568486	-3.129152	2.395139
C	-5.672017	-1.194903	2.802928
H	-6.627526	-0.659893	2.803180
C	-4.481742	-0.486608	3.043500
H	-4.517672	0.592164	3.226831
C	1.581358	-1.276109	0.748148
N	2.422590	-1.543279	-0.039401
C	3.417749	-1.882063	-0.926718
C	4.731709	-2.119202	-0.448520
C	3.111184	-2.017111	-2.305388
C	5.731026	-2.478303	-1.346570
H	4.947405	-2.017199	0.619539
C	4.108092	-2.405998	-3.191250
H	2.091647	-1.832773	-2.656672
C	5.436884	-2.621099	-2.732684
H	6.746326	-2.673793	-0.989590
H	3.883101	-2.557453	-4.251277
N	6.338307	-3.264001	-3.601640
N	7.542597	-2.999162	-3.845036
C	8.158012	-1.752825	-3.544578
C	7.489209	-0.505460	-3.476373
C	9.568463	-1.812617	-3.439676
C	8.235117	0.658651	-3.265773
H	6.408950	-0.446474	-3.637434
C	10.298364	-0.646657	-3.185911
H	10.057520	-2.785241	-3.553076
C	9.633123	0.589921	-3.102944
H	7.730698	1.630534	-3.243880
H	11.387057	-0.694652	-3.083152
H	10.207059	1.508410	-2.940938
C	-1.218114	-1.051330	0.544676
N	-1.974854	-1.191374	-0.353850
C	-2.906031	-1.371943	-1.350125
C	-2.479321	-1.660495	-2.671151
C	-4.287367	-1.293252	-1.033640
C	-3.425487	-1.858069	-3.670634
H	-1.410311	-1.721920	-2.896400
C	-5.226440	-1.519674	-2.032141
H	-4.597995	-1.078987	-0.006627
C	-4.815171	-1.782792	-3.367822
H	-3.107926	-2.092098	-4.690795
H	-6.296228	-1.504895	-1.802667
N	-5.780425	-2.263130	-4.272216
N	-5.980812	-1.946768	-5.471990
C	-5.484148	-0.757782	-6.072974
C	-5.214573	0.453429	-5.388606

C	-5.391659	-0.815636	-7.484411
C	-4.818788	1.577568	-6.120352
H	-5.363533	0.523720	-4.307263
C	-4.952873	0.303173	-8.200154
H	-5.660931	-1.750819	-7.985338
C	-4.670191	1.501293	-7.519576
H	-4.639438	2.526538	-5.603881
H	-4.859742	0.253144	-9.289610
H	-4.361812	2.388708	-8.082297

$[\{Pt_2(tpbz)\}(\textbf{iso-Ph})_4]^{4+}$

G = -6345.219442 Ha

Pt	0.048142	0.059876	4.542811
Pt	0.014542	-0.055167	-4.550690
P	0.222821	-1.562522	2.858237
P	-0.053501	1.647904	2.818468
C	0.028303	-0.686268	1.228565
C	0.110519	0.726267	1.209442
C	-0.092075	-1.394082	0.015409
H	-0.215002	-2.482404	0.029669
C	1.889970	-2.320975	2.810297
C	2.902965	-1.806242	3.651126
H	2.676205	-0.981128	4.336783
C	4.192749	-2.359255	3.617696
H	4.970081	-1.963927	4.279603
C	4.479282	-3.423643	2.747845
H	5.483001	-3.860656	2.729906
C	3.476009	-3.935304	1.905448
H	3.697473	-4.769920	1.232576
C	2.185100	-3.388793	1.930022
H	1.411270	-3.810630	1.279779
C	-1.029194	-2.893789	2.891390
C	-0.706504	-4.179022	3.382315
H	0.321128	-4.421443	3.671629
C	-1.710481	-5.155666	3.488092
H	-1.455585	-6.155089	3.855899
C	-3.032539	-4.855443	3.120169
H	-3.812023	-5.619088	3.209163
C	-3.356706	-3.574075	2.640206
H	-4.387886	-3.338478	2.357723
C	-2.362383	-2.592043	2.526862
H	-2.625513	-1.593694	2.158184
C	1.255127	2.923926	2.829714
C	2.563652	2.574591	2.421699
H	2.774912	1.573452	2.028256
C	3.600642	3.513088	2.522859
H	4.612604	3.240818	2.205947
C	3.343823	4.797661	3.033376
H	4.156319	5.527233	3.112090
C	2.046222	5.145062	3.444668
H	1.843647	6.147519	3.835933
C	1.000409	4.212452	3.351945
H	-0.007909	4.492124	3.674045
C	-1.684922	2.477617	2.742315
C	-2.746364	1.976113	3.529769
H	-2.574838	1.123892	4.198235
C	-4.012858	2.577929	3.467787
H	-4.828032	2.192728	4.088907
C	-4.227762	3.678934	2.622967
H	-5.213224	4.154425	2.583925
C	-3.175883	4.178444	1.834588
H	-3.341009	5.042390	1.182905
C	-1.907615	3.582761	1.887426
H	-1.093597	3.996709	1.282832
P	0.381561	1.537324	-2.874790
P	-0.303411	-1.607603	-2.827597
C	0.154175	0.686455	-1.237795
C	-0.055874	-0.711694	-1.217605
C	0.207476	1.401750	-0.024097
H	0.321176	2.491003	-0.042200
C	-0.728008	2.989400	-2.899218
C	-2.012536	2.849796	-3.473116
H	-2.310935	1.896354	-3.925720
C	-2.897786	3.938518	-3.478621
H	-3.888452	3.830946	-3.932282
C	-2.505956	5.167574	-2.920043
H	-3.192479	6.020435	-2.941474
C	-1.228642	5.309763	-2.349816
H	-0.917080	6.273251	-1.932747
C	-0.336858	4.225534	-2.333849
H	0.668082	4.353610	-1.916712
C	2.115450	2.127771	-2.886564
C	2.480386	3.183780	-3.754085
H	1.720625	3.710439	-4.341012
C	3.825104	3.568821	-3.858336
H	4.101805	4.391920	-4.524994
C	4.812187	2.900253	-3.113988
H	5.861141	3.200521	-3.203658
C	4.453361	1.845036	-2.258730
H	5.222214	1.321029	-1.681398
C	3.110572	1.454195	-2.142804
H	2.843858	0.626738	-1.476406
C	-2.028797	-2.220641	-2.789336
C	-3.021524	-1.524907	-2.062777
H	-2.755211	-0.665338	-1.438067
C	-4.361192	-1.934755	-2.142565
H	-5.128182	-1.393515	-1.578808
C	-4.719373	-3.030995	-2.945006
H	-5.766022	-3.345962	-3.006940
C	-3.734835	-3.721655	-3.672246
H	-4.011115	-4.576605	-4.297726
C	-2.393124	-3.318203	-3.603728
H	-1.635305	-3.862487	-4.176891
C	0.826386	-3.044321	-2.834210
C	2.091773	-2.906828	-3.449469
H	2.361834	-1.966109	-3.944490
C	2.994070	-3.981551	-3.440656
H	3.969565	-3.875922	-3.926511
C	2.638445	-5.194531	-2.825776
H	3.337932	-6.037011	-2.835178
C	1.380043	-5.334821	-2.214259
H	1.096237	-6.286708	-1.753091
C	0.471375	-4.264612	-2.213009
H	-0.519511	-4.392913	-1.763505
C	-0.168101	-1.469617	-5.954879
N	-0.271593	-2.263097	-6.824958
C	-0.404570	-3.174575	-7.849707
C	0.061957	-4.500983	-7.669431
C	-1.002718	-2.764432	-9.071959
C	-0.070931	-5.407317	-8.716854

H	0.530466	-4.792241	-6.724459
C	-1.133872	-3.684108	-10.104075
H	-1.358164	-1.735001	-9.183523
H	0.287548	-6.436773	-8.622221
C	-0.674264	-5.020994	-9.941464
H	-1.593137	-3.407620	-11.057252
N	-0.733538	-6.017405	-10.933026
N	-1.442048	-5.672911	-11.955021
C	-1.474061	-6.599185	-13.000982
C	-0.757041	-7.829017	-13.023976
C	-0.869265	-8.662198	-14.134112
H	-0.132342	-8.093984	-12.166703
C	-2.400975	-7.075062	-15.207386
C	-1.688961	-8.289302	-15.224927
H	-0.322833	-9.610412	-14.165901
H	-3.032736	-6.795960	-16.056188
H	-1.769687	-8.953863	-16.091734
C	0.157564	1.324775	-5.992910
N	0.238796	2.097738	-6.883642
C	0.345787	2.985683	-7.931748
C	-0.102703	4.320091	-7.764911
C	0.900044	2.544031	-9.163702
C	0.004249	5.202786	-8.835213
H	-0.537384	4.635715	-6.811640
C	1.005797	3.440399	-10.218966
H	1.242383	1.509083	-9.264669
H	-0.341509	6.237490	-8.751070
C	0.563967	4.784886	-10.070148
H	1.431291	3.139406	-11.180357
N	0.600133	5.759513	-11.084117
N	1.273010	5.387184	-12.120353
C	1.279245	6.290611	-13.186516
C	0.571518	7.525748	-13.213549
C	0.655177	8.334048	-14.344392
H	-0.023682	7.814282	-12.342997
C	2.139792	6.711375	-15.430681
C	1.437102	7.930982	-15.452086
H	0.115489	9.285999	-14.379498
H	2.742266	6.408879	-16.292661
H	1.495666	8.576194	-16.335140
C	-0.075859	1.476457	5.951139
N	-0.119582	2.261058	6.834027
C	-0.190444	3.160559	7.875199
C	0.409809	4.437024	7.737198
C	-0.859343	2.786764	9.072316
C	0.339215	5.330281	8.801559
H	0.929417	4.697182	6.810249
C	-0.927701	3.693945	10.121354
H	-1.317731	1.795664	9.151208
H	0.800772	6.320431	8.740210
C	-0.333870	4.981511	10.000825
H	-1.438682	3.445593	11.055785
N	-0.321743	5.957968	11.013404
N	-1.097322	5.670967	12.003796
C	-1.064618	6.572997	13.070806
C	-0.218089	7.715158	13.147644
C	-0.277147	8.532204	14.273761
H	0.461675	7.927383	12.318023
C	-2.011126	7.102362	15.256549
C	-1.170963	8.229633	15.327484
H	0.368690	9.413258	14.346844
H	-2.699519	6.877350	16.076901
H	-1.208865	8.880833	16.207266
C	0.114428	-1.326279	5.984110
N	0.126737	-2.095753	6.881339
C	0.159703	-2.977029	7.939366
C	-0.364701	-4.283723	7.776224
C	0.714806	-2.555544	9.178204
C	-0.332852	-5.159142	8.856990
H	-0.796302	-4.581102	6.815906
C	0.745981	-3.445048	10.243894
H	1.116716	-1.541906	9.276066
H	-0.738458	-6.172143	8.776318
C	0.227313	-4.762286	10.098736
H	1.170230	-3.159862	11.210637
N	0.184464	-5.725349	11.122880
N	0.867815	-5.389177	12.164499
C	0.794965	-6.277563	13.240758
C	-0.001666	-7.457225	13.272184
C	0.008254	-8.255562	14.413074
H	-0.605153	-7.712244	12.396882
C	1.594280	-6.732783	15.500746
C	0.803718	-7.897277	15.526513
H	-0.600038	-9.165065	14.451623
H	2.206674	-6.464839	16.367154
H	0.803872	-8.534283	16.417430
C	-2.294876	-6.230101	-14.099928
H	-2.831894	-5.277712	-14.048302
C	2.062064	5.891173	-14.302463
H	2.592974	4.935573	-14.247149
C	-1.959385	6.274106	14.132704
H	-2.595771	5.388381	14.039902
C	1.591063	-5.922897	14.362456
H	2.191456	-5.009588	14.303389

$[\{Pt_2(tpbz)\}(\textit{cis}\text{-}\textbf{iso-Ph})(\textit{trans}\text{-}\textbf{iso-Ph})_3]^{4+}$

G = -6345.199840 Ha

Pt	0.587039	-0.005188	4.357260
Pt	-0.384830	-0.047536	-4.606552
P	0.818035	-1.602746	2.654070
P	0.823940	1.575138	2.637164
C	0.387580	-0.731864	1.069098
C	0.456059	0.681128	1.048180
C	0.034795	-1.432700	-0.102365
H	-0.031920	-2.526771	-0.083202
C	2.544885	-2.187281	2.476299
C	3.420583	-2.059380	3.578903
H	3.074076	-1.581695	4.502039
C	4.737421	-2.535527	3.487449
H	5.410902	-2.434286	4.344579
C	5.189794	-3.134461	2.300590
H	6.217798	-3.504790	2.231777
C	4.325176	-3.254618	1.198532
H	4.678533	-3.717840	0.271650
C	3.006737	-2.782500	1.279059
H	2.347825	-2.881109	0.409674
C	-0.293815	-3.052161	2.759460
C	0.184033	-4.366971	2.568662
H	1.242119	-4.546943	2.352617
C	-0.704658	-5.450823	2.673800

H	-0.331742	-6.470845	2.533605
C	-2.059921	-5.229300	2.968005
H	-2.746147	-6.077916	3.056883
C	-2.535620	-3.919586	3.163139
H	-3.589237	-3.748308	3.406984
C	-1.657816	-2.831380	3.064689
H	-2.026176	-1.813850	3.243049
C	2.512137	2.263894	2.491963
C	3.411673	1.805740	1.504688
H	3.095107	1.065434	0.762560
C	4.724800	2.301417	1.474949
H	5.420171	1.946814	0.707189
C	5.146717	3.245668	2.424879
H	6.172173	3.628154	2.398885
C	4.254183	3.699161	3.412393
H	4.581364	4.435177	4.153813
C	2.941628	3.209072	3.454095
H	2.254352	3.568940	4.227564
C	-0.353775	2.976056	2.708247
C	-1.629601	2.767778	3.280948
H	-1.885669	1.791321	3.709568
C	-2.560109	3.817490	3.317601
H	-3.543400	3.656597	3.771959
C	-2.222529	5.076752	2.789646
H	-2.944229	5.899225	2.833985
C	-0.954982	5.286238	2.219388
H	-0.686962	6.270810	1.822139
C	-0.017878	4.240993	2.173816
H	0.976777	4.418810	1.751236
P	-0.598502	1.551446	-2.901011
P	-0.608955	-1.628502	-2.884240
C	-0.164407	0.678319	-1.318502
C	-0.233388	-0.734595	-1.297406
C	0.189736	1.378981	-0.147102
H	0.258031	2.472939	-0.166569
C	-2.322183	2.142443	-2.718986
C	-3.204864	2.009335	-3.815525
H	-2.866718	1.522417	-4.736854
C	-4.518781	2.492881	-3.721178
H	-5.197071	2.388496	-4.574104
C	-4.961300	3.104492	-2.537071
H	-5.986773	3.481365	-2.466196
C	-4.090003	3.229503	-1.440772
H	-4.435650	3.702695	-0.516041
C	-2.774415	2.750119	-1.524277
H	-2.110183	2.853459	-0.659514
C	0.520212	2.993795	-3.016732
C	0.048148	4.312334	-2.837906
H	-1.008747	4.498984	-2.621827
C	0.941056	5.391165	-2.955989
H	0.572585	6.414100	-2.826009
C	2.294480	5.161031	-3.251468
H	2.983693	6.005938	-3.351480
C	2.764500	3.847502	-3.434320
H	3.816762	3.669479	-3.679143
C	1.882612	2.764001	-3.322753
H	2.246860	1.743301	-3.491375
C	-2.299169	-2.310277	-2.735917
C	-3.195645	-1.844847	-1.749196
H	-2.875089	-1.103636	-1.009710

C	-4.510916	-2.334583	-1.716882
H	-5.204162	-1.974314	-0.949860
C	-4.937796	-3.279881	-2.663581
H	-5.965040	-3.657406	-2.635789
C	-4.048122	-3.740803	-3.650243
H	-4.379333	-4.477602	-4.389073
C	-2.733371	-3.256863	-3.694559
H	-2.048255	-3.622455	-4.467211
C	0.565390	-3.030450	-2.966533
C	1.835259	-2.825035	-3.553428
H	2.088998	-1.849524	-3.985549
C	2.762784	-3.876870	-3.600116
H	3.741250	-3.718482	-4.065580
C	2.428022	-5.135186	-3.068297
H	3.147239	-5.959340	-3.120651
C	1.166358	-5.341821	-2.484162
H	0.900456	-6.325831	-2.084126
C	0.232151	-4.294560	-2.428389
H	-0.758401	-4.470122	-1.995323
C	-0.258301	-1.463637	-6.009252
N	-0.210976	-2.265647	-6.876906
C	-0.174812	-3.194311	-7.893812
C	0.360838	-4.482955	-7.643934
C	-0.676368	-2.841983	-9.176218
C	0.393461	-5.408728	-8.682112
H	0.749626	-4.729847	-6.651441
C	-0.642463	-3.780659	-10.198750
H	-1.090056	-1.841832	-9.340801
H	0.807931	-6.410409	-8.533429
C	-0.111248	-5.080423	-9.966935
H	-1.024272	-3.548800	-11.196918
N	-0.007908	-6.092099	-10.939057
N	-0.629999	-5.806585	-12.033410
C	-0.503696	-6.749514	-13.056761
C	0.279102	-7.936491	-12.980876
C	0.326140	-8.792586	-14.078090
H	0.827334	-8.151299	-12.059662
C	-1.174953	-7.313632	-15.333960
C	-0.398452	-8.485086	-15.253428
H	0.924510	-9.708411	-14.034272
H	-1.732301	-7.085280	-16.247570
H	-0.354210	-9.167288	-16.109087
C	-0.215699	1.352694	-6.020710
N	-0.092115	2.152655	-6.882921
C	0.054047	3.076382	-7.892617
C	-0.131271	4.453833	-7.611021
C	0.380537	2.630912	-9.202547
C	0.005525	5.374258	-8.644797
H	-0.381318	4.774001	-6.595285
C	0.519033	3.563639	-10.221299
H	0.522065	1.562139	-9.392206
H	-0.139566	6.444799	-8.471298
C	0.337614	4.950579	-9.957911
H	0.771613	3.259476	-11.240831
N	0.423020	5.968787	-10.923613
N	0.904812	5.561708	-12.049733
C	0.935083	6.517852	-13.067995
C	0.434207	7.846076	-12.955396
C	0.516644	8.700239	-14.051965
H	-0.005089	8.167763	-12.007293

C	1.592199	6.939928	-15.379758
C	1.094201	8.251526	-15.262833
H	0.134575	9.723743	-13.980255
H	2.037434	6.602911	-16.320846
H	1.154165	8.933457	-16.117749
C	0.432199	1.415429	5.750097
N	0.360320	2.229261	6.605011
C	0.289349	3.178357	7.601704
C	-0.276475	4.446514	7.317461
C	0.784729	2.867820	8.896867
C	-0.345086	5.393822	8.334572
H	-0.659653	4.661258	6.315344
C	0.714064	3.827414	9.898191
H	1.222023	1.882507	9.087961
H	-0.782768	6.381193	8.158985
C	0.151130	5.106853	9.632191
H	1.089521	3.627854	10.905692
N	0.012428	6.139818	10.578585
N	0.601589	5.880475	11.697341
C	0.450578	6.851936	12.690893
C	-0.317341	8.043335	12.557542
C	-0.391272	8.929822	13.628992
H	-0.833789	8.237435	11.613666
C	1.053130	7.472428	14.972682
C	0.291602	8.648314	14.835243
H	-0.978938	9.849377	13.540712
H	1.578205	7.264055	15.909903
H	0.226363	9.354017	15.670244
C	0.397314	-1.406006	5.764857
N	0.214821	-2.215911	6.610711
C	-0.016228	-3.165428	7.571505
C	0.052538	-4.544141	7.227556
C	-0.351895	-2.767630	8.894436
C	-0.235638	-5.503004	8.185935
H	0.314126	-4.835045	6.205779
C	-0.617121	-3.731683	9.857006
H	-0.399665	-1.703836	9.146623
H	-0.224748	-6.566892	7.930950
C	-0.562178	-5.120041	9.521551
H	-0.893584	-3.434274	10.872643
N	-1.117535	-6.080684	10.360212
N	-0.951038	-6.366022	11.567705
C	0.201967	-6.039655	12.319322
C	1.472261	-5.709752	11.779691
C	2.538244	-5.474803	12.650970
H	1.628337	-5.686551	10.697220
C	1.093550	-5.894945	14.581580
C	2.349163	-5.553011	14.047475
H	3.530994	-5.247563	12.248818
H	0.958082	-5.974972	15.664496
H	3.195349	-5.373015	14.718826
C	-1.228511	-6.446194	-14.240259
H	-1.820335	-5.525711	-14.263376
C	1.513308	6.073216	-14.287106
H	1.888286	5.046147	-14.337951
C	1.133345	6.574842	13.905143
H	1.714929	5.650001	13.972552
C	0.026304	-6.167063	13.720282
H	-0.955866	-6.471004	14.095277

[{Pt_2(tpbz)}(*cis*-**iso-Ph**)$_2$(*trans*-**iso-Ph**)$_2$]$^{4+}$

cis groups in gem arrangement

G = -6345.175256 Ha

Pt	0.605826	-0.080009	4.346986
Pt	-0.400440	-0.070562	-4.611383
P	0.833766	-1.668212	2.635428
P	0.854720	1.504387	2.633894
C	0.395776	-0.789407	1.056449
C	0.464559	0.623662	1.043171
C	0.037629	-1.483232	-0.117367
H	-0.028139	-2.577382	-0.104821
C	2.558192	-2.255835	2.442031
C	3.454930	-2.093908	3.522769
H	3.124059	-1.591150	4.438375
C	4.771759	-2.567784	3.418621
H	5.461502	-2.440457	4.259192
C	5.203130	-3.197928	2.240244
H	6.231034	-3.566559	2.161399
C	4.317468	-3.351795	1.159179
H	4.654371	-3.839412	0.238706
C	2.999009	-2.882532	1.252812
H	2.323452	-3.008469	0.399849
C	-0.279927	-3.117624	2.736895
C	0.197980	-4.434697	2.562994
H	1.257740	-4.617956	2.358507
C	-0.692977	-5.516804	2.668931
H	-0.319874	-6.538329	2.540482
C	-2.050856	-5.291418	2.947700
H	-2.739003	-6.138460	3.036780
C	-2.526678	-3.979449	3.126695
H	-3.582450	-3.804820	3.358587
C	-1.646602	-2.893183	3.027206
H	-2.015252	-1.873704	3.193620
C	2.556008	2.161225	2.483575
C	3.419782	1.751041	1.445003
H	3.070906	1.060999	0.669828
C	4.739318	2.229412	1.406478
H	5.407124	1.911285	0.599276
C	5.202541	3.109676	2.397470
H	6.232586	3.479035	2.364171
C	4.345385	3.515656	3.435855
H	4.704977	4.201314	4.209848
C	3.027547	3.041313	3.486924
H	2.368690	3.361796	4.301414
C	-0.292194	2.930788	2.711300
C	-1.583382	2.740355	3.255234
H	-1.868875	1.762259	3.660832
C	-2.490283	3.810275	3.294632
H	-3.485817	3.663240	3.726531
C	-2.113850	5.071858	2.799234
H	-2.818040	5.909324	2.844856
C	-0.830319	5.263874	2.259602
H	-0.532281	6.249599	1.887331
C	0.083577	4.198169	2.210935
H	1.090003	4.360345	1.810604
P	-0.614829	1.515911	-2.894363
P	-0.614753	-1.662941	-2.898127
C	-0.171572	0.634978	-1.319295
C	-0.237265	-0.778060	-1.306818
C	0.188812	1.329119	-0.145493

H	0.255300	2.423372	-0.158340
C	-2.341409	2.096203	-2.702938
C	-3.214585	2.004219	-3.811321
H	-2.868741	1.552373	-4.747432
C	-4.529548	2.483258	-3.710171
H	-5.200495	2.410374	-4.572134
C	-4.982856	3.049560	-2.507679
H	-6.009305	3.422716	-2.431509
C	-4.121210	3.133271	-1.400036
H	-4.475380	3.570725	-0.461021
C	-2.804473	2.657870	-1.490172
H	-2.148085	2.727128	-0.616089
C	0.493842	2.966267	-3.005445
C	0.019957	4.277114	-2.780416
H	-1.032581	4.452283	-2.535143
C	0.904621	5.363369	-2.891456
H	0.534489	6.380677	-2.726699
C	2.251640	5.148216	-3.225302
H	2.934163	5.999162	-3.319946
C	2.723357	3.842507	-3.453875
H	3.770175	3.676636	-3.728748
C	1.849492	2.751701	-3.349736
H	2.213882	1.737592	-3.554019
C	-2.302087	-2.351765	-2.750366
C	-3.199765	-1.890845	-1.762638
H	-2.881283	-1.149729	-1.022147
C	-4.513444	-2.384801	-1.730816
H	-5.207672	-2.028013	-0.963053
C	-4.937580	-3.329858	-2.678998
H	-5.963629	-3.710650	-2.651623
C	-4.046712	-3.786307	-3.666632
H	-4.375769	-4.522899	-4.406628
C	-2.733502	-3.298137	-3.710436
H	-2.047410	-3.660246	-4.483856
C	0.564531	-3.059998	-2.992452
C	1.830902	-2.847042	-3.584075
H	2.078815	-1.868674	-4.013092
C	2.762615	-3.894777	-3.638950
H	3.738409	-3.730405	-4.107919
C	2.435546	-5.156473	-3.110432
H	3.157933	-5.977420	-3.169231
C	1.177363	-5.370642	-2.521430
H	0.917321	-6.357295	-2.124045
C	0.239012	-4.327537	-2.457595
H	-0.748922	-4.509008	-2.020973
C	-0.276267	-1.477864	-6.023163
N	-0.229658	-2.274389	-6.895864
C	-0.194110	-3.196922	-7.918418
C	0.350795	-4.483688	-7.679245
C	-0.705353	-2.840161	-9.195742
C	0.382972	-5.403139	-8.723061
H	0.746913	-4.733978	-6.690507
C	-0.671710	-3.772608	-10.223988
H	-1.126065	-1.841603	-9.352004
H	0.804392	-6.403124	-8.582726
C	-0.131105	-5.070366	-10.002991
H	-1.060736	-3.537250	-11.218543
N	-0.027335	-6.075889	-10.981549
N	-0.655363	-5.786574	-12.071512
C	-0.530097	-6.723440	-13.100594

C	0.256833	-7.908244	-13.034398
C	0.302250	-8.758139	-14.136500
H	0.809506	-8.126288	-12.116606
C	-1.208630	-7.277224	-15.378178
C	-0.428021	-8.446553	-15.307226
H	0.903787	-9.672215	-14.100121
H	-1.770360	-7.045644	-16.288287
H	-0.384966	-9.123868	-16.166819
C	-0.238669	1.338520	-6.017538
N	-0.119491	2.143719	-6.875538
C	0.021442	3.073795	-7.880130
C	-0.164524	4.449276	-7.589382
C	0.343263	2.636764	-9.194076
C	-0.033007	5.376147	-8.618078
H	-0.411087	4.763025	-6.570784
C	0.476486	3.575865	-10.207649
H	0.485362	1.569343	-9.390818
H	-0.178683	6.445432	-8.437463
C	0.294246	4.960942	-9.935115
H	0.725353	3.278282	-11.230030
N	0.374790	5.985378	-10.894703
N	0.849525	5.584878	-12.026240
C	0.876090	6.547906	-13.038078
C	0.379644	7.876713	-12.913267
C	0.457760	8.738193	-14.004411
H	-0.052939	8.193048	-11.960294
C	1.520187	6.984118	-15.350899
C	1.026604	8.296252	-15.221879
H	0.079031	9.762237	-13.923305
H	1.958693	6.652379	-16.297008
H	1.083238	8.983903	-16.072430
C	0.444884	-1.495440	5.742011
N	0.295098	-2.323909	6.576021
C	0.106123	-3.300547	7.519682
C	0.109815	-4.666995	7.125260
C	-0.118503	-2.942314	8.876401
C	-0.133197	-5.652019	8.070126
H	0.284311	-4.927428	6.076991
C	-0.336127	-3.933480	9.823729
H	-0.115805	-1.887353	9.167137
H	-0.173097	-6.705874	7.778864
C	-0.345769	-5.309390	9.438670
H	-0.524629	-3.666155	10.867472
N	-0.862924	-6.286173	10.286008
N	-0.600355	-6.619811	11.464393
C	0.620330	-6.342346	12.124835
C	1.849257	-6.026149	11.490037
C	2.990643	-5.844258	12.274494
H	1.915002	-5.973127	10.399420
C	1.702552	-6.289951	14.306814
C	2.917617	-5.961263	13.678946
H	3.951811	-5.628601	11.796349
H	1.656276	-6.401214	15.394472
H	3.821536	-5.822549	14.281301
C	-1.260622	-6.416000	-14.279487
H	-1.855507	-5.497334	-14.295175
C	1.445593	6.110121	-14.263766
H	1.817395	5.082404	-14.323976
C	0.560090	-6.509589	13.531139
H	-0.394522	-6.802756	13.978878

C	0.440989	1.337257	5.738679
N	0.399167	2.171585	6.579334
C	0.378584	3.164167	7.526671
C	0.035305	4.490613	7.144909
C	0.736530	2.870580	8.869932
C	0.080375	5.506997	8.087040
H	-0.251283	4.700059	6.109757
C	0.757224	3.887530	9.815222
H	0.997988	1.845801	9.151116
H	-0.143015	6.539914	7.803576
C	0.429856	5.227140	9.441771
H	1.050557	3.673608	10.847037
N	0.747922	6.300870	10.270523
N	0.472849	6.568333	11.463172
C	-0.615031	6.014961	12.180043
C	-1.766957	5.419931	11.603844
C	-0.524926	6.194808	13.583141
C	-2.794247	4.979716	12.441852
H	-1.872940	5.350307	10.517320
C	-1.544724	5.717366	14.412185
H	0.356642	6.703005	13.985982
C	-2.680177	5.113921	13.841990
H	-3.701874	4.545623	12.009628
H	-1.470978	5.838982	15.497218
H	-3.495939	4.770682	14.486879

[{Pt_2(tpbz)}(*cis*-**iso-Ph**)$_2$(*trans*-**iso-Ph**)$_2$]$^{4+}$
cis groups in *cis* arrangement
G = -6345.175757 Ha

Pt	0.592992	0.003090	4.335224
Pt	-0.510760	-0.142812	-4.588016
P	0.826290	-1.588235	2.626598
P	0.725612	1.585978	2.605769
C	0.314056	-0.736405	1.056314
C	0.334903	0.676997	1.031394
C	-0.063961	-1.452994	-0.099216
H	-0.090308	-2.549035	-0.077083
C	2.564286	-2.117679	2.393657
C	3.459104	-1.996918	3.481725
H	3.119901	-1.554352	4.424780
C	4.785716	-2.434823	3.350548
H	5.474058	-2.338929	4.196413
C	5.228925	-2.988462	2.138466
H	6.264608	-3.329095	2.038587
C	4.345109	-3.100854	1.051039
H	4.691870	-3.528893	0.104903
C	3.016546	-2.666584	1.170932
H	2.343271	-2.756817	0.311559
C	-0.232639	-3.074727	2.767439
C	0.276976	-4.370348	2.533100
H	1.328148	-4.512508	2.261888
C	-0.569456	-5.484521	2.666591
H	-0.170822	-6.489508	2.492924
C	-1.914276	-5.312226	3.032727
H	-2.566380	-6.184564	3.146015
C	-2.422071	-4.021484	3.270101
H	-3.466066	-3.888543	3.572667
C	-1.586308	-2.903088	3.142822
H	-1.977081	-1.900810	3.356486
C	2.389228	2.320745	2.410553
C	3.266776	1.896717	1.388985
H	2.945062	1.155338	0.650197
C	4.564469	2.428007	1.320447
H	5.242798	2.099397	0.526328
C	4.992776	3.374437	2.265273
H	6.006293	3.784533	2.209160
C	4.122106	3.794559	3.286509
H	4.454232	4.532241	4.024069
C	2.825437	3.268459	3.367195
H	2.155273	3.602137	4.166987
C	-0.488849	2.951892	2.712263
C	-1.747555	2.701251	3.305949
H	-1.967601	1.713928	3.729759
C	-2.706656	3.723323	3.369865
H	-3.676961	3.529806	3.839157
C	-2.414557	4.996917	2.849258
H	-3.159347	5.797154	2.912973
C	-1.163957	5.248443	2.259570
H	-0.931902	6.244028	1.867218
C	-0.198672	4.231046	2.185688
H	0.781932	4.440538	1.745339
P	-0.749578	1.495365	-2.920940
P	-0.913255	-1.654366	-2.834639
C	-0.351580	0.646825	-1.316639
C	-0.403704	-0.767511	-1.282394
C	0.015280	1.362494	-0.159592
H	0.053396	2.457911	-0.186161
C	-2.458795	2.138399	-2.793258
C	-3.131884	2.470824	-3.992780
H	-2.646835	2.310823	-4.961805
C	-4.427194	3.004635	-3.942629
H	-4.943319	3.263483	-4.872760
C	-5.062765	3.198892	-2.704012
H	-6.076832	3.609981	-2.668827
C	-4.400223	2.860973	-1.512594
H	-4.897096	3.007317	-0.548071
C	-3.100256	2.331953	-1.550148
H	-2.597086	2.069435	-0.613843
C	0.389696	2.922786	-3.037039
C	-0.051077	4.229305	-2.731970
H	-1.091657	4.409307	-2.442098
C	0.848482	5.304632	-2.825523
H	0.503481	6.319592	-2.602224
C	2.178917	5.082022	-3.218316
H	2.873642	5.924768	-3.297298
C	2.618010	3.780459	-3.523658
H	3.651528	3.609591	-3.842528
C	1.727645	2.700572	-3.438326
H	2.064666	1.689860	-3.698774
C	-2.682822	-2.083515	-2.652543
C	-3.225277	-2.488666	-1.411268
H	-2.604330	-2.515204	-0.509222
C	-4.576455	-2.857253	-1.329216
H	-4.995361	-3.171464	-0.367750
C	-5.391340	-2.822539	-2.473999
H	-6.445205	-3.110616	-2.403433
C	-4.856753	-2.413224	-3.706709
H	-5.491844	-2.378855	-4.597774
C	-3.507501	-2.040471	-3.800268
H	-3.096013	-1.709441	-4.760272

C	0.065309	-3.201684	-2.874008
C	1.437084	-3.126779	-3.213603
H	1.891992	-2.160445	-3.463098
C	2.208843	-4.296543	-3.260217
H	3.266774	-4.239242	-3.537268
C	1.619311	-5.542641	-2.977010
H	2.221744	-6.455653	-3.027914
C	0.256702	-5.618890	-2.645464
H	-0.205446	-6.589302	-2.436228
C	-0.526042	-4.452535	-2.592975
H	-1.591154	-4.520018	-2.348399
C	-0.183601	1.217665	-6.007053
N	-0.003452	2.008239	-6.868358
C	0.198588	2.934509	-7.867009
C	0.559868	4.261767	-7.523420
C	0.038758	2.544210	-9.224324
C	0.759774	5.187318	-8.542823
H	0.682353	4.539163	-6.472234
C	0.238547	3.481814	-10.228498
H	-0.244723	1.513841	-9.461743
H	1.043947	6.220417	-8.320935
C	0.599206	4.819596	-9.903720
H	0.121238	3.220411	-11.283775
N	0.839065	5.842119	-10.841029
N	0.576845	5.484360	-12.053652
C	0.835123	6.450768	-13.029397
C	1.373340	7.744269	-12.775162
C	1.586978	8.614870	-13.840744
H	1.606990	8.027167	-11.745307
C	0.737932	6.939482	-15.418351
C	1.270909	8.216704	-15.160740
H	2.000389	9.612422	-13.659914
H	0.496233	6.641274	-16.443116
H	1.442758	8.911011	-15.990142
C	0.424577	1.422617	5.726930
N	0.343047	2.237679	6.579771
C	0.259133	3.189224	7.573081
C	-0.354897	4.435856	7.293663
C	0.789231	2.902609	8.859978
C	-0.436725	5.385556	8.307570
H	-0.763817	4.632106	6.297951
C	0.704496	3.864401	9.858081
H	1.263528	1.933825	9.047258
H	-0.911066	6.356528	8.135774
C	0.092982	5.122361	9.596958
H	1.105298	3.683041	10.859234
N	-0.063550	6.154908	10.541115
N	0.552610	5.920367	11.650766
C	0.387108	6.891545	12.642331
C	-0.420717	8.057078	12.516538
C	-0.505281	8.946106	13.585104
H	-0.958761	8.229779	11.580601
C	1.007165	7.542421	14.910926
C	0.206141	8.692628	14.781014
H	-1.123543	9.845919	13.502547
H	1.554099	7.355615	15.840161
H	0.132115	9.400022	15.613851
C	0.497156	-1.398455	5.752087
N	0.369458	-2.211094	6.605409
C	0.202276	-3.164651	7.575466
C	0.314694	-4.541196	7.234307
C	-0.111370	-2.774441	8.906030
C	0.090302	-5.506331	8.203393
H	0.559528	-4.826390	6.206776
C	-0.312591	-3.743878	9.878681
H	-0.192827	-1.712198	9.156119
H	0.135401	-6.570088	7.951605
C	-0.214023	-5.130554	9.546288
H	-0.571871	-3.452950	10.900707
N	-0.709894	-6.108007	10.402711
N	-0.498962	-6.382791	11.605796
C	0.661670	-6.010410	12.323650
C	1.903025	-5.635080	11.747620
C	2.983394	-5.356796	12.587894
H	2.027876	-5.610384	10.661136
C	1.610236	-5.823498	14.559519
C	2.836599	-5.436593	13.989364
H	3.955221	-5.093991	12.157308
H	1.508263	-5.904386	15.646038
H	3.693856	-5.222242	14.636131
C	0.520011	6.057191	-14.357438
H	0.107131	5.056001	-14.516267
C	1.098430	6.642526	13.846208
H	1.710380	5.737092	13.908036
C	0.530311	-6.138913	13.729343
H	-0.428797	-6.478152	14.132694
C	-0.341365	-1.601915	-5.935228
N	-0.197981	-2.456441	-6.743929
C	-0.015481	-3.462887	-7.656211
C	-0.210161	-4.814874	-7.258425
C	0.392985	-3.152948	-8.982038
C	0.025673	-5.835392	-8.166178
H	-0.527362	-5.038159	-6.235356
C	0.605630	-4.176872	-9.894559
H	0.537183	-2.108860	-9.276159
H	-0.083096	-6.882508	-7.868275
C	0.424027	-5.539890	-9.504503
H	0.936595	-3.948176	-10.911686
N	0.922385	-6.579129	-10.283454
N	0.767536	-6.902588	-11.483134
C	-0.331447	-6.514152	-12.284592
C	-1.584693	-6.056820	-11.800926
C	-0.126272	-6.716046	-13.672743
C	-2.600941	-5.771628	-12.715588
H	-1.770272	-5.974505	-10.726021
C	-1.140891	-6.393346	-14.579010
H	0.837220	-7.116692	-14.002641
C	-2.378208	-5.925244	-14.100811
H	-3.582351	-5.445565	-12.355821
H	-0.980943	-6.531030	-15.652755
H	-3.186024	-5.704395	-14.806359

$[\{Pt_2(tpbz)\}(\textit{cis}\text{-}\mathbf{iso\text{-}Ph})_2(\textit{trans}\text{-}\mathbf{iso\text{-}Ph})_2]^{4+}$

cis groups in *trans* arrangement

G = -6345.176055 Ha

Pt	0.756378	-0.049252	4.363145
Pt	-0.364857	-0.176890	-4.559880
P	1.022354	-1.681974	2.694112
P	1.177994	1.466650	2.618274
C	0.619798	-0.830396	1.091963

C	0.687629	0.583505	1.058286
C	0.243300	-1.541171	-0.064464
H	0.186203	-2.635682	-0.036124
C	2.743852	-2.291396	2.568572
C	3.494888	-2.424269	3.759775
H	3.063020	-2.122014	4.720251
C	4.799268	-2.937220	3.710537
H	5.377130	-3.039218	4.634792
C	5.364462	-3.310840	2.479597
H	6.384712	-3.706331	2.444016
C	4.623913	-3.171375	1.293709
H	5.066310	-3.456905	0.333845
C	3.316241	-2.663236	1.331360
H	2.753122	-2.554738	0.398412
C	-0.097119	-3.126016	2.794988
C	0.370969	-4.431015	2.526359
H	1.421669	-4.600561	2.268636
C	-0.516316	-5.517582	2.610553
H	-0.150807	-6.530802	2.413222
C	-1.860894	-5.307836	2.958974
H	-2.545992	-6.158967	3.031471
C	-2.326884	-4.007990	3.228955
H	-3.371754	-3.846966	3.514024
C	-1.449802	-2.917001	3.152298
H	-1.809009	-1.907605	3.386505
C	2.944883	1.916083	2.459394
C	3.525696	2.229884	1.209644
H	2.938907	2.167238	0.286914
C	4.871801	2.621256	1.146507
H	5.321056	2.862908	0.177789
C	5.642441	2.701788	2.318886
H	6.692193	3.007546	2.263207
C	5.068828	2.385396	3.561690
H	5.668965	2.442606	4.475513
C	3.725542	1.988743	3.636435
H	3.285430	1.731238	4.606021
C	0.190236	3.008558	2.654865
C	-1.180222	2.930426	2.998574
H	-1.631156	1.963493	3.253037
C	-1.955872	4.097912	3.041824
H	-3.012754	4.038394	3.322462
C	-1.371152	5.344625	2.750842
H	-1.976354	6.255967	2.799193
C	-0.009706	5.423854	2.414377
H	0.448686	6.394837	2.199726
C	0.776520	4.259757	2.364901
H	1.840929	4.329180	2.117614
P	-0.533628	1.420141	-2.849879
P	-0.471510	-1.758698	-2.828525
C	-0.025162	0.560553	-1.282516
C	-0.066103	-0.852115	-1.256406
C	0.361141	1.272475	-0.126452
H	0.402280	2.368032	-0.148683
C	-2.256420	1.991035	-2.598683
C	-3.169189	1.883620	-3.673221
H	-2.854194	1.426025	-4.617226
C	-4.482880	2.354874	-3.528072
H	-5.184264	2.270031	-4.364329
C	-4.895998	2.928691	-2.314836
H	-5.921443	3.295914	-2.204194

C	-3.994879	3.027551	-1.240358
H	-4.317715	3.471377	-0.293056
C	-2.678999	2.560202	-1.374433
H	-1.992879	2.641255	-0.524492
C	0.558301	2.881165	-3.005493
C	0.077822	4.190245	-2.785655
H	-0.969976	4.358763	-2.516640
C	0.949319	5.283591	-2.929922
H	0.573468	6.299057	-2.766765
C	2.290505	5.076866	-3.291599
H	2.962806	5.932952	-3.410798
C	2.769047	3.772555	-3.515128
H	3.810655	3.612554	-3.812865
C	1.907880	2.674875	-3.377937
H	2.276247	1.661797	-3.580101
C	-2.133242	-2.492793	-2.615756
C	-3.020065	-2.022257	-1.622702
H	-2.703911	-1.249814	-0.913827
C	-4.319925	-2.546495	-1.544400
H	-5.005398	-2.181669	-0.772540
C	-4.741620	-3.531842	-2.451833
H	-5.757037	-3.936160	-2.388609
C	-3.861920	-3.998245	-3.444702
H	-4.188826	-4.766401	-4.152903
C	-2.562533	-3.479968	-3.534663
H	-1.885229	-3.850320	-4.311896
C	0.741402	-3.124992	-2.950434
C	1.979215	-2.883044	-3.589543
H	2.182543	-1.902705	-4.037316
C	2.938161	-3.904466	-3.667125
H	3.892167	-3.717466	-4.171168
C	2.666264	-5.169076	-3.114988
H	3.409941	-5.969389	-3.189983
C	1.436361	-5.412230	-2.479735
H	1.219340	-6.401375	-2.063222
C	0.471834	-4.395356	-2.391931
H	-0.493809	-4.599534	-1.917201
C	-0.285423	-1.602441	-5.954905
N	-0.261223	-2.418794	-6.809859
C	-0.250905	-3.370569	-7.806966
C	0.423904	-4.598294	-7.592312
C	-0.918854	-3.102928	-9.032245
C	0.427630	-5.548460	-8.609289
H	0.939022	-4.779938	-6.644235
C	-0.911084	-4.065352	-10.033366
H	-1.437956	-2.148851	-9.169512
H	0.945093	-6.504837	-8.487294
C	-0.240611	-5.304747	-9.836566
H	-1.419219	-3.898860	-10.987293
N	-0.154820	-6.335772	-10.791700
N	-0.893319	-6.123722	-11.828707
C	-0.806456	-7.092845	-12.832312
C	0.045359	-8.232883	-12.794483
C	0.040848	-9.122587	-13.865856
H	0.686482	-8.386073	-11.922234
C	-1.648274	-7.769993	-15.020332
C	-0.803219	-8.894919	-14.977738
H	0.691492	-10.003031	-13.850553
H	-2.298142	-7.603017	-15.884801
H	-0.798317	-9.602696	-15.813524

C	0.576336	1.411464	5.707202
N	0.484317	2.265336	6.520536
C	0.383635	3.264534	7.462370
C	0.364175	4.618403	7.042004
C	0.306123	2.921853	8.839613
C	0.267244	5.618084	8.004761
H	0.422860	4.859535	5.976470
C	0.210349	3.932181	9.786816
H	0.326596	1.868657	9.137288
H	0.249564	6.675205	7.722841
C	0.189822	5.297161	9.384679
H	0.150609	3.707950	10.855355
N	0.095732	6.398004	10.257669
N	0.039254	6.049306	11.499991
C	-0.057572	7.104864	12.410397
C	-0.096294	8.484511	12.059955
C	-0.194056	9.440993	13.067199
H	-0.047639	8.763695	11.004091
C	-0.216216	7.681749	14.776392
C	-0.254140	9.044065	14.423554
H	-0.224417	10.505311	12.812193
H	-0.263081	7.385072	15.828663
H	-0.330667	9.806058	15.206506
C	0.398072	-1.410025	5.772938
N	0.149513	-2.202161	6.619034
C	-0.159662	-3.136087	7.573060
C	-0.147893	-4.517658	7.233064
C	-0.517014	-2.719257	8.884385
C	-0.512797	-5.458571	8.183034
H	0.131304	-4.824190	6.220529
C	-0.859505	-3.666340	9.839220
H	-0.518935	-1.653893	9.134147
H	-0.545845	-6.522422	7.929765
C	-0.862378	-5.056741	9.507329
H	-1.152091	-3.353099	10.845531
N	-1.490691	-5.986637	10.328649
N	-1.378506	-6.275679	11.541693
C	-0.236472	-6.003271	12.330462
C	1.065381	-5.735790	11.833024
C	2.111810	-5.549204	12.738978
H	1.258310	-5.723244	10.756326
C	0.585535	-5.894757	14.621022
C	1.873001	-5.614296	14.128555
H	3.127041	-5.370376	12.369887
H	0.410601	-5.965175	15.698936
H	2.704035	-5.472571	14.827431
C	-1.651122	-6.869578	-13.952080
H	-2.293598	-5.983387	-13.947444
C	-0.118139	6.713177	13.774579
H	-0.085622	5.644269	14.007915
C	-0.464297	-6.118224	13.725026
H	-1.471754	-6.373968	14.067501
C	-0.317467	1.220481	-5.983478
N	-0.308061	2.026686	-6.852357
C	-0.333483	2.968961	-7.847214
C	-0.299467	4.350426	-7.508882
C	-0.431897	2.561090	-9.205644
C	-0.394030	5.302210	-8.511969
H	-0.216758	4.649352	-6.459625
C	-0.501767	3.517531	-10.208725
H	-0.448658	1.495263	-9.452984
H	-0.412271	6.368691	-8.268499
C	-0.484339	4.908827	-9.881076
H	-0.594351	3.212355	-11.254989
N	-0.865173	5.869043	-10.812322
N	-0.492473	6.128724	-11.978937
C	0.760682	5.762656	-12.523477
C	1.915100	5.413191	-11.775976
C	0.822529	5.867431	-13.935904
C	3.103453	5.135223	-12.455049
H	1.888946	5.406700	-10.682365
C	2.009642	5.552920	-14.604375
H	-0.075734	6.187537	-14.472866
C	3.149868	5.190609	-13.864631
H	4.009441	4.890839	-11.890774
H	2.057093	5.615095	-15.695860
H	4.089877	4.976225	-14.383
